# Visible-Light Active Titanium Dioxide Nanomaterials with Bactericidal Properties

**DOI:** 10.3390/nano10010124

**Published:** 2020-01-09

**Authors:** Chengzhu Liao, Yuchao Li, Sie Chin Tjong

**Affiliations:** 1 Department of Materials Science and Engineering, Southern University of Science and Technology, Shenzhen 518055, China; 2 Department of Materials Science and Engineering, Liaocheng University, Liaocheng 252000, China; liyuchao@lcu.edu.cn; 3 Department of Physics, City University of Hong Kong, Tat Chee Avenue, Kowloon, Hong Kong 999077, China

**Keywords:** antibacterial activity, photocatalyst, titania, nanomaterial, doping, *Staphylococcus aureus*, *Escherichia coli*, reactive oxygen species, silver nanoparticle, visible light

## Abstract

This article provides an overview of current research into the development, synthesis, photocatalytic bacterial activity, biocompatibility and cytotoxic properties of various visible-light active titanium dioxide (TiO_2_) nanoparticles (NPs) and their nanocomposites. To achieve antibacterial inactivation under visible light, TiO_2_ NPs are doped with metal and non-metal elements, modified with carbonaceous nanomaterials, and coupled with other metal oxide semiconductors. Transition metals introduce a localized d-electron state just below the conduction band of TiO_2_ NPs, thereby narrowing the bandgap and causing a red shift of the optical absorption edge into the visible region. Silver nanoparticles of doped TiO_2_ NPs experience surface plasmon resonance under visible light excitation, leading to the injection of hot electrons into the conduction band of TiO_2_ NPs to generate reactive oxygen species (ROS) for bacterial killing. The modification of TiO_2_ NPs with carbon nanotubes and graphene sheets also achieve the efficient creation of ROS under visible light irradiation. Furthermore, titanium-based alloy implants in orthopedics with enhanced antibacterial activity and biocompatibility can be achieved by forming a surface layer of Ag-doped titania nanotubes. By incorporating TiO_2_ NPs and Cu-doped TiO_2_ NPs into chitosan or the textile matrix, the resulting polymer nanocomposites exhibit excellent antimicrobial properties that can have applications as fruit/food wrapping films, self-cleaning fabrics, medical scaffolds and wound dressings. Considering the possible use of visible-light active TiO_2_ nanomaterials for various applications, their toxicity impact on the environment and public health is also addressed.

## 1. Introduction

The overuse of antimicrobials in humans, animal husbandry and aquafarming gives rise to the development of dangerous, antibiotic-resistant bacteria [1,2]. Infections caused by antibiotic-resistant bacteria are now emerging as worldwide public health challenges. Medicines find it harder to treat infections, increasing the risk of mortality and morbidity. For instance, Staphylococci such as *Staphylococcus aureus* (*S. aureus*) and *Staphylococcus epidermidis* (*S. epidermidis*), that cause orthopedic infections (e.g., osteomyelitis), have developed into methicillin-resistant *S. aureus* (MRSA) and methicillin-resistant *S. epidermidis* (MRSE). MRSA is capable of forming biofilms on medical devices, giving rise to antibiotic resistance [3,4]. Osteomyelitis is a bone infection induced by *Staphylococci,* leading to progressive bone loss and tissue damage. Moreover, multidrug-resistant (MDR) bacteria spread not only between hospital inpatients, but also through food chains and potable water [5]. Accordingly, researchers have concentrated on developing antimicrobial nanomaterials as alternatives to conventional antibiotics [6,7,8,9].

Current developments in nanoscience and nanotechnology have led to the creation of advanced functional nanomaterials with unique chemical, physical, and biological properties [9,10,11,12,13,14,15,16,17]. Nanomaterials with large, specific surface area-to-volume ratios enhance surface chemical reactivity due to the size reduction at the nanoscale. Thus, nanomaterials have opened up new opportunities for developing bactericidal agents to treat deadly microbial infections [18]. In particular, metal and metal oxide nanoparticles (NPs) have attracted great attention as promising candidates for antibacterial agents [19,20]. The main mechanisms of the antibacterial activities of those nanoparticles proposed in the literature include: (a) oxidative stress induction associated with the generation of reactive oxygen species (ROS) [21], where the oxidation process in bacterial cells causes peroxidation of the lipid membrane, thereby damaging proteins and DNA; (b) released metal ions from metal or metal oxide NPs penetrating through bacterial cell walls, directly interacting with the –SH, –NH and –COOH groups of nucleic acid and protein and eventually causing cell death [15,22]. For example, silver nanoparticles (AgNPs) have been employed as antibacterial agents for textile fabrics, healthcare products, cosmetics, coatings and wound dressings, because they exhibit relatively high bactericidal activity [15,23,24,25,26,27]. However, AgNPs are toxic for several human cell lines. This is because they induce a dose-, size- and time-dependent cytotoxicity, especially those with sizes of ≤10 nm [15].

Compared to other types of nanoparticles, titanium dioxide is particularly attractive for photocatalytic bactericidal activity because of its relatively low cost, natural abundance and superior chemical stability. Titanium dioxide (TiO_2_), generally known as titania, is an n-type semiconductor due to the presence of oxygen vacancies [28,29]. Those oxygen vacancies favor the formation of unpaired electrons or Ti^3+^ centers, thus acting as electron donors in the electronic structure of TiO_2_ [28]. Furthermore, oxygen vacancies can influence the charge transport and electron–hole recombination processes by trapping charge carriers in the defect sites [30,31,32,33]. Titania also has a high dielectric permittivity (κ = 50–80) that finds application as a gate insulator in the microelectronic industry. However, TiO_2_ with a bandgap of 3.2 eV suffers from a large leakage current and low dielectric breakdown field. In contrast, HfO_2_ with a larger bandgap (5.3–5.7 eV) is widely used as a high-κ gate dielectric material in the microelectronic sector [34].

**Figure 1:**
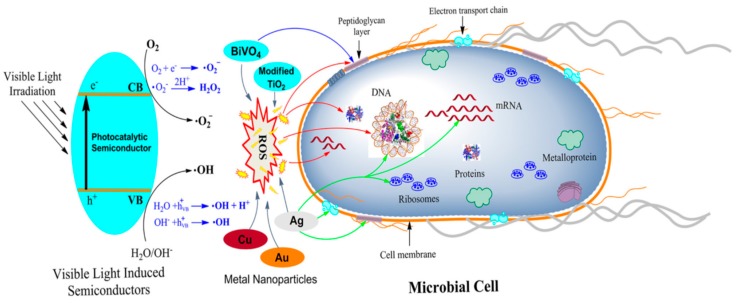
The possible mechanisms of antibacterial activities exhibited by different metal nanoparticles (NPs) and photocatalytic semiconductors. The activation of the photocatalytic semiconductor by visible light is depicted on the left-hand side of the figure. Reactive oxygen species created by various semiconductors destruct bacterial cell components, as indicated by red arrows. Ag, Cu, and Au nanoparticles also generate reactive oxygen species (ROS) for bacterial killing. The green arrow represents targets of Ag. Reproduced with permission from [22]. Copyright Frontiers, 2018.

By irradiating photocatalytic semiconductors with a photon of sufficient energy (≥band gap energy), an electron in the valence band (VB) is excited to the conduction band (CB), leaving a positive hole in the VB. These charge carriers migrate to the photocatalyst surface and can generate highly reactive oxygen species (ROS) such as hydroxyl (^•^OH) and superoxide anion (O_2_^−^) radicals, and hydrogen peroxide (H_2_O_2_) through the oxidative or reductive path with surface-adsorbed water and oxygen (Figure 1). Hydroxyl and superoxide species are highly reactive due to the presence of unpaired valence shell electrons, and can cause oxidative damage to biomolecules such as proteins, lipids and nucleic acids [25,35,36].

Matsunaga et al. first reported the antimicrobial and photoelectrochemical activities of platinum-loaded titanium oxide (TiO_2_/Pt) powders for killing Lactobacillus acidophilus, *Saccharomyces cerevisiae* and *Escherichia coli* (*E. coli*) in 1985 [37]. Nano-TiO_2_ exhibits excellent photocatalytic bactericidal activity against viruses and MDR bacteria under UV irradiation [38]. Accordingly, extensive efforts have been carried out by researchers to improve the photocatalytic bactericidal activity of TiO_2_ nanomaterials. TiO_2_ nanostructures have a wide spectrum of industrial, environmental and energy applications, including water purification, food preservation, degradation of dyes, chemical sensors, dye-sensitized solar cells, and antimicrobial agents. [39,40,41,42,43,44,45,46,47,48,49,50,51,52,53,54,55,56,57]. In particular, visible light-responsive TiO_2_ doped with metals and non-metals exhibit bactericidal activity against a wide variety of bacterial species including Gram-negative *E. coli*, Acinetobacter baumannii, Shigella flexneri, and Gram-positive *S. aureus*, Bacillus subtilis, Listeria monocytogenes, as well as Bacillus anthracis spores [58]. Those photocatalysts can be used for the disinfection of pathogenic bacteria, thereby preventing the spread of microbe-related diseases. Recently, Markov and Vidaković reviewed antimicrobial testing methods of TiO_2_ photocatalysts, including thin-film technique, petri-dish system, and polytetrafluoroethylene membrane-separated system. They also addressed the calculation methods for assessing the antimicrobial efficacy of TiO_2_ photocatalysts [35]. To avoid mechanical damage to TiO_2_ NPs, they are embedded in the polymeric matrices to form antibacterial nanocomposites [59,60,61,62,63,64,65]. The beneficial effects of polymers as the matrix materials of functional composites include ease of processing and good moldability, and they are inexpensive with a low density [66,67,68,69,70,71,72].

Apart from bactericidal activity, TiO_2_ NPs also find attractive application in biomedical fields as photodynamic therapeutic agents for destroying human cancer cells from the skin to the internal organs under ultraviolet (UV) and visible light illumination [36]. This is due to the ROS created by TiO_2_. NPs can damage cellular respiration in mitochondria, thus releasing electron-transfer proteins and causing cell death. Moreover, light-activated TiO_2_ NPs can lead to DNA fragmentation as a result of the electron transfer mechanism. This approach shows promise for reprograming gene-coding either by deleting or by inserting gene codons. In addition, TiO_2_ nanotubes can be used for light-controlled delivery of drugs for treating the diseased tissues upon UV irradiation [36]. This article provides an update review on the current development, synthesis, photocatalytic bacterial inactivation, and cytotoxicity of TiO_2_ NPs and their nanocomposites, especially in a rapidly growing field of research, over the past five years.

## 2. Crystal Structure of Titania

**Figure 2:**
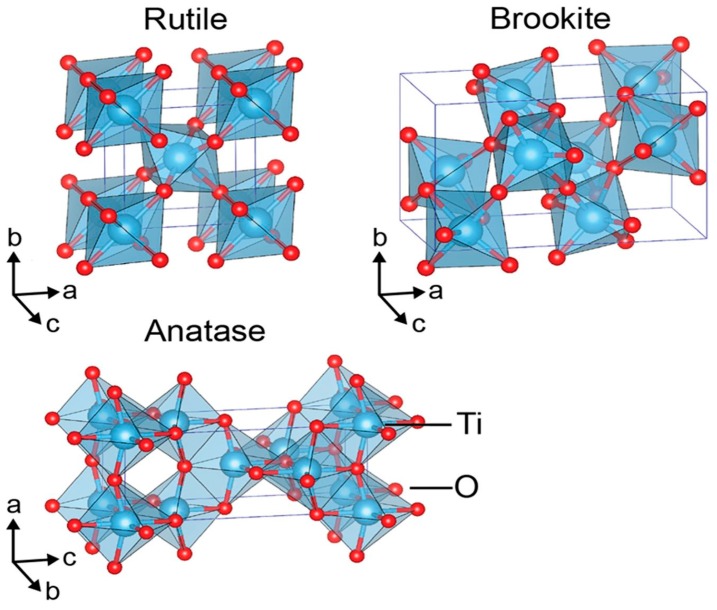
Connecting the chains of distorted TiO_6_ octahedra by sharing edges and corners in different ways to form rutile, brookite and anatase polymorphs. Titanium atoms are blue; oxygen atoms are red. Reproduced with permission from [42]. Copyright Nature Publishing Group, 2017.

Titanium dioxide generally exists naturally in three crystalline structures, i.e., anatase, rutile, and brookite [42,43]. Anatase exhibits the tetragonal structure with a space group of I4_1_/amd (I: body centered). Body-centered tetragonal anatase has lattice parameters of a = 3.7845 Å and c = 9.5143 Å. Rutile belongs to the P42/mnm (P: primitive) space group, with the primitive tetragonal lattice having lattice parameters a = 4.5937 Å, and c = 2.9587 Å. Brookite is orthorhombic with a space group of Pbca, having lattice parameters of a = 9.1819 Å, b = 5.4558 Å, and c = 5.1429 Å, as shown in Figure 2. [43,73,74]. These polymorphs are formed by linking the chains of distorted TiO_6_ octahedra through corner- and edge-sharing in different ways. In the TiO_6_ octahedra, titanium cations (Ti^4+^) are coordinated to six oxygen anions (O^2−^). The octahedron shares two, three, and four edges with adjacent octahedra to give rutile, brookite and anatase, respectively [42]. Anatase and brookite are metastable, and transform irreversibly to a stable rutile phase by heating at 500–700 °C. Moreover, anion fluorine dopant also stabilizes anatase at elevated temperatures (>1000 °C) [75]. Generally, anatase TiO_2_ is more photoactive than rutile and brookite. Anatase TiO_2_ absorbs ultraviolet light (UV) to create an electron–hole pair necessary for photocatalytic reaction. In the process, electron is excited from the valence band to the conduction band, leaving a positively charged hole in the valence band. This photogenerated electron–hole pair displays a high reducing and oxidizing capability. In this respect, the electron in the conduction band reacts with molecular oxygen to produce superoxide ion (O_2_^−^) via a reductive process, while the hole in the valence band oxidizes adsorbed water or hydroxyl ions at the titania surface into hydroxyl radicals (^•^OH) [76]. The photocatalytic activity of TiO_2_ depends mainly on the crystal structure, shape, particle size and surface area. The equilibrium shape of anatase consists of a truncated bipyramid constructed by {101} and {001} facets. According to the Wulff construction, the {001} facets constitute nearly 6% of the total exposed surface of anatase TiO_2_, while stable {101} facets contribute to more than 94% of the surface area [42]. However, the {001} facets of anatase TiO_2_ have a higher photocatalytic performance than {101} facets [77,78,79]. TiO_2_ NPs with a larger surface area and smaller size than their bulk counterparts generate more ROS during photoexcitation [80]. Xu et al. indicated that anatase TiO_2_ NPs exhibit a higher phototoxicity and cytotoxicity in human keratinocyte cells than rutile TiO_2_ NPs [81]. Recently, Bartlet et al. indicated that one-dimensional titania nanotubes prepared by electrochemical anodization exhibit superhydrophobic behavior with a large water contact angle of >150°. Such superhydrophobic titania nanotubes reduced bacterial adhesion on their surfaces [82].

## 3. Visible-Light Active TiO_2_

TiO_2_ NPs with a large bandgap (anatase = 3.2 eV and rutile = 3.0 eV) can only be activated by UV light, which accounts for less than 5% of the solar spectrum compared to 45% of visible light [83]. The low photocatalytic efficiency of titania under visible light limits its practical applications. Extending the utilization of solar energy to the visible region has motivated researchers to improve the visible-light photocatalytic performance of TiO_2_ NPs. Moreover, TiO_2_ NPs have another drawback, due to a rapid recombination of photogenerated electron–hole pairs. Recombination occurs when the excited electron returns to the valence band without interacting with the adsorbed species under UV irradiation. Accordingly, the energy of recombination is dissipated in the form of light or heat. Therefore, it deems necessary to enhance the photocatalytic activity of TiO_2_ NPs by reducing both the bandgap and the recombination of electron–hole pairs under visible light irradiation. Many attempts have been made by researchers to design and synthesize visible light-active TiO_2_ photocatalysts. These include metal and non-metal doping, coupling with semiconductors, and modification with graphene oxide or carbon nanotube [84,85,86,87,88,89,90,91,92,93,94,95,96,97,98,99,100,101,102,103,104]. The incorporation of those dopants into titania affects its electronic band structure greatly, thereby promoting visible light absorption and a red shift in the bandgap.

### 3.1. Metal Doping

The VB of titania is composed of hybridized states of O-2p and Ti-3d orbitals, while the CB consists of primarily Ti-3d orbitals. The electronic and optical properties of titania can be modified by doping. In this context, titanium or oxygen ions’ sites of titania lattice can be substituted with either metal or nonmetal dopants to alter their optical and photocatalytic properties. The cationic doping of titania with transition metals, rare earth metals and noble metals is typically used to improve its photocatalytic performance under visible light excitation. The presence of metal ion dopants can alter the charge transfer properties of TiO_2_, thus improving the separation efficiency of photogenerated carriers, and producing a shift in its absorption edge to the visible regime. The dopant energy level is located below the CB of TiO_2_, acting as an electron or hole trap, and thus allowing more carriers to transport to the surface. The photocatalytic activity of metal-doped titania depends on several factors, including the dopant concentration, type of metal dopant, d-electron configuration and energy band level of dopant in the titania lattice [105]. Although metal dopants facilitate a red shift in optical absorption edges of titania, they can induce defect states, acting as carrier recombination centers, especially at very high dopant contents. Thus, the occurrence of a rapid recombination rate of photogenerated charge carriers arises from a reduction in the distance between the trapping sites by increasing the number of dopant ions.

Doping TiO_2_ with transition metals influences its electronic energy levels and narrows the bandgap, resulting in a shift in the absorption spectrum of titania to longer wavelengths. Titania can be self-doped with Ti^3+^ ions to improve its visible-light absorption and avoid the incorporation of other impurities into its lattice. The introduction of Ti^3+^ energy level and the creation of an oxygen vacancy (O_vac_) in the bandgap are responsible for the shift in optical adsorption of TiO_2_ into the visible light region. As such, the electrons in the VB can be excited to the O_vac_–Ti^3+^ defect states, and electrons from these defect sites can be excited to the CB upon visible light illumination [106,107]. In this respect, the O_vac_–Ti^3+^ sites can trap photogenerated electrons under visible light, thereby inhibiting the recombination of electron–hole pairs and improving photocatalytic activity accordingly. Generally, oxygen vacancy is not stable in air, and it remains a challenge to develop a stable Ti^3+^ self-doped titania with a high photocatalytic performance [108]. 

**Figure 3:**
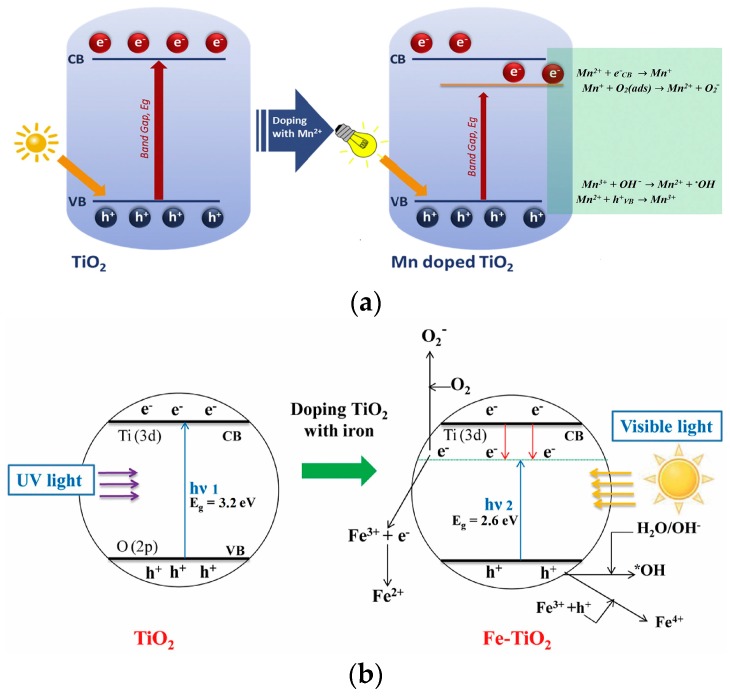
The charge transfer processes between excited electrons from the valence band of TiO_2_ with (**a**) Mn^2+^ ions of Mn-doped TiO_2_, and (**b**) Fe^3+^ ions of Fe-doped TiO_2_. CB and VB are the conduction and valence bands of TiO_2_, respectively. Reproduced with permission from [92,95], respectively. Copyright Elsevier, 2017; Copyright American Chemical Society, 2013.

Apart from Ti^3+^ ions, other transition metals, such as copper (Cu), vanadium (V), chromium (Cr), manganese (Mn), iron (Fe) and nickel (Ni), are typically employed to enhance the visible-light photocatalytic activity of titania [88,89,90,91,92,93,94,95,109,110,111,112,113,114,115]. The redshift effectiveness takes the following order: V > Cr > Mn > Fe > Ni [86]. The substitution of Ti^4+^ in the TiO_2_ lattice by transition metal ions creates a new energy state in the bandgap of TiO_2_. Therefore, the localized d-electron state of transition metals introduced in the bandgap captures the excited electrons from the titania valence band, thereby suppressing the recombination of charge carriers. Figure 3a shows the typical charge transfer reactions involved during photocatalysis of Mn-doped TiO_2_. Mn^2+^ displays an electronic configuration of 3d^5^ and changes to 3d^6^ (Mn^+^) by trapping electrons, while it changes to 3d^4^ (Mn^3+^) as it traps the holes. Both Mn^+^ and Mn^3+^ species are unstable, and react with adsorbed O_2_ and surface hydroxyl molecules to yield ROS [92]. Similarly, Fe^3+^ ions of Fe-doped TiO_2_ can also act as hole and electron traps in prohibiting the recombination of the electron–hole pair and promoting ROS generation [84,85,86]. These result in a red shift in the absorption edge and thus enhance photocatalytic activity (Figure 3b) [95]. Doping TiO_2_ with vanadium, molybdenum (Mo) and tungsten can also shift its absorption edge to the visible region [111,112]. By doping TiO_2_ NPs with 1% and 2% Mo, the bandgap of TiO_2_ NPs decreases from 3.05  to 2.94 and 2.73  eV, respectively. The ionic radius of Mo^6+^ is 0.062 nm, while that of Ti^4+^ is 0.068 nm. As such, Mo ions can readily replace Ti^4+^ in the TiO_2_ lattice, as they have approximately the same ionic radii, resulting in a narrower bandgap [112]. This facilitates the charge transfer between the VB and Mo-3d orbitals, thereby promoting photocatalytic activity under visible light [112].

From the literature, rare earth metal ions are effective in extending the recombination time of charge carriers and improving their separation efficiency. Rare earth metals, such as cerium (Ce), lanthanide (La), erbium (Er) and ytterbium (Yb), with 4f, 5d, and 6s electrons are good dopants for modifying the electronic structure and optical properties of titania [116,117]. Rare earth dopants introduce several impurity energy levels due to the introduction of orbitals between the conduction and valence bands. Moreover, lattice defects are generated in titania as a result of a large mismatch of both the charge and ionic radius between the dopant and Ti cations. The impurity energy levels act as trapping centers for photogenerated electrons and holes, thereby favoring charge separation and reducing the electron–hole recombination [118,119,120,121]. Among these, the La dopant in titania is studied most frequently, followed by Ce doping, in recent years [116,117,118,119,120,121,122]. Kasinathan et al. reported that cerium doping suppresses the recombination of photogenerated electron–hole pairs in titania and promotes a red-shift in its band gap transition. As such, Ce-TiO_2_ had strong antibacterial activity against *E. coli* due to its strong oxidation activity and superhydrophilicity [121]. 

Generally, two or more types of metal cations can be incorporated into the TiO_2_ lattice to further improve its photocatalytic performance. This is typically termed the ‘co-doping’. The enhancement in the photocatalytic activity of co-doped TiO_2_ is attributed to the synergistic effect of the dopants in increasing visible light absorption, thus facilitating electron–hole generation and suppressing the recombination rate [90,113,114,115]. Very recently, Aviles-Garcia et al. synthesized W and Mo co-doped TiO_2_, and reported that the nanocomposite with the W:Mo = 1:1 ratio having a bandgap of 2.87 eV exhibits a synergistic effect between the dopants to generate more hydroxyl radicals for degrading 4-chlorophenol. This is because both the W6^+^ and Mo^6+^ ions are effective in trapping photogenerated electrons, thus extending the lifetime of electron–hole pairs and reducing their recombination rate. The holes can react with the adsorbed H_2_O or –OH groups on the TiO_2_ surface, giving rise _to_ hydroxyl radicals. The photocatalytic reactions can be expressed as follows [114]

W/Mo-TiO_2_ + *hv* → e^−^ + h^+^ (1)

W^6+^ + e^−^ → W^5+^ (2)

Mo^6+^ + e^−^→ Mo^5+^ (3)

H_2_O + h^+^ → ^•^OH + H^+^ (4)

OH^−^ + h^+^ → ^•^OH. (5)

The visible light response of titania can also be achieved by doping with noble metals such as gold (Au), silver (Ag), platinum (Pt) and palladium (Pd) [123,124,125,126]. As recognized, a collective oscillation of conduction electrons can be induced in metal NPs by irradiating with light. This is because the collective oscillation of surface electrons resonates with the electromagnetic field of the incident light. This behavior is generally termed as the localized surface plasmon resonance (LSPR). LSPR covers a wide range of solar spectrum, particularly in the visible and near-infrared (NIR) regions [127,128]. After excitation, LSPR decays non-radiatively into hot electrons and holes through Landau damping, generating highly energetic charge carriers that are typically termed ‘hot carriers’ [129]. This ultrafast relaxation renders the hot carriers capable of rapidly separating and transferring into semiconductors to drive chemical reactions on adsorbed molecules [128,129,130,131]. The LSPR effect is more pronounced for Au and Ag nanoparticles compared with other metals. 

**Figure 4:**
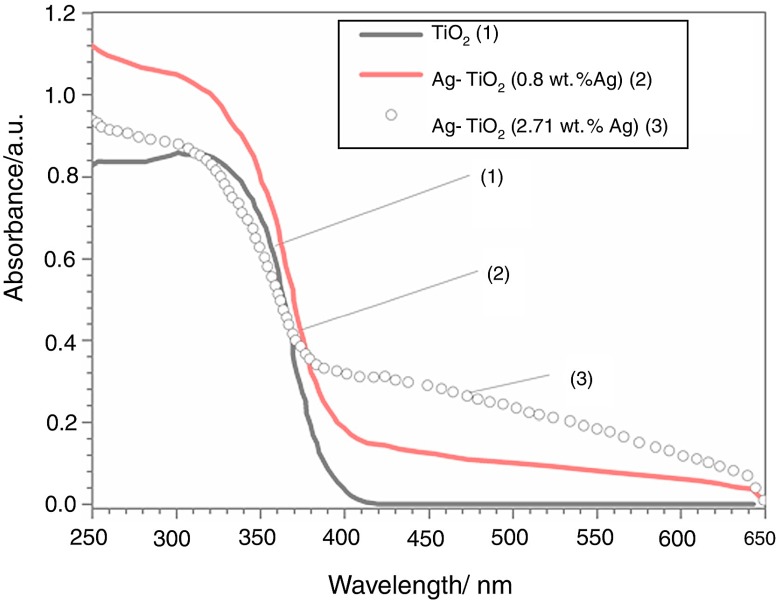
Ultraviolet-visible diffuse reflectance spectra of TiO_2_ and its nanocomposites. Reproduced with permission from [126]. Copyright Elsevier, 2019.

Employing plasmonic NPs on semiconductors is considered to be effective in improving their photocatalytic performance. In this respect, noble metal NPs act as electron donors for titania by injecting hot electrons into the conduction band of TiO_2_ under visible light [132]. The holes created in plasmonic AgNPs can capture conduction electrons of TiO_2_, thereby reducing the charge recombination in titania. Therefore, plasmonic oscillation from Au and Ag nanoparticles to TiO_2_ under visible light has received considerable attention in recent years [133,134,135,136,137,138]. Moreover, AgNPs with well-established antibacterial properties are particularly attractive dopants for titatia in addition to their LSPR effect [15]. Figure 4 shows the UV-visible spectra of pristine TiO_2_ and Ag/TiO_2_ nanocomposites with different AgNP contents [126]. Pristine TiO_2_ exhibits a strong UV light absorption band due to the excitation of the electron–hole pair across the bandgap. The Ag/TiO_2_ nanocomposites display higher absorption values in the UV region, and the absorption intensity increases with increasing AgNP concentrations. The introduction of AgNPs into TiO_2_ results in an increase in the absorption towards the visible light region, i.e., 400–650 nm wavelength. This arises from the LSPR effect of AgNPs that promotes the absorption of Ag/TiO_2_ nanocomposites in the visible regime. 

**Figure 5:**
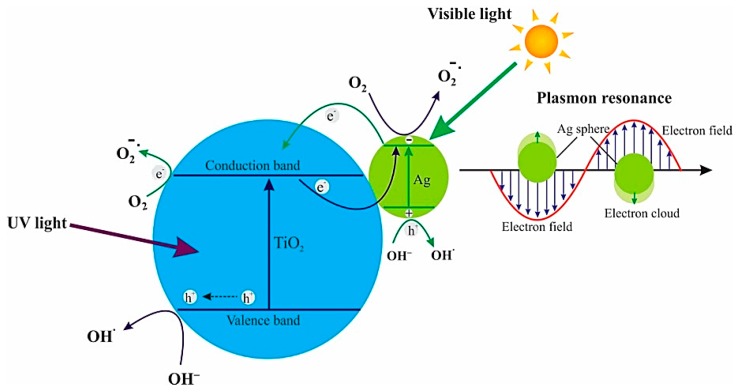
The creation of reactive oxygen species in Ag/TiO_2_ nanomaterials due to the localized surface plasmon resonance (LSPR) effect of AgNPs under visible light. After excitation, LSPR decays into hot electrons and holes through Landau damping, creating highly energetic charge carriers. On the other hand, AgNPs serve as an excellent electron accumulator for TiO_2_ under UV irradiation. Reproduced with permission from [138]. Copyright MDPI, 2019.

When a metal comes into contact with a semiconductor of a different work function, a large potential barrier is established at their interface, which is usually known as the Schottky barrier [137]. Such a barrier at the AgNPs/titania junction improves the charge separation or suppresses the charge recombination greatly [135,138]. Under UV irradiation and in the absence of plasmonic oscillation, electron transfer from the TiO_2_ conduction band to AgNPs is thermodynamically favorable, as the Fermi level of titania is higher than that of AgNPs. From this perspective, excited electrons are transferred from titania to AgNPs across the Schottky barrier at the Ag/TiO_2_ interface. Accordingly, AgNPs serve as an excellent electron accumulator, thus suppressing the charge recombination process. Under visible light irradiation, AgNPs experience the LSPR effect, and excite the conduction electrons for transfer to TiO_2_ to create ROS, as mentioned previously (Figure 5). 

### 3.2. Carbonaceous Nanomaterials Modified Titania

Pure and high-quality graphene is an excellent electrical conductor as it has no bandgap. Graphene consists of a monolayer of sp^2^-bonded carbon atoms that are tightly organized into a two-dimensional (2D) honeycomb structure. Graphene has been reported to possess an excellent electrical mobility of 2 × 10^5^ cm^2^ V^−1^ s^−1^, a superior light transparency of 97.7%, a high specific surface area of 2600 m^2^ g^−1^, and good antibacterial activity [14,139,140]. In this context, graphene and its derivatives, such as graphene oxide (GO) and reduced graphene oxide (rGO), find attractive applications in electronic and optoelectronic devices, energy storage devices, chemical sensors and biomedical implants [141,142,143]. Moreover, a graphene sheet with a lateral dimension of several micrometers can serve as a template for anchoring TiO_2_ NPs onto its surface [102,144,145,146]. 

Large-area graphene sheets can be synthesized from chemical vapor deposition (CVD) [147]. However, the CVD approach is still an expensive process for manufacturing high-quality graphene sheets. To tackle this, GO can be prepared at a large scale by exposing the graphite flakes in a strong oxidizing solution, i.e., a mixture of sulfuric acid, sodium nitrate, and potassium permanganate, using a modified Hummers process [148]. As a result, GO bears oxygen functional groups having hydroxyl and epoxide on the graphene basal plane, with carboxyl and carbonyl groups at the edges [149]. Those oxygenated groups damage the conjugated structure of graphene, leading to poor electrical conductivity. To resume its electrical conducting properties, reducing agents, such as hydrazine and sodium borohydride, are used to reduce GO to form rGO [150]. The aforementioned reductants are toxic, so green reductants such as L-ascorbic acid, D-glucose and tea polyphenol can be used to reduce GO to rGO [151]. Generally, all chemical reductants cannot remove oxygenated groups of GO completely, rendering rGO with a certain degree of residual oxygen levels. Accordingly, GO would change from insulating to conducting behaviors by regulating the C/O ratios. GO and rGO with a tunable band gap of 4.3–2.4 eV is dependent upon the oxygen level; the bandgap generally increases with increasing O levels [152]. In contrast, pure graphene exhibits no bandgap with excellent electron mobility.

**Figure 6:**
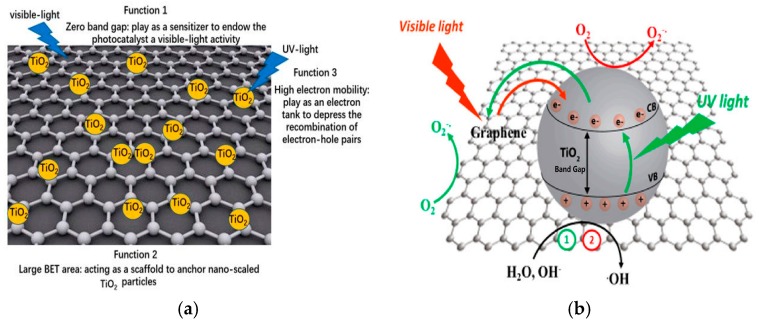
Schematics displaying the (**a**) roles of graphene layers of graphene/TiO_2_ composite in photocatalysis, and (**b**) charge transfer mechanism under ultraviolet or visible light irradiation. Reproduced with permission from [145,153], respectively. Copyright MDPI, 2018 and 2017.

**Figure 7:**
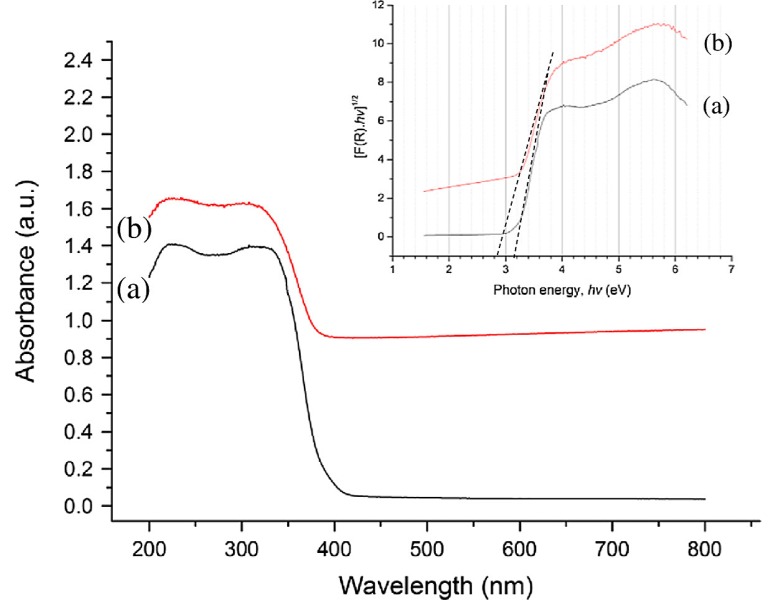
UV–vis diffuse reflectance spectra of (**a**) anatase TiO_2_ and (**b**) nanostructured rGO/TiO_2_. Inset: plot of transformed KM function [F(R).*hv*]^1/2^ vs. *hv* for bandgap determination of anatase TiO_2_ and rGO/TiO_2_; R is reflectance and *hv* is photon energy. Reproduced with permission from [146]. Copyright Springer, 2013.

For novel graphene/TiO_2_ nanostructures, the migration of photogenerated charge carriers from TiO_2_ to graphene or vice-versa depends upon the interfacial contact between them, and the photon energy or wavelength [153]. As is known, uniformly dispersed TiO_2_ NPs on a large-area graphene sheet and a close interfacial interaction between them are essential for efficient charge transport across the interface (Figure 6a). Under UV irradiation, photoexcited electrons from titania are injected into graphene as the conduction band minimum of TiO_2_ is higher than the Fermi level of graphene [32]. As such, highly conductive graphene acts as an electron acceptor for titania, and provides a network to facilitate the rapid transfer of excited electrons. These promote the separation between electron–hole pairs and inhibit their recombination [154]. Under visible light illumination, electrons located in high-energy graphene states are delocalized into the conduction band of TiO_2_. Consequently, electrons react with oxygen adsorbed on the TiO_2_ surface to form superoxide anion (Figure 6b) [153]. In general, a few layer graphene sheets of rGO/TiO_2_ photocatalyst facilitate a red shift in the optical absorption, thereby narrowing its bandgap and enhancing its photocatalytic efficiency [144,146]. Figure 7 shows the UV-vis spectra of anatase TiO_2_ and rGO/TiO_2_ nanocomposite. The inset displays the Tauc plot of the modified Kubelka–Munk (KM) function with a linear extrapolation to produce respective bandgap values of TiO_2_ and rGO/TiO_2_ materials, i.e., 3.2 eV and 2.9 eV.

A single-walled carbon nanotube (SWNT) is formed by rolling-up a graphene sheet into a cylindrical or turbular shape, while several sheets rolls into a multi-walled nanotube (MWNT). The MWNTs with a large surface-area-to-volume ratio and remarkable electrical conductivity serve as the template for anchoring TiO_2_ NPs, facilitating the separation of electron–hole pairs and inhibiting the charge recombination by trapping photoexcited electrons from titania [154]. Accordingly, the bandgap of TiO_2_ NPs reduces from 3.25 to 2.71 eV with an increase in MWNTs content. This leads to a shift in the absorption edge into the visible region. The Ti–O–C bond extends the light absorption to longer wavelengths [155,156,157]. 

### 3.3. Non-Metal Doping

**Figure 8:**
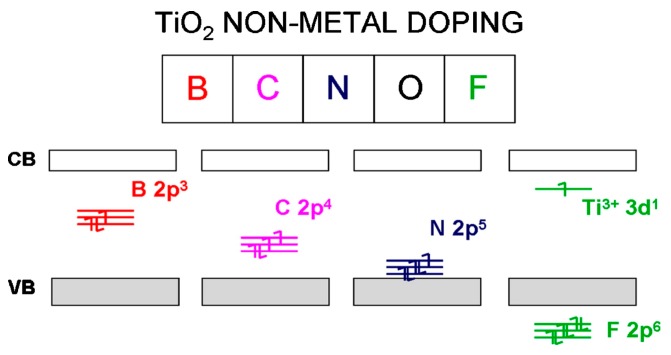
Electronic band structure of titania due to non-metal doping. CB and VB represent conduction band and valence band, respectively. Reproduced with permission from [160]. Copyright Elsevier, 2013.

Metal doping has some drawbacks for enhancing the visible light response of titania. These include transition metals of high contents, which may serve as recombination sites for photogenerated charge carriers, the low thermal stability of photocatalysts, the formation of secondary phases and dopant insolubility [105,158,159]. Therefore, significant improvement in the photocatalytic performance of metal-doped titania can be achieved only at a low metal dopant concentration. Above an optimal dopant content, photocatalytic activity decreases owing to a higher recombination rate of charge carriers. Non-metal elements such as carbon, nitrogen and boron, with an atomic radius close to that of the O atom, can be utilized as anionic dopants for replacing lattice oxygen anions [76,97,159,160,161,162,163,164]. In this respect, non-metal doping appears to be an alternative route for enhancing visible light efficiency, due to the introduction of a new valence band associated with their localized 2p states lying above the valence band of TiO_2_ (Figure 8). As such, non-metal doping generates a hybridization of O-2p and N-2p orbitals, giving rise to an upshift in the valence band position. By irradiating with visible light, electrons are excited from the localized N-2p states to the CB, leaving behind holes on the localized states. The exception is fluorine with the highest electronegativity, having filled states below the O-2p valence band, leading to the formation of Ti^3+^ ions as of result of the charge compensation [165]. 

Among anionic dopants, nitrogen is widely employed to enhance the visible light response of titania. The N atom can occupy either the substitutional or interstitial site of the titania lattice. In the former case, the N atom substitute the O atom to yield TiO_2−x_N_x_, so that the doping energy state (N-2p) lies just above the valence band. Substitutional N doping reduces the bandgap of titania slightly, from 3.20 to ~3.06 eV. The interstitial N-doping reduces the bandgap to ~2.46 eV, in which the doping energy level lies in the midgap, i.e., at 0.74 eV above the valence band [166,167,168]. In an earlier study by Asahi et al., N-doping into substitutional sites of TiO_2_ is reported to be essential for bandgap reduction and efficient photocatalytic activity [166]. For the C-doped TiO_2_ photocatalyst, carbon dopant may replace oxygen or Ti in the substitutional lattice site. It may also occupy the interstitial site [169]. Therefore, C-doped TiO_2_ can have different photocatalytic behaviors, depending on the synthesis process employed. Density functional theory (DFT) calculations predict that substitutional (to oxygen) carbon and oxygen vacancies are formed at low carbon contents and oxygen-poor conditions. Under oxygen-rich conditions, interstitial and substitutional (to Ti) C atoms are favored [170]. Similarly, the B dopant can substitute for either the O or Ti atom, or can occupy the interstitial position. DFT simulations indicate that a B substitution for Ti is unlikely to take place. In contrast, the boron atom tends to either replace an oxygen atom or occupies the interstitial site [171]. From the X-ray photoelectron spectroscopic (XPS) results, Patel et al. reported that B preferentially occupies the interstitial site at low concentrations (up to 1%), while it occupies the substitutional O site as the concentration increases (≥2%) [172]. 

Recently, Sotelo-Vazquez et al. reported that phosphorus (P)-doping can result in the formation of both cationic (P^5+^) and anionic (P^3−^) states of anatase TiO_2_ films on the basis of XPS results. The P^3−^ state of P-doped TiO_2_ exhibited inferior photocatalytic activity compared to undoped TiO_2_ film. Transient absorption spectroscopic results revealed that charge carrier concentrations increased by several orders of magnitude in films containing P^5+^ species [173]. From the XPS measurements, Gopal et al. demonstrated that the P dopant exists in a P^5+^ state which can replace part of Ti^4+^ through the formation of Ti–O–P bonds, i.e., forming P cation-doped TiO_2_ [174]. As a result, the photocatalytic activity of P-doped titania for degrading methylene blue was much enhanced and superior to undoped TiO_2_. Moreover, X-ray diffraction results indicated that P-dopant increases the thermal stability of TiO_2_ NPs, and retards the phase transition from anatase to rutile. 

From the literature, fluorine doping stabilizes anatase TiO_2_ at elevated temperatures up to 1200 °C [75]. The substitution of fluorine for oxygen in TiO_2_ NPs leads to the creation of an oxygen vacancy [175]. Fluorine doping converts Ti^4+^ to Ti^3+^ in TiO_2_ NPs by charge compensation. The presence of Ti^3+^ suppresses the recombination of the electron–hole pairs and enhances the photocatalytic activity accordingly [165]. Co-doping TiO_2_ NPs with F and N is considered to be very effective in tuning the bandgap to further enhance visible-light photocatalytic activity. Multiple charge transfer transitions occur in the Ti^3+^ localized state, oxygen vacancy and N midgap state of the F–N, co-doped TiO_2_ NPs [176]. Table 1 summarizes visible-light active TiO_2_ NPs doped with metals and non-metals.

### 3.4. Coupling of Semiconductors

The poor photocatalytic efficiency of titania under visible light can be overcome through the formation of a heterojunction structure by coupling with other semiconductors with a suitable energy band level. Titania can be coupled with metal oxides (e.g., Cu_2_O, Fe_2_O_3_, WO_3_) and chalcogenides (e.g., CdS, MoS_2_ and WS_2_) to form a heterojunction for the charge separation in enhanced visible light absorption. Those coupled semiconductors acting as sensitizers should be nontoxic, and exhibit visible light photocatalytic activity, with a bandgap smaller than that of titania [177,178,179,180,181,182,183]. In this respect, photoexcited electrons in the CB and holes in the VB of a sensitizer semiconductor can be transferred to the CB and VB of TiO_2_ NPs [180]. Zinc oxide with good antimicrobial property is unsuitable to form visible-light active ZnO/TiO_2_ nanostructures due to its wide bandgap of 3.37 eV. Cadmium sulfide is toxic and carcinogenic, so it is unfavorable to form CdS/TiO_2_ heterojunction for practical applications. Nontoxic molybdenum disulfide (MoS_2_) with a direct band-gap of 1.9 eV can be coupled with titania to form MoS_2_/TiO_2_ nanocomposites, having excellent visible photocatalytic activity [181]. Oxide semiconductors, such as α-Fe_2_O_3_ and Cu_2_O with a respective small bandgap of 2.2 eV and 2.17 eV, can also form composite photocatalysts, with TiO_2_ having good antibacterial properties under visible light [177,179,182,183]. Inexpensive and nontoxic Cu_2_O, with its efficient electron injection to the conduction band of TiO_2_, is particularly suitable for forming heterojunction photocatalysts [182,183]. 

## 4. Synthesis of Titania Nanomaterials

Titania can be fabricated in the form of thin films, powders, or nanocrystals. Physical deposition techniques such as thermal evaporation, reactive sputtering and pulsed laser deposition, chemical gas-phase atomic layer deposition (ALD) process, and wet chemical deposition methods such as dip-coating, spin-coating, spray coating and sol-gel, have been employed by researchers to prepare TiO_2_ thin films [184,185,186,187,188,189]. Those homogeneous films deposited by physical deposition techniques are beneficial for use in dye-sensitized solar cells, microelectomechanical systems and electroluminescent devices [185,189]. In ALD, chemical precursors react sequentially on various substrate surfaces including carbon nanotubes, forming nanometer-sized films of metal oxides (e.g., TiO_2_ and HfO_2_) [190,191,192]. It offers the advantages of nanometer-level control of both thickness and film composition. For bactericidal applications, wet chemical processing is the most convenient, simple and effective synthesis route for preparing TiO_2_ NPs and nanocomposites. Moreover, the solution chemical synthesis process is capable of producing titania nanomaterials in larger quantities in comparison with the physical processing route. Solution processing techniques include the sol-gel, wet impregnation, photoreduction, hydrothermal and solvothermal processing, electrochemical anodization and electrospinning. 

### 4.1. Solution Processing Route

#### 4.1.1. Sol-Gel Method

Titania colloids can be synthesized through the hydrolysis and condensation reaction of titanium alkoxide in the presence of water, and these reactions are catalyzed by an acid [193,194,195,196]. The sol-gel process involves the transformation of metal alkoxide or metal salt into a solid by adding an excess of water to give a metal–oxo linkage (M–O–M). The hydrolysis facilitates the formation of original nuclei TiO_2_, and the subsequent condensation promotes the growth of a crosslinked network of TiO_2_ nuclei. This strategy allows the formation of TiO_2_ NPs with a high level of chemical purity [196,197]. From an earlier study of Padmanabhan et al., the sol-gel process involved the reaction of titanium tetraisopropoxide (TTIP) with trifluoroacetic acid (TFA), followed by hydrolysis, gelation, drying, and finally calcination at high temperatures. The sol was dried at 90 °C to obtain the gel, and then calcined at 500–900 °C to remove organic substances to form nano-TiO_2_ with a high photocatalytic activity [194]. In a recent study, Lusvard et al. employed different precursors and procedures for synthesizing TiO_2_ NPs with the preparation conditions compatible with the industrial scale for water purification [196]. Three different kinds of precursors were utilized for the synthesis of TiO_2_ NPs, including: titanium tetrachloride (TiCl_4_) and ethanol, titanium isopropoxide (C_12_H_28_O_4_Ti) and urea (CO(NH_2_)_2_), as well as titanium isopropoxide, isopropyl alcohol (C_3_H_8_O), acetic acid (CH_3_COOH) and methanol (CH_3_OH). They reported that TiO_2_ NPs, synthesized from molar TTIP: urea in a ratio of 2:1 at 50 °C, have the best photocatalytic activity for degrading methyl blue and bromothymol blue [196]. 

For fabricating metal-doped TiO_2_ nanopowders, an additional metal source reagent is needed, and added to titanium precursors during the sol-gel process [197,198,199,200]. For instance, Marami et al. prepared Fe-doped TiO_2_ powders by introducing FeSO_4_·7H_2_O into the TTIP, and the ethanol solution followed with the addition of acetic acid under vigorous stirring. Thereafter, the temperature of mixture was increased to 70 °C, and ethylene glycol was added, acting as a stabilizer. The product was dried and finally calcined at 600 °C for 4 h to yield Fe-doped TiO_2_ nanopowders [198]. In the case of Ag-doped TiO_2_, a desired amount of silver salt precursor, i.e., silver nitrate was added to the TTIP−methanol solution [50,199]. Reducing agents such as NaBH_4_ are employed to reduce silver ions to AgNPs. For the synthesis of N-doped TiO_2_, an organic compound with nitrogen (such as trimethylamine, 1,3-diaminopropane, ethylmethylamine), or ammonium salt bearing nitrogen (e.g., ammonium carbonate, ammonium chloride, ammonium nitrate), is added to the sol-gel solution during the synthesis process [200,201,202,203,204]. 

#### 4.1.2. Hydrothermal/Solvothermal Synthesis

The hydrothermal/solvothermal method is a useful tool for fabricating TiO_2_ nanostructures involving chemical reactions in a solvent (water/nonaqueous) medium at an elevated temperature >100 °C and a pressure higher than 1 atm, within a closed system using an autoclave. As the sol-gel process generally produces amorphous or low crystalline materials, a subsequent annealing at high temperatures for crystallization is needed. In this context, hydrothermal or solvothermal processing is beneficial for improving the crystallinity of titania synthesized by the sol-gel technique. For example, Yanagizawa and Ovenstone investigated the effect of hydrothermal treatment on the crystallinity and phase structure of sol-gel prepared, TiO_2_ amorphous powders [205]. In their study, hydrothermal treatment was performed at 250 °C for 1 h in the presence of several inorganic salts under acidic and basic conditions. Acidic conditions led to the formation of anatase, brookite, and rutile, whereas basic conditions and/or the presence of sulfate ions favored the crystallization of anatase [205]. In addition, the hydrothermal approach can also be used to synthesize rGO/TiO_2_ nanocomposites [206]. 

**Figure 9:**
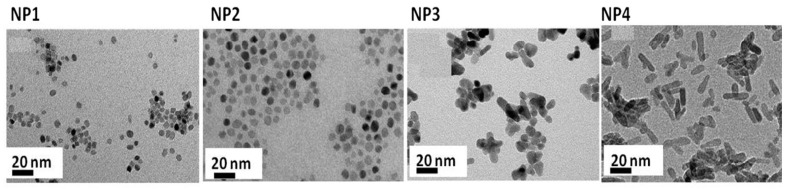
Transmission electron micrographs of solvothermally synthesized TiO_2_ with nanospheres (NP1–NP2) and nanorods (NP3–NP4) morphologies. Reproduced with permission from [209]. Copyright Elsevier, 2017.

The organic solvents in solvothermal treatment help to control the morphology of synthesized nanocrystals. Thus, this process enables better control of the shape, size distribution and crystallinity of TiO_2_ NPs in comparison with the hydrothermal method. TiO_2_ nanostructures of different morphologies can be obtained and tailored by manipulating several processing parameters, including the type of solvent and titanium precursor, molar ratio of reagents, addition of surfactant, reaction temperature and time [207,208,209,210,211,212]. For instance, TiO_2_ nanorods can be synthesized in TTIP, benzyl alcohol (BzOH) and acetic acid (AA) at 150 °C for 8 h. The molar ratio of TTIP/AA is kept at 1: 4 [208]. Recently, Falentin-Daudré et al. synthesized highly crystalline sphere and rod-shaped TiO_2_ nanostructures using TTIP, benzyl alcohol (BzOH) and AA reagents. The shape of the TiO_2_ nanostructure can be tuned by varying the concentration molar ratios of TTIP/BzOH and AA/BzOH (Figure 9) [209]. The X-ray diffraction patterns for TiO_2_ nanospheres and nanorods display well-defined peaks associated with pure anatase, thus revealing TiO_2_ nanospheres and nanorods with a high crystallinity.

**Figure 10:**
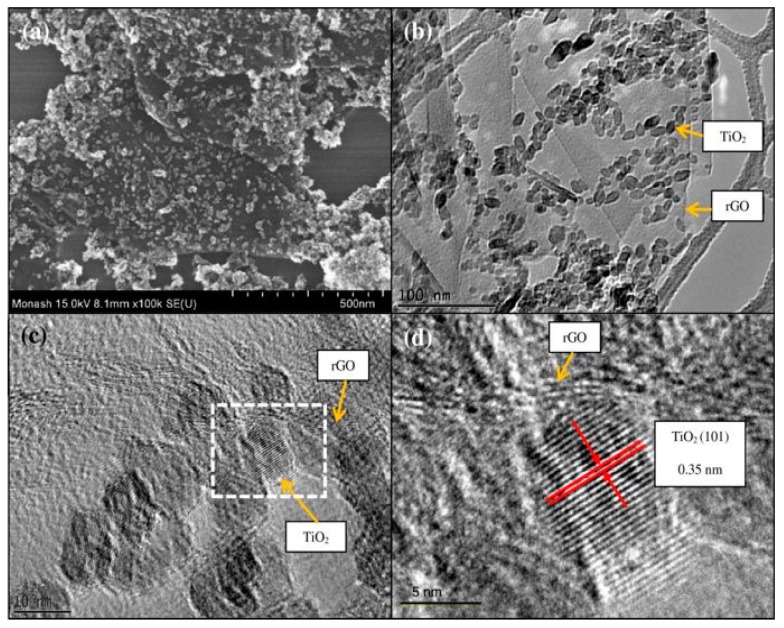
(**a**) Field-emission scanning electron image and (**b**) transmission electron micrograph of the solvothermally synthesized rGO/TiO_2_ nanocomposite. (**c**,**d**) Enlarged images of high-resolution transmission electron micrographs showing the lattice fringes of TiO_2_ and a clean interface between TiO_2_ and rGO. Reproduced with permission from [146]. Copyright Springer, 2013.

For preparing rGO/TiO_2_ nanocomposite, Tan et al. first obtained a mixed solution of tetrabutyl titanate, ethylene glycol and acetic acid, and then added it dropwise into a chilled GO solution under vigorous stirring. Thereafter, an autoclave filled with the GO–TiO_2_ solution was heated at 180 °C for 8 h. The greyish-black precipitate was obtained by centrifugation [146]. During the solvothermal synthesis, GO was reduced to rGO accordingly. Figure 10a,b show the respective field-emission scanning electron microscopic (FESEM) image and transmission electron micrograph (TEM) of the rGO/TiO_2_ nanocomposite. It is apparent that titania nanoparticles with an average size of 12 nm are dispersed and anchored on the rGO surface. The high-resolution TEM (HRTEM) images of a selected rGO-TiO_2_ heterojunction are shown in Figure 10c,d. The lattice fringes can be seen in titania nanoparticles, especially in the high magnification image shown in Figure 10d, implying that the TiO_2_ nanocrystals exhibit good crystallinity. The lattice spacing of TiO_2_ is determined to be 0.35 nm, which corresponds to the (101) plane of anatase TiO_2_. Moreover, the rGO–TiO_2_ interface is clean and free from the impurity products. The intimate connection enables photoinduced electrons to flow readily from rGO to TiO_2_ NPs, thereby enhancing the photocatalytic activity. 

#### 4.1.3. Electrochemical Anodization

Transition metals like iron and chromium can form a thin oxide film on their surface upon electrochemical polarization in the anodic region [213,214]. Therefore, titanium and its alloys can also form anodic films on their surfaces during the anodizing process. Ti-based alloys are widely used as load-bearing bone prostheses and dental implants in clinical sectors. However, bacterial infection due to biofilm formation is the main cause of implant failures. In recent years, there has been a clinical demand for functional Ti-prostheses with enhanced bone cell adhesion/growth, and excellent antibacterial properties. To improve the biocompatibility of Ti-implants with the host-tissues, titania coating is formed on their surfaces through the anodization technique [215]. One-dimensional titania nanotubes’ (TNTs) high surface area to volume ratio and enhanced bone–cell adhesion ability makes them suitable for biomedical applications [216,217,218,219]. TNTs promote the osseointegration of bone implants more effectively than titanium alloys. Compared with TiO_2_ NPs, TNTs bear a stronger negative surface charge [55], enabling them to repel bacteria with a negatively charged membrane. Thus, TNTs show bactericidal effects to a lesser degree. By incorporating AgNPs into TNTs, the bactericidal performance of anodized Ti-alloys is improved significantly [218]. The TNTs fabricated from the sol-gel or hydrothermal methods are randomly oriented [220]. In contrast, ordered and self-organized titania nanotubes can be prepared by electrochemical anodization [219]. Anodization offers the additional advantages of simplicity, and ease of fabrication and scaling-up. The tube diameter, length and wall thickness can be properly manipulated by processing parameters including electrolyte composition, applied voltage, pH, temperature, and time [221]. In general, the applied voltage regulates the nanotube diameter, and the anodizing time controls the tube length. 

**Figure 11:**
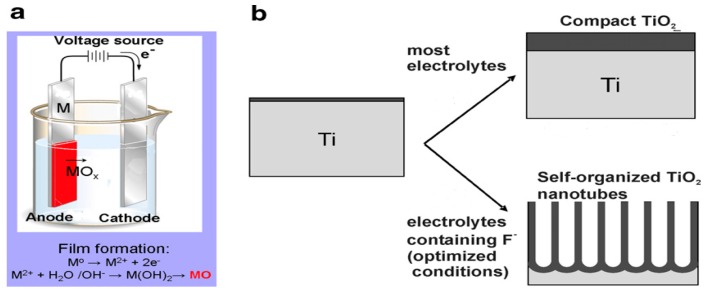
Schematic illustration displaying (**a**) the set-up for anodization and (**b**) frmation of compact titania layer on the Ti substrate in electrolytes without fluoride, and self-organized titania nanotube arrays in electrolytes with fluoride. Reproduced with permission from [222]. Copyright Elsevier, 2007.

Titanium anodization can be simply performed in a two-electrode cell system connected to a power supply (Figure 11a). The oxide film formation involves an anodic oxidation of metal at the metal surface, outward migration of Ti^4+^ ions toward the metal/oxide interface and field-assisted dissolution of oxide at the oxide/electrolyte interface [219]. The oxide layer generally has a low conductivity, which restricts the migration of oxygen and Ti ions accordingly. As such, continued oxide growth is assisted by an electric field, and a compact oxide layer is formed on the Ti surface. The electrochemical reactions occurring during anodization are given as follows

Ti + 2H_2_O + 4e→ TiO_2_ + 4H^+^ Anodic oxidation, (6)

4H^+^ + 4e → 2H_2_ Cathodic reaction. (7)

To form TNTs on Ti foil substrate, aqueous fluoride-containing electrolytes such as (NH_4_)_2_HPO_4_/NH_4_F or (NH_4_)_2_SO_4_/NH_4_F, and organic electrolytes, e.g., ethylene glycol, formamide, or dimethylsulfoxide containing F^−^ anions, are needed (Figure 11b). The presence of F^−^ anions in the electrolyte results in the chemical dissolution of oxide at the electrolyte/oxide interface to yield [TiF_6_]^2−^ and F^−-^rich layers. In other words, F^−^ ions etch the oxide layer to form water-soluble [TiF_6_]^2−^ complexes. The chemical reaction associated with the F^−^ ions etching is given by [222]

TiO_2_ + 6F^−^ + 4H^+^ → [TiF_6_]^2−^ +2H_2_O (8)

**Figure 12:**
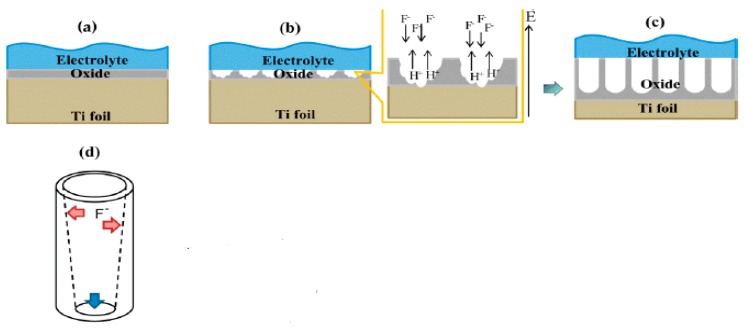
Schematic showing the formation of titania nanotube (TNT) arrays: (**a**) an initial development of a compact oxide layer on the surface of Ti, (**b**) small pits formation due to the etching of oxide by F^−^ ions, (**c**) local growth of nanopores into well-aligned TNT arrays, and (**d**) the shape and wall thickness of a nanotube. Reproduced with permission from [223]. Copyright MDPI, 2019.

**Figure 13:**
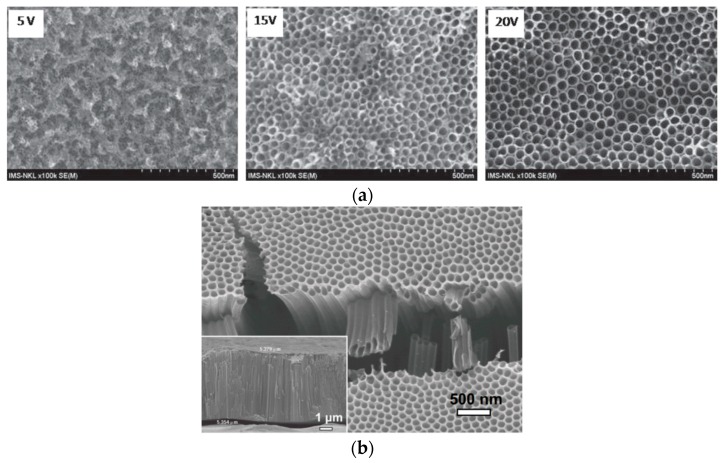
(**a**) Top-view scanning electron micrographs of TNT arrays prepared by anodizing Ti in a mixed ethylene glycol/NH_4_F and water solution under applied voltages of 5, 15 and 20 V for 5 h. Reproduced with permission from [225]. Copyright IOP Publishing, 2014. (**b**) Cross-sectional SEM image (inset) and top view of TNTs fabricated by anodizing Ti in a mixed ethylene glycol/NH_4_F and water solution at 30 V for 1 h. Reproduced with permission from [223]. Copyright MDPI, 2019.

Accordingly, small pits are produced at the electrolyte/oxide interface due to the chemical dissolution of oxide. These pits gradually grow into nanopores, as shown in Figure 12a,b. The pores grow into tubular features and form TNT arrays as the anodizing process continues to its final stage (Figure 12c,d). The growth of TNT arrays is described as the competition between electrochemical oxide formation and chemical dissolution of oxide by F^−^ ions of sufficient concentrations [222,223,224]. Figure 13a,b shows the formation of TNT arrays by anodizing Ti in a mixed ethylene glycol/NH_4_F and water solution [223,225]. At a low applied voltage of 5 V, an SEM image shows the formation of the rough Ti surface together with inhomogeneous TNTs. However, uniform and well-aligned TNTs are produced by increasing the applied voltage from 15 to 20 V (Figure 13a). The as-anodized TiO_2_ nanotubes generally exhibit an amorphous structure. Therefore, post-annealing treatment is typically performed to enhance their crystallinity.

**Figure 14:**
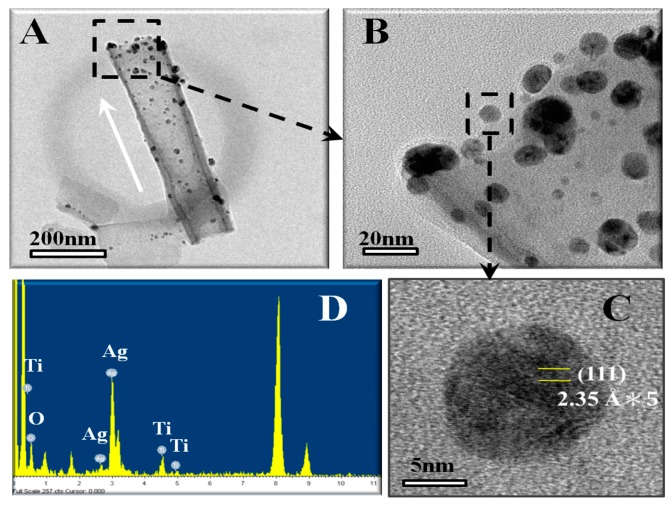
(**A**) TEM image of a single, Ag-decorated TiO_2_ nanotube with a diameter of 100 nm. The white arrow indicates the growth direction of a nanotube. (**B**) High-magnification image of the selected are, as marked by a dashed square in (**A**). (**C**) Enlarged view of a single AgNP, and (**D**) the corresponding EDS spectrum of AgNP and TiO_2_ nanotubes. Reproduced with permission from [226]. Copyright Public Library of Science, 2013.

To introduce AgNPs into TNTs, Lan et al. deposited a thin Ag layer on anodized TNTs via electron-beam evaporation. AgNPs were directly coated onto inner- and outer-tube surfaces [226]. Figure 14A is the TEM image of AgNP-decorated TNTs, showing the uniform distribution of AgNPs along the tubes. High-magnification TEM images reveal that the sizes of AgNPs range from 5 to 20 nm (Figure 14B,C). The corresponding energy dispersive X-ray spectrum (EDS) reveals the presence of Ag in addition to Ti from the TNT (Figure 14D). Alternatively, the wet chemical synthesis route using TNT and silver nitrate mixed solution can yield Ag-decorated TNTs. Thereafter, UV illumination is employed to reduce Ag^+^ ions to AgNPs through the photoreduction process without using reducing agents. As such, photo-assisted deposition can bind AgNPs closely to TNTs [53]. 

#### 4.1.4. Electrospinning

Electrospinning is a simple and versatile tool to form microfibers and nanofibers (NFs) from different materials including polymers, metal oxides and their nanocomposites. This process has been used extensively for fabricating nanofibers derived from polymers and polymer nanocomposites [27,72,227,228]. In the process, a high electric field is applied to the polymer/solvent solution. Beyond a critical voltage, the repulsive electrostatic force overcomes the surface tension of the polymer droplet, resulting in the ejection of a charged jet from the nozzle towards the collector. Several processing parameters affect the fiber diameter and porosity of electrospun polymer mats, including the type of solvent used, polymer concentration, applied voltage, flow rate, needle-to-collector distance, etc. [227,228]. Very recently, Feng et al. incorporated commercial Degussa P25 (70–80% anatase and 20–30% rutile) with a diameter of 20 nm into polylactic acid (PLA) using the electrospinning method [229]. They reported that the PLA/TiO_2_ composite nanofibers with 0.75 wt% TiO_2_ exhibit good bactericidal activity upon exposure to UV-A (360 nm) radiation. 

In general, two approaches have been employed to prepare electrospun metal oxide NFs, i.e., a polymer-assisted spinning method and direct electrospinning without using a polymer [230,231,232,233,234]. The former strategy involves the mixing of metal alkoxide sol with a polymer solution in which the polymer controls the rheology during electrospinning. Without a polymer solution, the viscosity of the sol varies with time, causing a difficulty in controlling the rheological properties of a sol. In addition, the diameter of the as-spun ceramic fibers falls in the micrometer scale [232]. To improve solution spinnability, poly (vinyl pyrrolidone) (PVP) is added to the sol to obtain continuous ceramic NFs. As such, the diameter of titania fibers can be tuned from the micrometer to nanometer scale by regulating the concentration of PVP and the Ti alkoxide to PVP ratio. For example, Tekmen et al. electrospun TiO_2_ with a diameter of 54–78 nm, employing a mixture solution of PVP and TTIP [231]. 

**Figure 15:**
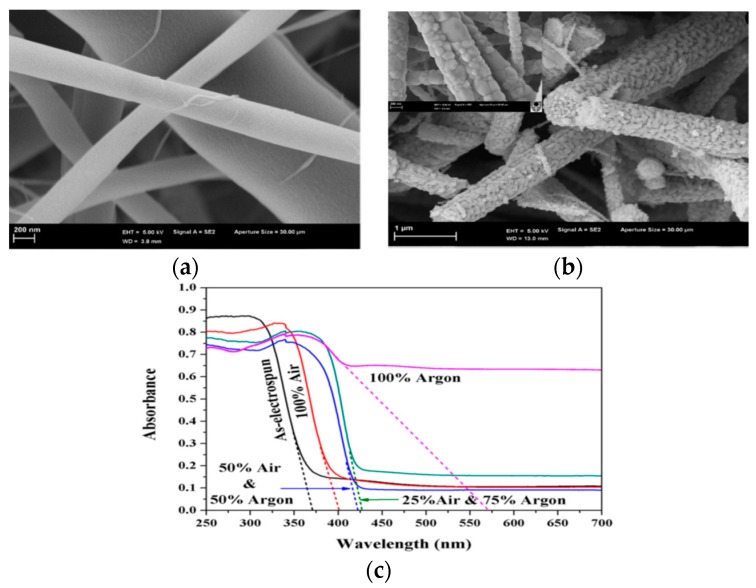
Scanning electron micrographs of (**a**) electrospun titania nanofibers before heating, and (**b**) after heating in 100% argon atmosphere at 900 °C. (**c**) UV-visible spectra of as-spun titania nanofibers without calcination, and with calcination at 900 °C in 100% air, 50% air–50% argon, 25% air–75% argon and 100% argon. Reproduced with permission from [233]. Copyright Elsevier, 2016.

Albetran et al. studied the effect of calcination treatment on the bandgap reduction in electrospun titania nanofibers exposed to pure argon, air, and air–argon mixtures at 900 °C [233]. The spinning solution was prepared by mixing TTIP, ethanol, and acetic acid in a fixed volume ratio of 3:3:1, followed by the addition of 12 wt% PVP. The nanofibers heated in 100% argon exhibit an uneven or rough surface in comparison with the as-spun amorphous fibers due to the formation of crystalline grains of anatase and rutile (Figure 15a,b). In general, the anatase phase is stable in TiO_2_ up to 500–700 °C, and transforms to rutile with an increase in temperature [75]. Calcination at 900 °C led to a reduction in the diameter of NFs due to the removal of PVP and the densification of TiO_2_. The degree of crystallinity of calcined titania NFs was 73.4%. Moreover, calcination of the as-spun NFs in 100% argon induced the formation of a high amount of oxygen vacancies, thereby creating a localized state below the conduction band, and reducing the bandgap accordingly. The creation of oxygen vacancies was reported to be effective to enhance visible light absorption as the oxygen vacancy states were located 0.75 to 1.18 eV below the conduction band minimum of TiO_2_. Those oxygen vacancies were generated in titania by heating in an oxygen-poor environment, such as a N_2_, Ar or a vacuum at elevated temperatures (>400 °C) [235]. From Figure 15c, the as-spun mat calcined at 900 °C in a 100% argon atmosphere had the highest absorbance in the visible light region compared with the as-spun mats with and without calcination in air and air–argon gaseous mixtures. The bandgap of as-electrospun amorphous nanofibers determined from the UV-vis spectra reduced from 3.33 to 3.09, 2.91 and 2.18 eV through calcination in air, 25% air/75% argon and 100% argon, respectively. Nasr et al. electrospun (2%–7 wt%) rGO/TiO_2_ NFs, followed by annealing at 500 °C [236]. The rGO sheets reduced the bandgap of TiO_2_ NFs from 3.2 to 2.9 eV, thus suppressing the recombination of electron–hole pairs, and increasing visible-light photocatalytic activity for degrading methylene blue. Very recently, Chapman et al. successfully obtained TiO_2_ NFs with average diameters of ~70 nm without using a polymer through mixing an alkoxide precursor, solvent, water, and an acid [234]. They introduced TTIP in ethanol, aged under nitric acid condition, and then added *N,N*-dimethylformamide to obtain a sol needed for the continuous spinning of TiO_2_ NFs.

## 5. Bactericidal Activities

Titania NPs are negatively charged at the point of zero charge (pzc) at pH = 6.2. Therefore, they exhibit low bactericidal activity in neutral and alkaline solutions by repelling negatively charged bacteria in the absence of light. At acidic pH, positively charged TiO_2_ NPs strongly interact with the bacterial cells, resulting in bacterial membrane penetration and inducing oxidative damage accordingly [57]. Kiwi et al. reported that TiO_2_ NPs tend to kill *E. coli* by direct contact in the dark condition, thus damaging their cell walls, due to the electrostatic attraction between the TiO_2_ NPs and the negatively charged bacterial cell wall at a pH close to but below pzc [237]. On the contrary, the bactericidal effect is caused by the creation of ROS species on the TiO_2_ NPs under UV irradiation. This means that the bactericidal activity is due to the radiation itself and is not caused by the titania NPs [238]. TiO_2_ NPs can kill multidrug-resistant bacteria such as MRSA, vancomycin-resistant Enterococcus faecalis (VRE) and *P. aeruginosa* through the reactive radicals generated by electron–hole pairs upon UV excitation [239,240]. The photocatalytic inactivation of MRSA and VRE strains depends on the power and irradiation time of UV-A light [239]. Therefore, the disinfection process requires a high-power UV source to excite TiO_2_ NPs. Apparently, TiO_2_ NPs do not reach their full potential for bactericidal applications owing to their ineffective photoexcitation under visible light irradiation. As a result, TiO_2_ NPs have limited efficiency against microorganisms in indoor environments where the fraction of UV light is small. From this perspective, the development of a visible-light active TiO_2_ with excellent antibacterial performance is of crucial importance in medical and industrial sectors.

### 5.1. Metal Doping

Transition metals like Cr, Fe, Ni, Cu, and RE metals can be used to enhance the photocatalytic activity of nanocrystalline titania, and this in turn improves its bactericidal performance. Those metal cations substitute Ti^4+^ ions in the titania lattice, leading to a reduction in the bandgap and promoting the formation of charge carriers under visible light. As a result, ROS are generated on the titania surface, and they are very effective at killing bacteria through lipid peroxidation, the depletion of glutathione, DNA damage and the final disintegration of the cell membrane. This results in a leakage of cellular contents, thus causing cell lysis and eventual cell death [241,242]. Negatively charged superoxide and hydroxyl radicals generally reside on the membrane and do not penetrate into the bacterial cytoplasm, while electrically neutral H_2_O_2_ can pass through the cell membrane. Hydrogen radicals can abstract hydrogen atoms from the fatty acids of bacterial membrane lipids, causing lipid peroxidation and damaging the respiratory electron transport chain located in the membrane [242]. As is known, most transition and RE metals are toxic to humans. In terms of environmental and public health considerations, Cu is more suitable than other transition metals and RE metals for doping nanocrystalline titania. Copper metal is widely used in hospitals for preventing spread of bacteria among the patients because of its antimicrobial activity [243]. Therefore, copper can be used to dope titania for antibacterial purposes [244,245,246,247]. As an example, TiO_2_–Cu films exhibit bacterial inactivation for *E. coli* and MRSA under indoor visible light irradiation [244,245]. 

#### 5.1.1. Doped Titania NPs

**Figure 16:**
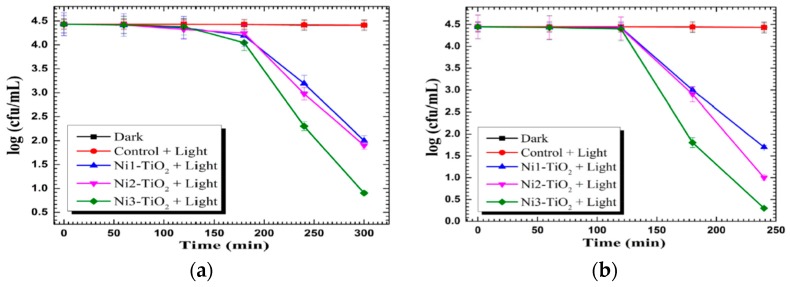
Inactivation of (**a**) *E. coli* and (**b**) *S. aureus* by Ni-doped TiO_2_ NPs as a function of time. Reproduced with permission from [248]. Copyright Elsevier, 2014.

Yadav et al. fabricated Ni-doped TiO_2_ NPs using the sol-gel process through the addition of NiSO_4_(H_2_O)_6_ to a mixture solution containing TTIP, acetic acid and sodium dodecyl sulfate. The resulting powders were dried and calcined at 500 °C for 5 h [248]. Figure 16a,b shows the photocatalytic bactericidal activity against *E. coli* and *S. aureus* of Ni-doped TiO_2_ NPs with 1.0 mol %, 2.0 mol % and 3.0 mol % Ni dopants, denoting Ni1–TiO_2_, Ni2–TiO_2_ and Ni3–TiO_2_, respectively. In dark (with doped TiO_2_ NPs) and visible light (without doped TiO_2_ NPs) environments, both bacterial strains grow into a high density of cell populations, expressed as colony-forming units (CFU)/mL. The photocatalytic inactivation of *E. coli* and *S. aureus* takes place by illuminating Ni-doped TiO_2_ NPs with visible light. The Ni3–TiO_2_ sample shows the highest photocatalytic inactivation because it can generate a higher ROS level with an increase in Ni content. Moreover, the photocatalytic inactivation efficiency of Ni-doped TiO_2_ NPs toward Gram-positive *S. aureus* is somewhat faster than Gram negative *E. coli*. In the case of Gram-negative salmonella abony, complete inactivation takes 360 min of light irradiation (data not shown). The time required for the full inactivation of S. abony is higher than that for *E. coli* inactivation. Using the same approach, they also fabricated Cu-doped TiO_2_ NPs with 1.0 mol %, 2.0 mol % and 3.0 mol % Cu by adding different concentrations of CuSO_4_·5H_2_O to a solution containing TTIP and acetic acid. The catalysts were calcined in air at 500 °C for 5 h [247]. The 3%Cu/TiO_2_ NPs photocatalyst exhibits a higher bactericidal activity than those doped with 1.0 mol %, and 2.0 mol % Cu. The 3%Cu/TiO_2_ NPs catalyst shows 100% inhibition for *S. aureus* within 120 min, but it requires 240 min for the complete inactivation of *E. coli*. This implies that the rate of bacterial inactivation for *E. coli* is much slower than for *S. aureus*. This is caused by a difference in the cell wall structures between these two bacterial strains. As is known, bacteria exhibit a negative charge on their cell wall surface. The cell wall of Gram-positive bacteria is relatively porous and thick (20–80 nm) consisting of several layers of peptidoglycan, interspersed with teichoic and lipoteichoic acids. Peptidoglycan is negatively charged due to the presence of carboxyl and amino groups [249]. In contrast, the cell wall of Gram-negative bacteria is thinner (<10 nm) with a single peptidoglycan layer, surrounded by an outer membrane with a very complex structure. Lipopolysaccharides (LPS) and lipoproteins are located in the outer leaflet, while phospholipids are found in the inner leaflet of the outer membrane. The phosphate groups of LPS increase the overall negative charge. Thus Gram-negative bacteria have a higher negative charge than Gram-positive bacteria [250,251,252]. The structural variations in the cell walls between these two bacterial strains lead to their different interactions with photocatalysts. As such, Gram-negative bacteria is more resistant to attack from the superoxide anion and hydroxy radical with a negative charge. Moreover, LPS also creates a permeability barrier at the cell surface, thus contributing to its resistance against many antibiotics and substances [250,251,252]. 

**Figure 17:**
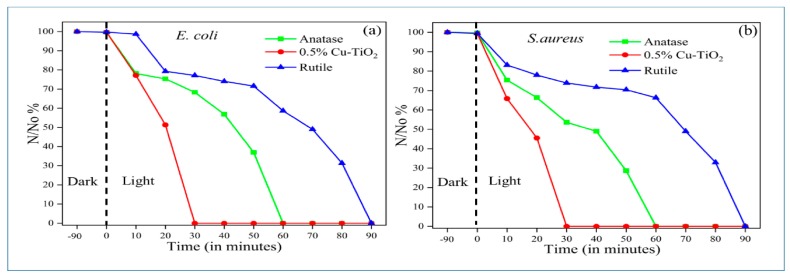
Photocatalytic inactivation of (**a**) *E. coli* and (**b**) *S. aureus* with 0.5% Cu/TiO_2_ calcined at 650 °C, pure anatase and rutile specimens. N/N_o_ is the reduction in the concentration of the bacteria. Reproduced with permission from [109]. Copyright MDPI, 2018.

**Figure 18:**
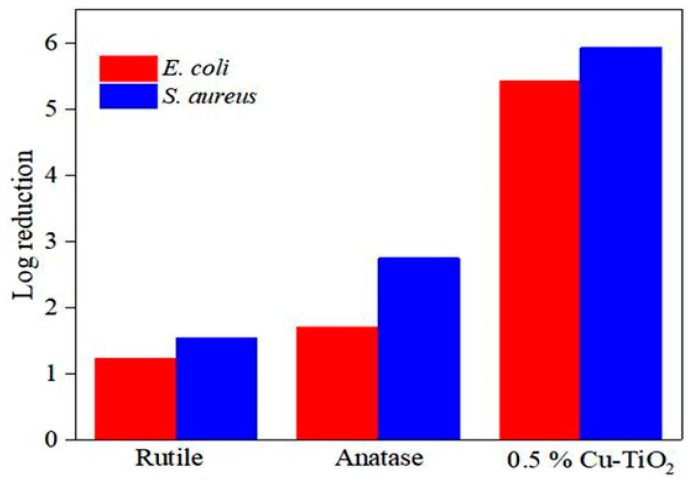
Photocatalytic bactericidal efficacy of 0.5% Cu/TiO_2_, pure anatase and rutile with *E. coli* and *S. aureus* upon visible light irradiation for 30 min. Reproduced with permission from [109]. Copyright MDPI, 2018.

Very recently, Pillai and coworkers prepared Cu-doped TiO_2_ NPs by adding a copper sulfate solution to a mixture solution containing TTIP, and isopropanol [109]. The resulting gel was dried, and doped titania powders were calcined at 500, 600, 650 and 700 °C, respectively. Pure TiO_2_ powders were prepared from TTIP and isopropanol without copper sulphate addition. The obtained TiO_2_ powders were calcined at 500 and 700 °C to yield anatase and rutile, respectively. Their results showed that Cu doping is very effective to retain the anatase phase of TiO_2_ at calcined temperatures up to 650 °C. X-ray Photo-electron Spectroscopy (XPS) spectra reveals the presence of Cu^+^ and Cu^2+^ in Cu-doped TiO_2_ where the Cu^+^ state predominates. Figure 17a,b shows the photocatalytic bactericidal activity of 0.5 wt% Cu/TiO_2_, anatase TiO_2_ and rutile TiO_2_ against *E. coli* and *S. aureus* under dark and visible light illumination, respectively. In the dark, both bacteria strains grow quickly and their survival rate reaches a high plateau value. However, a 5-Log pathogen reduction (99.999%) is observed in both bacterial strains exposed to 0.5 wt% Cu/TiO_2_ following visible light irradiation for 30 min (Figure 18). In this respect, 0.5 wt% Cu/TiO_2_ photocatalyst exhibits a strong antibacterial effect against *E. coli* and S. aureous under visible light irradiation. The enhanced antibacterial performance of 0.5 wt% Cu/TiO_2_ photocatalyst calcined at 650 °C is attributed to the formation of a heterojunction between TiO_2_ and Cu_2_O, inducing hydroxyl radicals through the interfacial charge carrier transfer mechanism, to the copper ions killing effect. The replacement of Ti^4+^ with Cu^2+^ also induces the creation of oxygen vacancies. This gives rise to the high absorption rate of visible light as a result of a bandgap reduction from 3.17 to 2.8 eV. It is noted that some Cu^+^ ions may react with a transient metabolic byproduct of cellular respiration, i.e., H_2_O_2_ through the Fenton reaction, resulting in the formation of hydroxyl radicals and Cu^2+^ ions. The Fenton reaction for generating hydroxyl radicals due to the presence of Cu^+^ ions is given by [246]

Cu^+^ + H_2_O_2_ → Cu^2+^ + OH^−^ + ^•^OH. (9)

Silver nanoparticles with an additional function as a bactericidal agent can be used to modify titania photocatalyst to further enhance its antibacterial performance. From the literature, AgNPs exhibit excellent antibacterial activity against various microorganisms, including *S. aureus*, MRSA, Bacillus subtilis, *E. coli*, *Pseudomonas aeruginosa*, *Klebsiella pneumonia*, and *Acinetobacter baumanii* [253]. Whether metallic Ag^0^ or ionic Ag^+^ released from AgNPs exerts killing effects on bacteria is still unknown [15,253,254,255]. The former mechanism involves the adhesion of AgNPs to the cell membrane, leading to membrane damage, the generation of oxidative stress and leakage of cellular contents. Moreover, AgNPs can move into the cytoplasm and interact with biomolecules such as protein and DNA. In some cases, they inactivate and destabilize ribosome, thus inhibiting protein synthesis and generating ROS accordingly. In the case of silver-ion induced toxic effects, released silver ions would interact with the thiol groups of respiratory chain proteins on the membrane, resulting in the disruption of the bacterial cell wall and the creation of ROS. The electron transport chain for bacterial respiration is located at the bacterial cytoplasmic membrane, since bacteria have no mitochondria (Figure 1). Silver ions can also penetrate into the cytoplasm and react with the thiol groups of cytoplasmic proteins [15,253,254,255]. 

**Figure 19:**
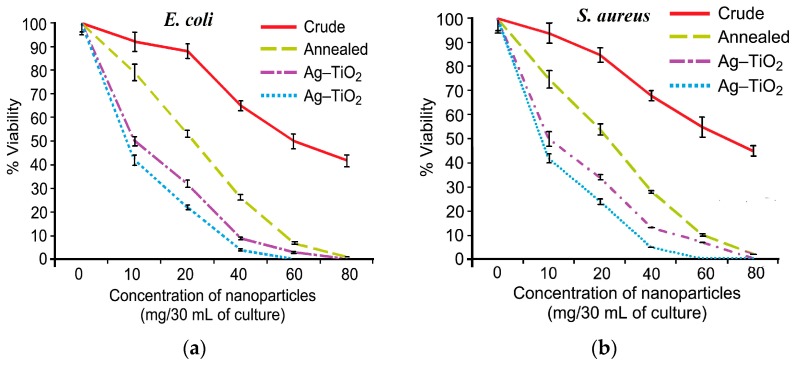
Viability of (**a**) *E. coli* and (**b**) *S. aureus* against the concentration of as-synthesized TiO_2_ NPs, annealed TiO_2_ NPs, and Ag-doped TiO_2_ NPs with 3% AgNPs (dash–dot curve) and 7% AgNPs (dot curve; blue). Reproduced with permission from [256]. Copyright Beilstein-Institut, 2013.

As mentioned, AgNPs can induce a collective oscillation of surface electrons under visible light irradiation, thereby creating a hot electron–hole pair and inducing ROS for bacterial inactivation. Thus, AgNPs serve as electron donors for titania, since plasmonic hot electrons are injected into the conduction band of TiO_2_ and trigger a photocatalytic disinfection reaction to generate superoxide anion, as shown in Figure 6. Accordingly, AgNPs play the dual role of antibacterial agent and electron donor for Ag-doped TiO_2_ NPs [124]. Gupta et al. fabricated Ag-doped TiO_2_ NPs with 3% and 7% AgNPs using the sol-gel process. The resulting powders were dried in an oven followed by annealing at 450 °C for 30 min [256]. The photocatalytic activities of the as-synthesized TiO_2_, annealed TiO_2_, and annealed Ag-doped TiO_2_ materials were assessed against Gram negative *E. coli*, Pseudomonas aeruginosa and Gram positive *S. aureus* under visible light. Figure 19a,b shows the viability of *E. coli* and *S. aureus* versus the concentration of catalyst nanoparticles, respectively. The as-synthesized TiO_2_ NPs with an amorphous structure inactivates some *E. coli* and *S. aureus* because their negatively charged surface can repel bacteria, resulting in a net negative charge on the cell wall [57]. Annealing treatment at 450 °C induces the crystallization of the anatase phase in TiO_2_ NPs. The bactericidal performance of annealed Ag-doped TiO_2_ NPs is markedly improved in comparison with the as-synthesized and annealed TiO_2_ NPs. The Ag-doped TiO_2_ NPs with 7% AgNPs exhibits toxicity to both bacterial strains at 60 mg/30 mL, and at 40 mg/30 mL culture in the case of *P. aeruginosa*. 

#### 5.1.2. Doped Titania Nanotubes

The photocatalytic activity of one-dimensional TNTs is considerably higher than that of TiO_2_ NPs because of their large surface area, high aspect ratio, and good light-harvesting properties [257,258]. Recently, Podporska-Carroll et al. reported that TNTs exhibit very high bactericidal efficiency against *E. coli* (97.53%) and *S. aureus* (99.94%) under 24 h of UV irradiation [259]. Moreover, anodic TNTs exhibit a higher photocatalytic inactivation of bacteria than commercial Degussa P25 TiO_2_ powders. To extend the optical absorbance to the visible-light region and improve bactericial performance in this optical regime, noble metal dopants are added to TNTs accordingly. For instance, Viet et al. demonstrated that the 2 wt% Ag/TNTs photocatalyst exhibits higher bactericidal activity against *S. aureus* than pristine TNTs when exposed to sunlight at noontime [53]. 

**Figure 20:**
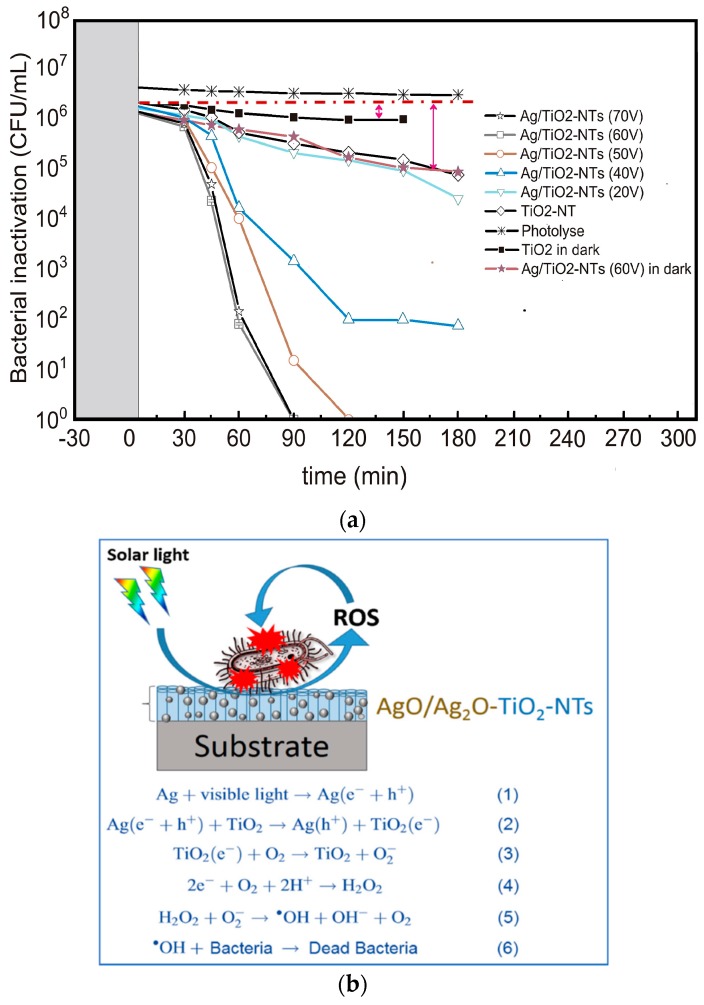
(**a**) Bacterial inactivation on neat TiO_2_–NT and Ag/TNTs photocatalysts exposed to solar-simulated light (50 mW/cm^2^, 310–800 nm). Error bars: standard deviation; *n* = 5. (**b**) Bacterial inactivation mechanism of Ag/TNTs, as described by Reaction (1–6). Reproduced with permission from [260]. Copyright Elsevier, 2018.

Rtimi and coworkers prepared TNTs of different diameters by varying applied voltages from 20 V–70 V during anodizing process. The anodized TNTs were air-dried, annealed for 3 h at 400 °C, and then immersed in a 0.1 M AgNO_3_; Ag^+^ ions were reduced to AgNPs on TNTs using the photoreduction method [260]. A low voltage of 20 V was not favorable for the formation of TNTs. The average diameters of TNTs under applied voltages of 40, 50, 60 and 70 V were 59.6, 93.6, 96.6 and 100. 9 nm, respectively. The tube diameter increased with increasing applied voltage. Figure 20a shows the bacterial survival rate of *E. coli* on pristine TNTs and Ag-decorated TNTs of different diameters upon exposure to solar-simulated light (50 mW/cm^2^). The used light intensity corresponds with the overcast daylight dose. Pristine TiO_2_–NTs inactivate 1.6log E coli within 180 min. Negatively charged TNTs tend to repel *E. coli* with a negative surface charge, giving rise to a low level of antibacterial activity. From this figure, a stronger *E. coli* inactivation can be achieved by increasing the diameter of TNTs. The Ag/TNTs with diameters of 96.6 nm and 100.9 nm exhibit excellent bacterial inactivation compared with neat TNTs. These two samples require 90 min for inactivating 99.99% *E. coli* upon exposure to solar-simulated light. The bacterial inactivation is attributed to the generation of ROS as a result of the plasmonic oscillation of surface electrons of AgNPs caused by solar-simulated light irradiation. This, in turn, leads to the generation of an electron–hole pair in TNTs to create ROS (Figure 20b). Free radicals abstract electrons from the lipid molecules of bacterial membrane, leading accordingly to lipid peroxidation and membrane damage.

**Figure 21:**
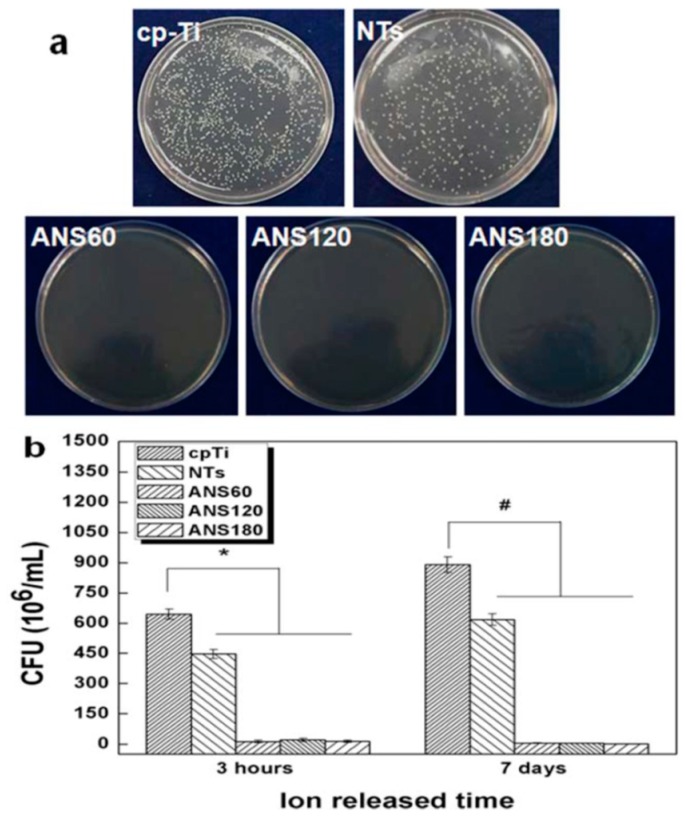
(a) Photographs showing the spread of *S. aureus* on commercially pure titanium (cpTi), titanium nanotubes (NTs) and Ag-doped TNTs samples. (**b**) Antibacterial efficacy of all samples immersed in PBS for 3 h and 7 d. The error bars are the standard deviation (*n* = 5); * denotes *p* < 0.05 compared with cp-Ti at a 3 h ion extraction time, # denotes *p* < 0.05 compared with cp-Ti at 7 days ion extraction time. Reproduced with permission from [261]. Copyright Wiley, 2014.

The AgNPs of silver-decorated TNTs also play the role of antibacterial agent through the released Ag^+^ ions. Uhm et al. fabricated Ag-doped TNTs by depositing a thin silver layer onto anodized TNTs via magnetron sputtering for different time periods [261]. The TNTs samples coated with silver for 60, 120 and 180 s were designated as ANS 60, ANS 120 and ANS 180, respectively. A longer sputtering time induced more AgNPs formation on the nanotubes, as expected. To assess the silver-ion induced toxic effect on the *S. aureus*, Ag^+^ ion, released in phosphate-buffered saline (PBS) and plate counting methods was employed in their study (Figure 21a,b). From Figure 21a, all Ag-doped TNTs samples showed excellent antibacterial activity compared to commercially pure Ti (cpTi) and pristine TNTs. This was attributed to the released Ag^+^ ions from the Ag-doped TNTs for bacterial inactivation (Figure 21b). Such an antibacterial effect was unrelated to photoactivity. A profound difference in bacterial reduction in terms of CFU was seen between neat TNTs and Ag-doped TNTs. 

### 5.2. Non-Metal Doping

**Figure 22:**
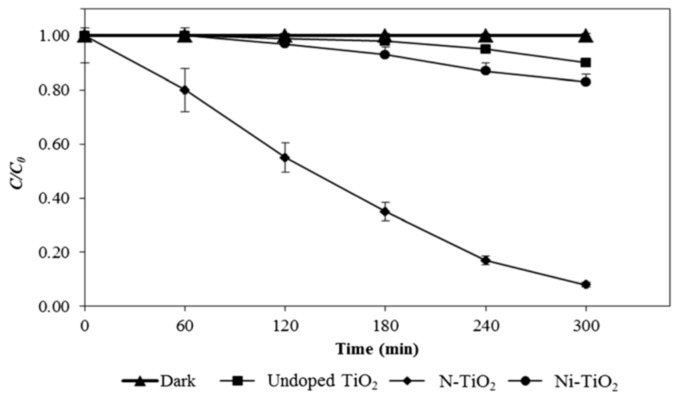
Survival ratio (C/Co) of *S. aureus* with neat TiO_2_, N-doped TiO_2_ NPs and Ni-doped TiO_2_ NPs under an 18 W visible light irradiation for different time periods. *S. aureus* without TiO_2_ in the dark is used as a control. Statistically significant at *p* < 0.05. Reproduced with permission from [161]. Copyright Springer, 2016.

**Figure 23:**
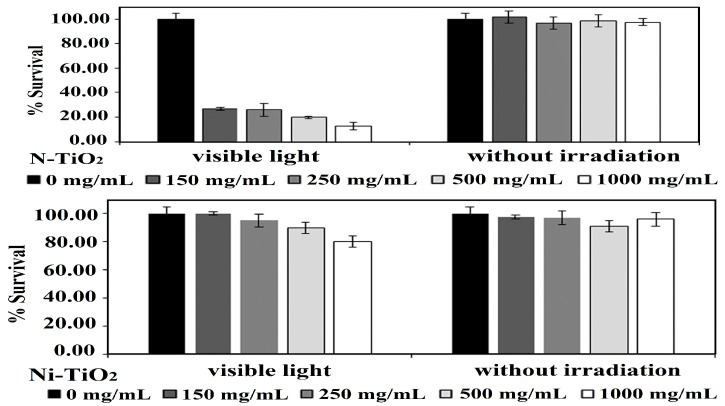
Photocatalytic inactivation of *S. aureus* with N- and Ni-doped TiO_2_ NPs of different contents under visible light. Statistically significant at *p* < 0.05. Reproduced with permission from [161]. Copyright Springer, 2016.

**Figure 24:**
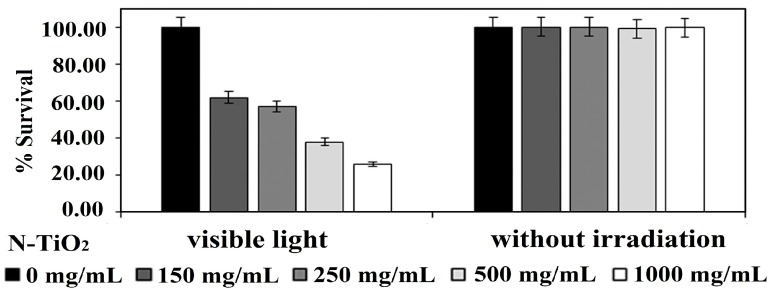
Photocatalytic inactivation of *E. coli* with N-doped TiO_2_ NPs of different concentrations under an 18 W visible light. Statistically significant at *p* < 0.05. Reproduced with permission from [161]. Copyright Springer, 2016.

Non-metal dopants such as nitrogen, carbon, and boron are typically employed to replace lattice oxygen anions of titania, in order to narrow its bandgap and extend the optical absorption edge to the visible regime, thereby increasing photocatalytic activity. This in turn leads to an improvement in its antibacterial properties under visible light [161,202,246,262,263,264,265]. The visible light response originates from the presence of localized energy levels of the dopant lying above the valence band, thus shifting the VB level upward (Figure 8) [97,159,160,161,162,163,164]. Among those dopants, nitrogen has a size comparable to oxygen, so it can be readily doped into the TiO_2_ lattice in either substitutional or interstitial sites. The N-2p orbital hybrids with the O-2p state, leading to the band gap narrowing. Recently, Ananpattarachai et al. prepared N-doped TiO_2_ NPs using the sol-gel technique with diethanolamine acting as the N source. For the purposes of comparison, they also prepared Ni-doped TiO_2_ NPs by adding NiSO_4_(H_2_O)_6_ to the Ti-sol. The as-synthesized N- and Ni- doped TiO_2_ NPs powders were calcined at 600 °C [161]. The bandgaps of N-doped TiO_2_ NPs and Ni-doped TiO_2_ NPs were determined to be 2.1 eV and 2.97 eV, respectively. The antibacterial activities of the photocatalysts were assessed using *S. aureus* and *E. coli* strains under visible light irradiation. Figure 22 shows the photocatalytic inactivation of *S. aureus* with neat TiO_2_, N-doped TiO_2_ NPs and Ni-doped TiO_2_ NPs. Apparently, N-doped TiO_2_ NPs are more effective than Ni-doped TiO_2_ NPs for *S. aureus* inactivation due to their smaller bandgap. Nearly 90% of *S. aureus* cells are inactivated by N-doped TiO_2_ NPs within 300 min. The complete inactivation time for *S. aureus* is 360 min. In contrast, the complete inactivation time for *E. coli* is 420 min (not shown). Figure 23 displays the photocatalytic inactivation of *S. aureus* with different concentrations of N-doped TiO_2_ NPs and Ni-doped TiO_2_ NPs. The survival of *S. aureus* with N-doped TiO_2_ NPs under visible light is smaller than with Ni-doped TiO_2_ NPs. Figure 24 shows the photocatalytic inactivation of *E. coli* with different concentrations of N-doped TiO_2_ NPs. The inactivation efficacy of Gram-positive *S. aureus* using N-doped TiO_2_ NPs is higher than that of Gram-negative *E. coli* under visible light illumination. According to the literature, carbon dopant is also beneficial in improving the visible light absorption of TiO_2_ NPs and photocatalytic inactivation of anthrax, a fatal bacterial disease that occurs in animals and can transmit to humans, and is caused by Gram-positive Bacillus anthracis [264].

**Figure 25:**
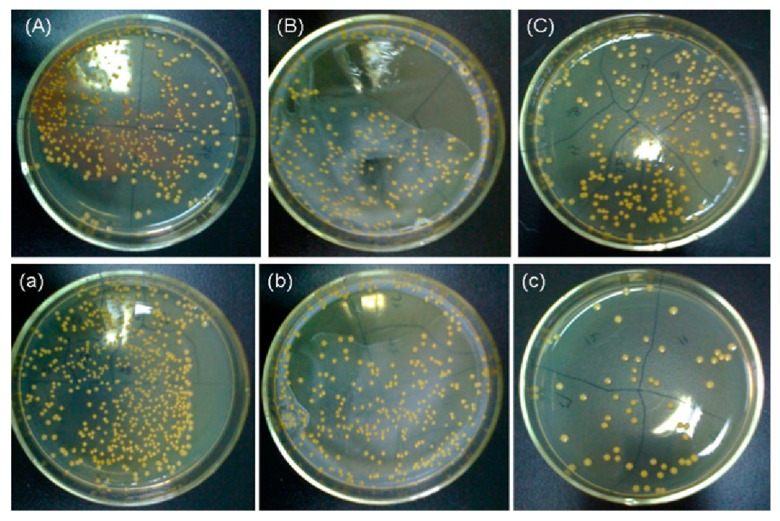
Photographs of *E. coli* colonies developed on agar plates treated with (**A**) control, (**B**) TiO_2_ and (**C**) N-doped TiO_2_ samples in the dark for 24 h. (**a**–**c**) are the images of *E. coli* colonies under visible light irradiation for 2 h. (**a**): control, (**b**): neat TiO_2_, and (**c**) N-doped TiO_2_. Reproduced with permission from [265]. Copyright Springer, 2013.

He et al. fabricated N-doped TiO_2_ NPs (30 nm) using the sol-gel technique. The photocatalytic and bactericidal behaviors of N-doped TiO_2_NPs against *E. coli* under dark and simulated-sunlight conditions were investigated [265]. The bacterial inactivation of this catalyst reaches 90% under simulated sunlight for 2 h, much higher than in the dark. Figure 25 displays photographs of neat TiO_2_ and N-doped TiO_2_ NPs treated with *E. coli* in the dark for 24 h, and under visible light for 2 h. Bacteria grows into colonies on these samples in the dark (top panel). However, N-doped TiO_2_ can inactivate *E. coli* by irradiating with simulated-sunlight for 2 h (bottom panel). 

**Figure 26:**
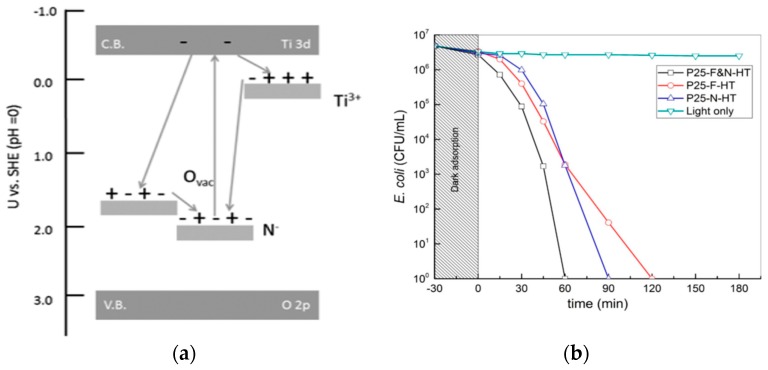
(**a**) Visible light excitation of N−F codoped TiO_2_ and subsequent filling of empty N state by electron transfer from either Ti^3+^ or oxygen vacancies (O_vac_). Reproduced with permission from [268]. Copyright American Chemical Society, 2014. (**b**) Survival rate of *E. coli* treated with N-doped P25 (P25-N-HT), F-doped P25 (P25-F-HT) and (3) F-N codoped P25 (P25-F&N-HT) under simulated light illumination. Reproduced with permission from [270]. Copyright MDPI, 2017.

From the literature, fluorine doping does not shift the bandgap of TiO_2_. Fluorine dopant stabilizes anatase TiO_2_ up to 1200 °C [75,266]. The replacement of lattice oxygen with fluorine in TiO_2_ converts Ti^4+^ to Ti^3+^ as a result of the charge compensation between F^−^ and Ti^4+^ [165]. The Ti^3+^ ions suppress the recombination rate of photogenerated charge carriers, thereby enhancing photocatalytic activity. The substitution of fluorine for oxygen in the titania lattice gives rise to a dramatic increase in oxygen vacancy concentrations [175,267]. The visible-light photocatalytic activity of F-doped TiO_2_ can be further enhanced by co-doping with N [268]. Thus, the co-doping approach is an effective route to tune the energy band level of TiO_2_ NPs to enhance photocatalytic reactions. Figure 26a shows the charge-transfer mechanism for visible-light excitation of N−F co-doped TiO_2_. A series of charge transfer events take place during visible light irradiation. In the process, electrons are excited from the N midgap state to the conduction band, and the corresponding holes generated in the N-state are filled by electrons from the Ti^3+^ level. Oxygen vacancies (O_vac_) also donate electrons to the empty N-state. Moreover, the conduction band can transfer electrons to the oxygen vacancies. This cascade effect facilitates the continuous generation of superoxide anion and hydroxide radical species [268,269]. The ROS then cause the destruction and death of microorganisms accordingly. 

Milosevic et al. fabricated N-doped and F-doped TiO_2_ by wet milling Aeroxide^®^ P25 (21 nm) powders in the presence of glycine (N source) or polytetrafluoroethylene (PTFE; fluorine source), respectively [270]. For the preparation of F−N-co-doped P25, both glycine and PTFE were added during wet milling. The resulting N-doped P25 was heat-treated at 500 °C for 1 h, while F-doped P25 and F−N-doped P25 were calcined at 600 °C for 1 h. Figure 26b shows the survival rate of *E. coli* treated with F-doped P25, N-doped P25, and F−N-co-doped P25 under visible light irradiation. The complete bacterial inactivation times for F-doped P25, N-doped P25 and F−N-co-doped P25 catalysts are 120 min, 90 min and 60 min, respectively. Apparently, codoping P25 with F and N leads to the F−N-doped P25 catalyst with the best bactericidal performance. This is attributed to a synergistic effect between F and N dopants, creating a series of charge transfer reactions, thereby inducing ROS for bacterial inactivation (Figure 26a). In another study, Milosevic et al. prepared F-doped TiO_2_ NPs by means of solution precipitation through the hydrolysis of titanium oxychloride, using urea and ammonia as the precipitation agents, and potassium fluoride as the fluorine source. This was followed by wet milling in glycine, and the resulting F−N-doped TiO_2_ powders were calcined at 500 °C for 1 h [271]. The complete bacterial inactivation times for F-doped TiO_2_ and F-N codoped TiO_2_ are 75 min and 60 min, respectively. 

### 5.3. Graphene and MWNT Modified Titania Nanocomposites

A graphene sheet with sharp edges can act as a ‘nanoknife’ for killing microorganisms during the direct contact of bacteria with the sheet edges. In addition, graphene with a lateral dimension of several micrometers can effectively wrap and isolate bacteria from the environment, thus stopping the supply of nutrients [272,273]. A direct contact of the bacterial cell wall with graphene may also lead to the induction of oxidative stress, resulting in the physical disruption of lipid bilayers and the generation of ROS [274,275]. In this respect, the inclusion of rGO to TiO_2_ NPs can produce novel photocatalysts with improved antibacterial performance. There are few studies in the literature reporting bacterial inactivation of rGO/TiO_2_ photocatalysts [101,102,276,277]. 

**Figure 27:**
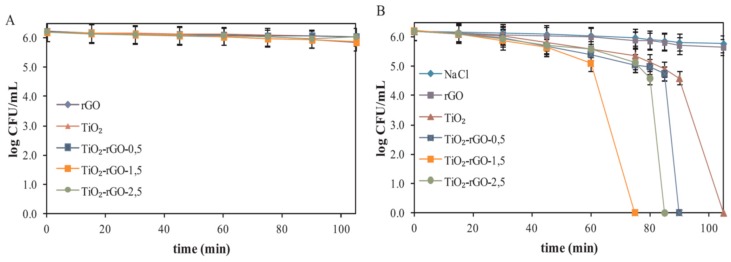
Inactivation of *E. coli* in the presence of rGO, TiO_2_ and (0.5–2.5 wt%) rGO/TiO_2_ samples in (**A**) dark contition and (**B**) under artificial solar light irradiation. Reproduced with permission from [101]. Copyright Elsevier, 2018.

More recently, Wanag et al. fabricated (0.5–2.5 wt%) rGO/TiO_2_ NPs using hydrothermal process at 180 °C for 4 h. The resulting products were finally heated in a furnace at 100 °C for 4 h [101]. Figure 27A,B shows the survival rate of *E. coli* treated with (0.5–2.5 wt%) rGO/TiO_2_ NPs in the dark and under artificial solar light irradiation, respectively. Little change in the bacterial populations is seen in the dark condition. The complete bacterial inactivation times for TiO_2_ NPs, 0.5% rGO/TiO_2_ NPs, 1.5% rGO/TiO_2_ NPs, and 2.5% rGO/TiO_2_ NPs samples are 105, 90, 75 and 85 min, respectively. It appears that the 1.5% rGO/TiO_2_ NPs photocatalyst exhibits the best bacterial inactivation effect. 

**Figure 28:**
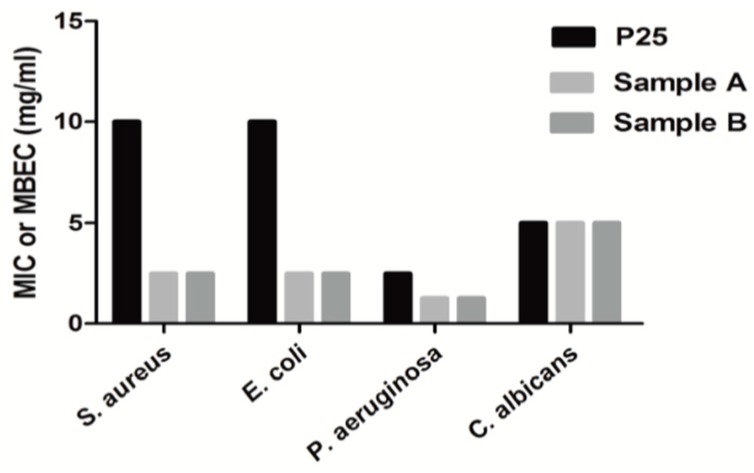
MIC and MBEC values of rGO/1%Fe-N-doped TiO_2_ nanocomposites and commercial P25 TiO_2_ treated with *S. aureus*, *E. coli*, *P. aeruginosa* and fungal C. albicans under visible light at 37 °C for 24 h. Reproduced with permission from [277]. Copyright MDPI, 2017.

Nica et al. determined the minimum inhibitory concentration (MIC) and minimum biofilm eradication concentration (MBEC) values of rGO/1%Fe–N-doped TiO_2_ nanocomposites prepared by a hydrothermal synthesis of mixed 1%Fe–N-doped TiO_2_ and GO solutions at 150 °C for 2 h [277]. Two different precipitation strategies were adopted to yield 1%Fe–N-doped TiO_2_ powders. Sample A solution was prepared through a simultaneous precipitation of Ti^3+^ and Fe^3+^ ions by mixing desired amounts of TiCl_3_ and FeCl_3_.6H_2_O in water, with a subsequent addition of NH_4_OH to maintain an alkaline pH of 9. This solution was hydrothermally treated at 200 °C for 2 h, and the resulting powders were calcined at 400 °C for 2 h. Sample B solution was made via a sequential precipitation of these two cations. The Ti^3+^ ions were first precipitated and oxidized to Ti^4+^ followed by the addition of Fe^3+^ ions in an alkaline reaction medium. The purpose of this was to attain a higher iron concentration on the surfaces of synthesized powders [242]. The antibacterial activity of rGO/1%Fe–N-doped TiO_2_ nanocomposites was assessed with *S. aureus*, *E. coli*, *P. aeruginosa* and Candida albicans (a type of fungus). Figure 28 shows the the MIC and MBEC values of rGO/1%Fe–N-doped TiO_2_ nanocomposites irradiated with visible light. MIC is generally defined as the lowest antimicrobial agent concentration inhibiting visible growth of bacteria, whereas MBEC is the lowest concentration of an antimicrobial agent needed to kill a bacterial biofilm [278,279]. Compared with commercial P25, rGO/1%Fe–N-doped TiO_2_ nanocomposites made from the respective Sample A and Sample B solutions exhibit a much higher antibacterial activity under visible light exposure than Gram-positive *S. aureus*, Gram-negative *E. coli*, and *P. aeruginosa*. The MIC value of rGO/doped TiO_2_ nanocomposites for *E. coli* and *S. aureus* is 2.5 µg/mL, four times smaller than that of P25. 

Akhavan et al. prepared MWNT/TiO_2_ thin films with 2–40 wt% MWNTs by sol-gel technique. The films were deposited on the glass slides by the dip coating method followed by annealing at 450 °C in air for 1 h to yield anatase phase, thereby forming Ti–C and Ti–O–C bonds [155]. The 20 wt% MWNT/doped TiO_2_ film inactivated *E. coli* completely under visible light irradiation for 60 min. They attributed bactericidal effects of 20 wt% MWNT/doped TiO_2_ nanocomposite to an efficient charge transfer between the MWNTs and TiO_2_ due to the formation of a Ti–C and Ti–O–C bond, and a reduction in the electron–hole recombination rate, leading to an increase in the production of hydroxyl radicals for photocatalytic inactivation. Very recently, Koli et al. fabricated (0.1–0.5 wt%) MWNT/doped TiO_2_ nanocomposites using the solution-mixing method. The final products were centrifuged and calcinated in air at 450 °C for 5 h [156]. The 0.5 wt% MWNT/doped TiO_2_ nanocomposite exhibited complete killing for *S. aureus* and *E. coli* under visible light irradiation for 180 and 300 min, respectively. However, nanocomposites with 0.3 wt% and 0.1 wt% MWNTs only showed 80% and 90% inhibition for *S. aureus*. In another study, Koli et al. prepared (0.1–0.5 wt%) MWNT/Fe-doped TiO_2_ nanocomposites using the sol gel process. The 0.5 wt% MWNT/Fe-doped TiO_2_ nanocomposite showed 100% inactivation for Gram-positive Bacillus subtilis under visible light illumination for 120 min. However, nanocomposites with 0.1 wt% and 0.3 wt% MWNTs exhibited complete inhibition at 180 min [157]. In the case of Gram-negative Pseudomonas aeruginosa, MWNT/Fe-doped TiO_2_ nanocomposites with 0.1, 0.3 and 0.5 wt% MWNTs required 240 and 300 min, respectively, for complete bacterial killing. The photocatalytic inactivation of these bacterial strains derived from the effective generation of ROS under visible light irradiation.

### 5.4. Coupled Semiconductors

**Figure 29:**
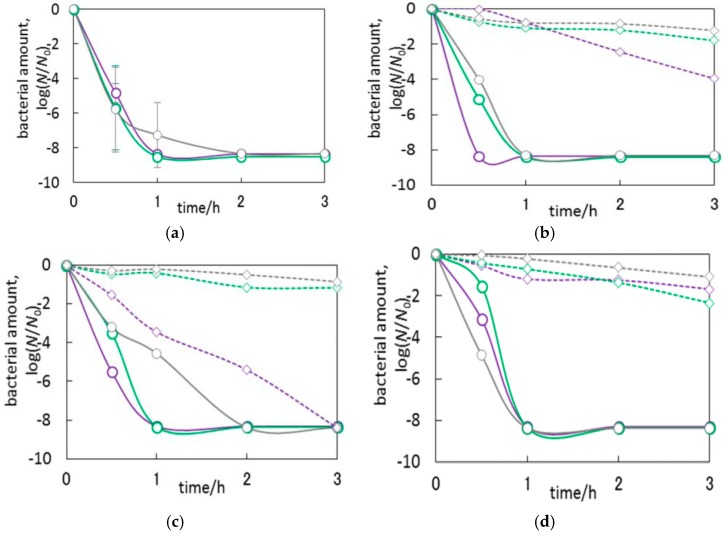
Survival of *E. coli* in CFU/mL treated with (**a**) Cu_2_O, (**b**) Cu_2_O/anatase TiO_2_, (**c**) Cu_2_O/P25, and (**d**) Cu_2_O/rutile TiO_2_ in solid curves with circle symbols; grey color (dark condition), violet (UV irradiation) and green (visible light). Umodified anatase TiO_2_, P25 and rutile TiO_2_ results are shown in dashed curves with diamond symbols in grey (dark condition), violet (UV irradiation) and green (visible light). Error bars in Cu_2_O: standard deviation determined from two or three independent measurements. Reproduced with permission from [177]. Copyright MDPI, 2018.

Limited studies are available in the literature on the visible-light photocatalytic bactericidal activity of oxide semiconductor heterojunctions [177,179]. Recently, Janczarek et al. studied the antibacterial performance of cuprous oxide/titania nanocomposites prepared by mechanically mixing Cu_2_O with TiO_2_ powders of different structures including pure anatase TiO_2_ (8 nm), pure rutile TiO_2_ (16 nm) and Aeroxide^®^ TiO_2_ P25 in an agate mortar [177]. Figure 29a–d shows the antibacterial performance of pure Cu_2_O and Cu_2_O/TiO_2_ nanocomposites against *E. coli* in the dark, under UV and visible light irradiation, respectively. Pure Cu_2_O displays high bactericidal activity under UV or visible light irradiation due to the intrinsic activity of Cu^+^ ions (Figure 29a). Pure anatase TiO_2_ NPs with a negative charge surface show little bacterial inactivation in the dark as they repel negatively charged bacteria to a lesser degree [44,57]. However, their antibacterial activity improves substantially under UV light irradiation as a result of the ROS generation. By coupling anatase TiO_2_ with Cu_2_O, the Cu_2_O/anatase shows enhanced antibacterial activity in the dark, under UV or visible light irradiation (Figure 29b; solid curve). The bactericidal activity of Cu_2_O/anatase in the dark is caused by the Cu^+^ ions in Cu_2_O. The enhanced bactericidal activity of this sample under visible light is attributed to the interfacial charge transfer of electrons from Cu_2_O to TiO_2_ across the heterojunction interface. This prolongs the lifetime of charge carriers, such that they can take part in photocatalytic reactions. Cu_2_O generates electron–hole pairs readily under visible light irradiation due to its small bandgap of 2.17 eV. Under UV light irradiation, the interfacial charge transfer from TiO_2_ to Cu_2_O, and the inhibition of charge carriers’ recombination contribute to an enhancement in bactericidal activity. In contrast, unmodified rutile and Cu_2_O/rutile show slower antibacterial activity under UV or visible light irradiation (Figure 29d).

### 5.5. Polymer/Titania Nanocomposites

Novel polymer nanocomposites with enhanced chemical, thermal and mechanical properties can be designed and developed by adding functional nanofillers of unique properties [26,26,68,69,70,71,72,280,281]. Such polymer nanocomposites show great potential for structural, electronic, environmental and biomedical engineering applications. The polymeric matrix of nanocomposites immobilizes nanofillers and offers protection to the fillers from mechanical damage. The extent of property enhancement in the polymer nanocomposites depends greatly on the homogeneous dispersion of the fillers, and good interfacial bonding between the filler and polymer matrix [280]. The polymers employed for forming nanocomposites can be either degradable or nondegradable, depending on their intended application. Degradable polymers such as PLA, polyvinyl alcohol (PVA), poly(ε-caprolactone) (PCL) and chitosan are generally used to form scaffolds and wound dressings [26,65,282,283,284,285]. These polymers should have the ability to degrade with time and to heal the wounds. 

Titania nanomaterials have been incorporated into polymers to form antibacterial coatings, food packaging materials, medical implants, wound dressings and scaffolds [59,60,61,62,63,64,65,229,286,287]. However, those studies are mainly focused on the bactericidal properties of the polymer nanocomposites with TiO_2_ nanomaterials under UV irradiation. However, it is impractical to use a UV source to excite electron–hole pairs in the polymer–TiO_2_ NP system for applications in theb medical sector and food industry. Therefore, the development of visible-light active polymer–TiO_2_ nanocomposites is considered of technological interest and practical importance [288,289,290,291,292,293].

**Figure 30:**
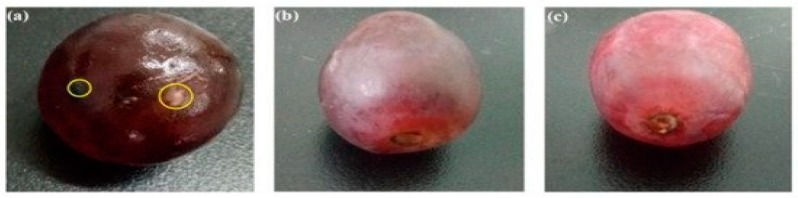
Photographs showing the preservation of red grapes wrapped with (**a**) plastic film, (**b**) chitosan film, and (**c**) chitosan-TiO_2_ film at 37 °C for six days. Reproduced with permission from [289]. Copyright Elsevier, 2017.

The bactericidal activity of polymer–TiO_2_ nanocomposites under visible light also depends on the type of polymers employed. In particular, natural chitosan (CS) with biodegradable behavior can bind to TiO_2_ NPs through its amino and hydroxyl groups, thereby extending the optical absorption of TiO_2_ NPs into the visible region. The CS/TiO_2_ nanocomposites exhibit a red shift in absorption in their UV-vis spectra [289,292,294]. The bandgap of TiO_2_ NPs in CS/TiO_2_ nanocomposites is then reduced from 3.20 to 3.00 eV [292]. As such, CS/TiO_2_ nanocomposites exhibit the photocatalytic inactivation of microorganisms under visible light. Accordingly, CS/TiO_2_ films find useful application as antimicrobial wrapping films for vegetables and fruits under visible light. Thus, TiO_2_ nanofillers can delay the ripening process of fresh produce [289,295]. Very recently, Zhang et al. fabricated a CS/TiO_2_ nanocomposite film for wrapping red grapes. They assessed the antimicrobial activity of the film against food-borne pathogenic microbes, including *E. coli*, *S. aureus*, C. albicans, and Aspergillus niger (mold) [289]. They found that the film exhibited a good microbial inactivation effect, with complete sterilization for all microbial strains within 12 h. The composite film was very effective in protecting red grapes from microbial attack, thereby extending their shelf-life and improving the quality of fresh fruit accordingly (Figure 30a–c). Moreover, pure CS film also shows antibacterial effect to a certain degree. In contrast, grapes were spoiled and mouldy in the plastic wrap, as expected. 

**Figure 31:**
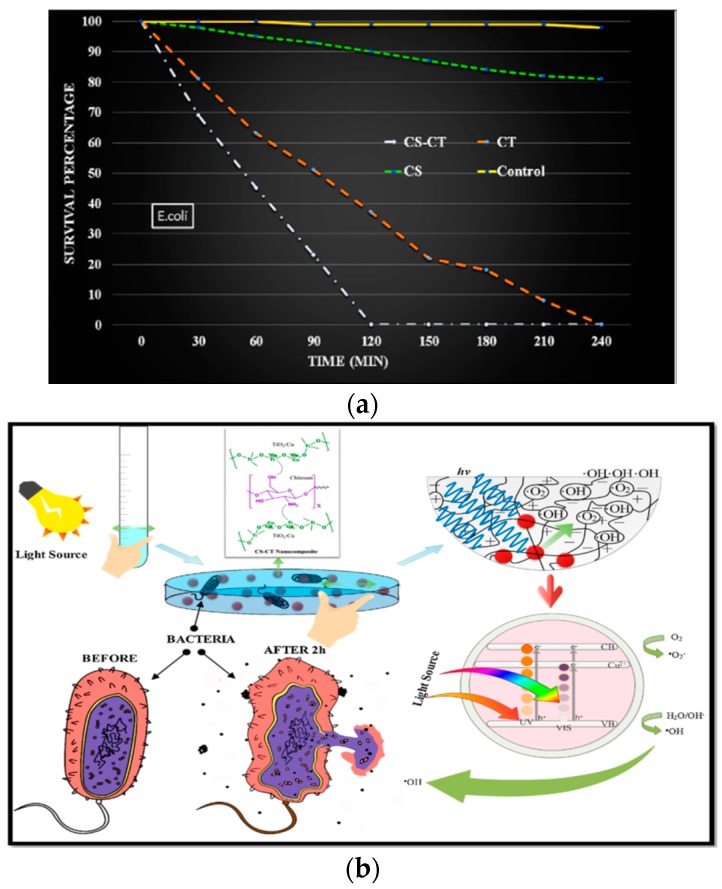
(**a**) Antibacterial activity of CS, CT and CS–CT samples against *E. coli* under visible light illumination. (**b**) Mechanism of antibacterial activity of CS/Cu-doped TiO_2_ nanocomposite. Reproduced with permission from [288]. Copyright Elsevier, 2011.

It is noteworthy that CS/TiO_2_ nanocomposites also find medical applications as antibacterial scaffolds in the presence of visible light. Biodegradable chitosan has been used as a scaffold in orthopedics, particularly for bone tissue engineering. The hydrophilic behavior of CS facilitates the adhesion and growth of bone cells (osteoblasts) on its surface [296]. However, a pure chitosan scaffold suffers from poor mechanical strength, so it is unable to provide sufficient mechanical support for the proliferation of osteoblasts during the bone healing process. Therefore, TiO_2_ NPs and AgNPs are added to CS to improve mechanical strength [65]. Recently, Raut et al. enhanced the visible-light bacterial inactivation of the CS/TiO_2_ NPs nanocomposite by including a Cu dopant into TiO_2_ NPs (denoting as CT) through the sol-gel process. The CT nanofillers were then solution-mixed with CS to yield CS/Cu-doped TiO_2_, denoted as CS-CT [288]. Figure 31a shows the bactericidal activity of CS, CT and CS–CT samples against *E. coli* under visible light. A 100% bacterial inactivation time is achieved in 240 min by CT and 120 min by CS–CT. Therefore, a synergistic effect exists between Cu-doped TiO_2_ NPs (CT) and C, thereby giving rise to a faster bacterial reduction time of 120 min. Figure 31b shows the effect of an *OH radical, generated by the photoexcitation of an electron–hole pair, in destroying *E. coli*. 

**Figure 32:**
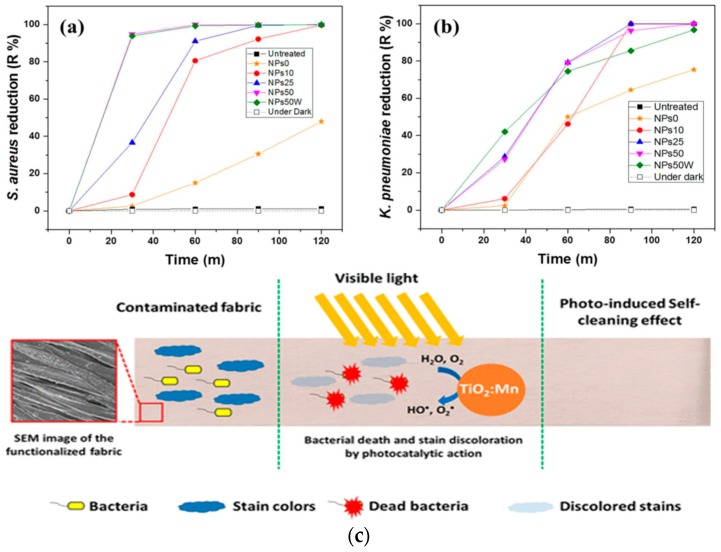
Bacterial reduction in percentage of (**a**) *S. aureus* and (**b**) *K. pneumoniae* on cotton fabrics with and without Mn-doped TiO_2_ NPs in the dark and under natural sunlight. NPs0, NPs10, NPs25 and NPs50 are cotton fabrics with zero, 10, 25 and 50 wt% Mn-doped TiO_2_ NPs; NPs50W is the NPS50 after 10 washing cycles. (**c**) Schematic of visible light induced the photocatalytic inactivation of bacteria and degradation of stain residues on contaminated fabric with Mn-doped TiO_2_ NPs. Reproduced with permission from [298]. Copyright American Chemical Society, 2018.

Antibacterial polymer/TiO_2_ nanocomposites also find attractive applications in textile industries for producing odorless and self-cleaning fabrics. Clothing fabrics are prone to microbial contamination, and can spread infections accordingly. The clothes generate a warm and humid environment on human skin, causing the secretion of sweat and bacterial growth [297]. Cotton consists of natural cellulosic fibers that absorb more sweat than synthetic polymer fabrics. Thus, cotton clothes tend to keep the body dry while absorbing sweat. Very recently, Zahid et al. prepared Mn-doped TiO_2_ NPs (150 nm) by the sol-gel method, and then applied the spray coating technique to apply Mn-doped TiO_2_ NPs on cotton fabrics. NPs0, NPs10, NPs25 and NPs50 were designated to the cotton fabrics with zero, 10, 25 and 50 wt% Mn-doped TiO_2_ NPs, respectively [298]. The fabrics with and without Mn-doped TiO_2_ NPs in the dark exhibited no bactericidal effect because no photocatalytic excitation occurred in the absence of light. The NPs10 and NPs 25 reduced the *S. aureus* population by 80% and 90% within the first 60 min, respectively, while NPs50 and NPs50W reduced nearly ~100% *S. aureus* population in the same period under sunlight (Figure 32a). A lower bacterial inactivation rate was found for the fabrics with Mn-doped TiO_2_ NPs and treated with *K. pneumoniae* in the first 60 min (Figure 32b). The presence of Mn ions dopant promoted the generation of an electron–hole pair in TiO_2_ fillers for creating ROS under visible light, as shown in Figure 32a,c. Apart from its antimicrobial activity, the photocatalytic effect also removed or degraded color stains on the fabrics, thus performing self-cleaning, as shown in Figure 32c. The visible-light antibacterial activity of modified titania nanomaterials is summarized in Table 2.

## 6. Biocompatibility and Cytotoxicity

Antibacterial materials and coatings have been a focus of global research topics for the past decade in orthopedics, due to an increasing incidence of implant-related infections caused by MDR bacteria [299]. Medical devices and implants are easily contaminated with microorganisms, leading to the formation of biofilms on their surfaces. Therefore, titania coating is an attractive solution for controlling implant infection by decreasing bacteria adhesion through the introduction of metal nanoparticles (e.g., AgNPs or CuNPs) to the coating [300,301]. Furthermore, titania coating formed on the medical devices should exhibit good biocompatibility with the host tissues. As an example, titania film deposited on polyetheretherketone (PEEK) spinal implant exhibits superior compatibility compared to uncoated PEEK in terms of osteoblastic adhesion, proliferation, and differentiation [302]. Similarly, anodic TNTs of large surface areas formed on Ti-based alloys have been reported to provide anchoring sites for osteoblasts and fibroblasts, thereby promoting cell proliferation effectively [217,218,219,303,304,305]. Ti-based alloys (e.g., Ti-6%Al-4%V and Ti-6%Al-7%Nb) are commonly used as the load-bearing prostheses in orthopedics and tooth implants in dentistry. The optimal diameter of TNTs for osteoblastic adhesion and growth is typically below 100 nm [217,218]. Recently, Radtke et al. reported that anodic TNTs formed on the Ti-6%Al-4%V alloy, especially at lower anodic potentials with diameters of 25–35 nm, were effective in promoting the growth of murine fibroblasts L929 [303]. Xu et al. investigated the biocompatibility of periodontal ligament cells on anodized TNTs of different diameters. Periodontal ligament cells (PDLCs) are the key cells responsible for periodontal tissue regeneration [304]. They demonstrated that the TNTs formed on thin Ti foil substrate favor the adhesion and growth of PDLCs. Furthermore, TNTs promoted the osseointegration of Ti substrate more effectively than untreated Ti foil, as evidenced by the high gene expression levels of alkaline phosphatase, osteocalcin and osteopontin.

### 6.1. Cytotoxicity 

Advanced nanomaterials, prepared by emerging nanotechnology, pose toxicity to humans to a large/lesser extent depending on their structure, chemical composition, shape, distribution, etc. [14,15,306]. Wadhwa et al. reported that hydrothermally synthesized TNTs and TiO_2_ NPs exhibit no toxic effect towards the human alveolar carcinoma epithelial cell line (A549) [307]. Standalone and detached TNTs from Ti foil substrate were toxic to human dermal fibroblasts as a result of the ROS generation, leading to DNA damage and chromosomal aberration [308]. Allegri et al. reported that electrospun TiO_2_ nanofibers were toxic towards A549 and murine macrophage cell lines (Raw 264.7). The cytotoxic effects were dose-dependent, with larger effects on A549 than on Raw 264.7. However, TiO_2_ NPs exert no cytotoxic effect on these two cell lines [309]. 

As mentioned, TiO_2_ NPs have been produced commercially in large quantities for applications in paints, pharmaceutical, food, drug and cosmetic industries [39,40,41,42,43,44,45,46,47,48,49,50,51,52,53,54,55,56]. The synthesis and handling of TiO_2_ NPs during the production process can release a tremendous amount of these nanomaterials into the environment, including air, soil and water. The wastewater of titania production plants is the major source pollutant that leaks into the environment. The nanomaterials are discharged in the sewage sludge and can enter the soil and water ecosystem [310]. Therefore, the disposal and treatment of wasterwater, as well as the recycling of titania from water treatment plants, are considered of technological importance [311]. Moreover, the widespread use of products with TiO_2_ NPs would also release such nanoparticles into the environment. For instance, auto-manufacturing plants consume large quantities of titania pigment paints for coating car bodies. Therefore, plaint sludge is always found in the paint-bearing wastewaters. The recycling and reproduction of TiO_2_ NPs from the paint sludge are beneficial to both the environmental and industrial sectors for minimizing pollution [312]. Furthermore, embedded TiO_2_ NPs in the paint of buildings, bridges and traffic railings could be lost and released into the aquatic environment due to a lengthy outdoor weathering [313]. Some consumer titania products such as food-packages and textiles could also end up as waste, and are disposed of in incinerators and landfills [314]. The wastewater treatment plants remove most TiO_2_ NPs in the influent sewage; however, the residual nanoparticles would discharge to natural water ecosystems. In this respect, TiO_2_ NPs could potentially induce harmful effects to aquatic organisms, such as impaired metamorphosis and growth, teratogenicity, and mortality of fish larvae [315,316]. 

#### 6.1.1. Neat TiO_2_ NPs

TiO_2_ NPs can enter the human body through exposure to workplace atmospheres, the use of commercial products, the ingestion of food and pharmaceuticals. The main routes of entry include skin contact, inhalation, ingestion and medical implants [317]. Accordingly, public concerns have been raised related to the safe use of TiO_2_ NPs, and their effects on human health and the environment. Conflicting results are reported in the literature regarding the biocompatibility and cytotoxicity of TiO_2_ NPs. Some studies indicate a good biocompatibility of TiO_2_ NPs with mammalian cells [166,201,309,318], while others reveal the toxic effects of TiO_2_ NPs [319,320,321,322,323,324,325]. The discrepancy is attributed to the biological and materials factors involved during in vitro studies. The former factors include the type of culture cells, cell cultivation time and cell-based assays used, while the latter factors include the size, shape, crystal structure (anatase or rutile), and dose of TiO_2_ NPs in the assay tests [326]. 

Wang et al. investigated cytotoxicity in A549 cells induced by TiO_2_ NPs (5 nm) using 3-(4,5-dimethylthiazol-2-yl)-2,5-diphenyltetrazolium bromide (MTT), quantitative real-time PCR (qRT-PCR) and comet assays, as well as rhodamine 123 staining [323]. From the MTT results, the cytotoxic effect of TiO_2_ NPs was time- and concentration-dependent (in a range from 50 to 200 μg/mL). Comet assay revealed DMA damage in cells exposed to 50 to 200 μg/mL TiO_2_ NPs for 48 h. TiO_2_ NPs also decreased the mitochondrial membrane potential as determined by rhodamine 123 (Rh123) staining. The qRT-PCR analysis demonstrated that the expression of caspase-3 and caspase-9 messenger RNA (mRNA) increased dramatically upon exposure to 100 and 200 μg/mL TiO_2_ NPs for 48 h. As is known, caspases play the key role in executing apoptosis. Caspase-3 tends to induce DNA bond cleavage and inactivate cytoskeletal proteins. From this perpective, TiO_2_ NPs inhibit A549 proliferation, and induce DNA damage and eventual cell apoptosis through the activation of the intrinsic mitochondrial pathway [323]. 

TiO_2_ NPs can penetrate the human body through inhalation, then translocate from the lungs into the bloodstream, and the subsequent uptake in other organs like the heart, liver, spleen and brain. Huerta-Garcia et al. investigated the in vitro toxicity of TiO_2_ NPs on rat cardiomyoblasts (H9c2) [327]. They found that the cellular uptake of TiO_2_ NPs by H9c2 cells reduces their metabolic activity and cell growth, thus causing mitochondrial dysfunction due to a marked reduction in mitochondrial membrane potential. Furthermore, TiO_2_ NPs increase the ROS level and membrane permeability of H9c2 cells greatly, leading to final cell death. Therefore, the internalization and building up of TiO_2_ NPs in cardiomyoblasts would induce cardiac damage and pose threats to human health upon inhalation of those nanoparticles. More recently, Mottola et al. studied the genotoxic effect of TiO_2_ NPs on human amniotic fluid cells in vitro. The TiO_2_ NPs exposure caused DNA strand fragmentation, a loss of viability, and apoptosis of the cells [325]. 

Yin et al. studied the phototoxicity of TiO_2_ NPs (<25 nm, 31 nm, <100 nm, and 325 nm) in human skin keratinocytes (HaCaT) under UVA irradiation [328]. TiO_2_ NPs induced photocytotoxicity and cell membrane damage in a UVA dose- and TiO_2_ NPs dose-dependent manner. The smaller the size of TiO_2_ NPs, the higher the cell damage was. The induced photocytotoxic damage was attributed to the ROS generation during UVA irradiation, leading to lipid peroxidation of the plasma membrane. TiO_2_ NPs with a large surface-to-volume ratio enhanced biological reactivity by generating ROS. The degree of photocytotoxicity and cell membrane damage depends greatly on the level of ROS generated. More recently, Ren et al. also reported a similar finding on the phototoxicity of TiO_2_ NPs in HaCaT cells under UV irradiation [329]. 

**Figure 33:**
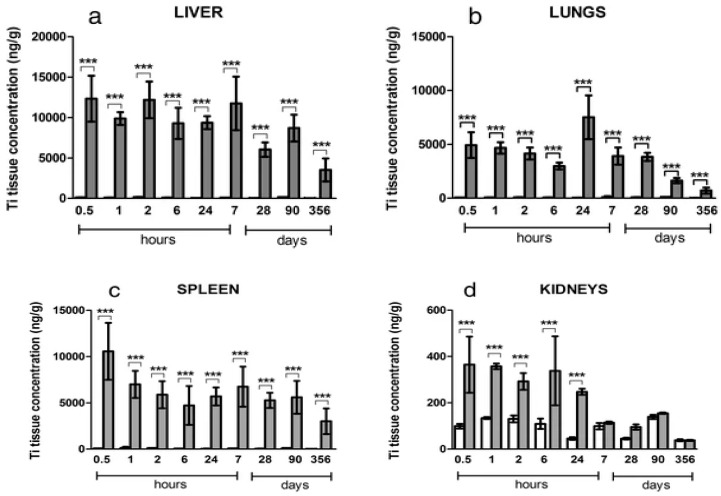
Biopersistence of titanium level in (**a**) liver, (**b**) lungs, (**c**) spleen and (**d**) kidneys after intravenous injection of 1 mg/kg TiO_2_ NPs in rats for 365 days. Grey and white bars are treated and control mice, respectively. Error bars represent the mean ± SD and *n* = 6. Statistical comparison is performed by two-way ANOVA, * *p* < 0.05; ** *p* < 0.01; *** *p*< 0.001. Reproduced with permission from [333]. Copyright BioMed Central, 2015.

The adverse effects of TiO_2_ NPs on mammalian cells, such as DNA damage, the generation of ROS, and apoptosis, addressed in in vitro studies, are supported by in vivo animal models [330,331,332,333,334]. Grassian et al. exposed mice to TiO_2_ NPs (2–5 nm; 8.88 mg/m^3^; 4 h/day for 10 days) through inhalation. Exposure for 1–2 weeks led to pulmonary inflammation with high cell counts of alveolar macrophages in bronchoalveolar lavage (BAL) fluid [330]. To evaluate the potential respiratory system toxicity, Liu et al. studied the biodistribution of TiO_2_ NPs (5, 21 and 50 nm) in rats via intratracheally instillation at doses of 0.5, 5, and 50 mg/kg body weight (bw). Rats were then sacrificed one week post-instillation [331]. Histopathological evaluation of lung tissues revealed a dose-dependent inflammatory lesion. At a specific dose, the pulmonary toxicity induced by 5 nm TiO_2_ NPs was more severe than that caused by 21 and 50 nm TiO_2_ NPs. In the case of dermal exposure, Wu et al. reported that TiO_2_ NPs can penetrate through the skin of hairless mice, and finally reach the liver following a prolonged exposure of 60 days. This led to a remarkable change in malondialdehyde (MDA) level [332]. MDA is a marker of liquid peroxidation and oxidation stress of cells. Therefore, an increase in free radicals produces high levels of MDA. Disdier et al. intravenously administrated P25 TiO_2_ NPs of 1 mg/kg into male Fisher F344 rats. They analyzed the biodistribution of Ti level in internal organs of rats using inductively coupled plasma mass spectrometry [333]. Biopersistence of Ti in the main target organs, i.e., the liver, lungs, spleen and kidneys, was observed after intravenous administration for up to 365 days (Figure 33a–d). Jia et al. intraperitoneally injected TiO_2_ NPs (5, 10, 60, 90 nm) and TiO_2_ microparticles at doses of 5, 10, 50, 100, 150, and 200 mg/kg (once a day for 14 days) into rats (half male and half female). TiO_2_ NPs were found to accumulate in the liver, kidney, spleen, lung, brain, and heart through the circulatory system [335]. The liver was damaged seriously due to mitochondrial dysfunction and the ROS generation at TiO_2_ NPs doses ≥10 mg/kg, leading to hepatocyte apoptosis. Furthermore, TiO_2_ NPs were more toxic than TiO_2_ microparticles, as expected. The distribution of TiO_2_ NPs in the brain tissue suggested that nanoparticles can enter directly into the central nervous system without crossing the blood–brain barrier. Finally, TiO_2_ NPs also caused genotoxicity in the ex vivo mouse embryo models [335].

#### 6.1.2. Metal-Doped TiO_2_ NPs

**Figure 34:**
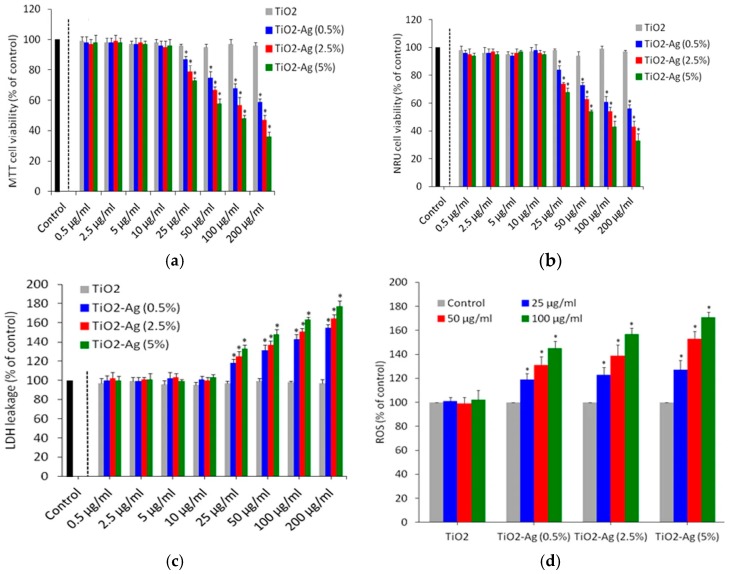
(**a**) MTT, (**b**) NRU, and (**c**) lactase dehydrogenase leakage results of human liver cancer (HepG2) cells exposed to TiO_2_ NPs and Ag-doped TiO_2_ NPs of different concentrations. (**d**) ROS level of HepG2 cells exposed to pure TiO_2_ NPs and Ag-doped TiO_2_ NPs of 25, 50 and 100 µg/mL. Reproduced with permission from [318], Copyright Nature Publishing Group, 2017.

We now consider the effect of metal doping on the cytotoxicity of TiO_2_ NPs in human cells. Doping TiO_2_ NPs with transition metals and noble metals is an efficient strategy for improving the photocatalytic inactivation of bacteria in the visible region. However, this approach has its own drawback as metal dopants can induce cytotoxicity in human cells. Therefore, metal-doped TiO_2_ NPs photocatalysts can fulfill the requirement of bactericidal performance, but they pose potential human health and safety hazards following long-term exposure. Recently, Ahamed et al. employed MTT and neutral red uptake (NRU) assays to assess the metabolic and lysosomal activities of human liver cancer (HepG2) cells exposed to Ag (0.5–5%)-doped TiO_2_ NPs [318]. Their results showed that Ag-doped TiO_2_ NPs reduce the cell viability in a dose-dependent manner, as shown in Figure 34a,b. Apparently, TiO_2_ NPs display no toxic effect to the HepG2 cell. In contrast, Ag (0.5–5%)-doped TiO_2_ NPs are toxic, as the Ag-dopant can release zero valent Ag^0^ or Ag^+^ ion to reduce cell viability through ROS generation [15]. This leads to the leakage of intracellular components at Ag (0.5–5%)-doped TiO_2_ NPs doses of ≥25 µg/mL, as evidenced by lactate dehydrogenase (LDH) assay (Figure 34c). Furthermore, cell viability decreases with increasing Ag content in Ag-doped TiO_2_ NPs, from 0.5% to 5%, while the ROS level increases with increasing Ag content (Figure 34d).

#### 6.1.3. rGO-Modified TiO_2_ NPs

Jin et al. demonstrated that rGO/TiO_2_ NPs would separate independently into TiO_2_ NPs and GO after entering A549 cells. The rGO/TiO_2_ NPs composite could induce cytotoxicity in A549 due to the generation of oxidative stress [336]. More recently, Prakash et al. prepared rGO (10–50%)/TiO_2_ nanocomposites using hydrothermal synthesis, and studied their toxicity in zebrafish embryos and larvae [337]. The toxicity of rGO (10–50%)/TiO_2_ nanocomposites was highly dependent on the rGO concentrations and doses. At low concentration and dose levels (10%, 20%, and 30% rGO; 0.25–30 μg/mL), rGO (10–30%)/TiO_2_ nanocomposites exhibited no toxicity to the zebrafish embryos. At high doses of 0.125–1.0 mg/mL, all the rGO (10–50%)/TiO_2_ nanocomposites induced teratogenicity and cardiotoxicity due to the generation of ROS. Zebrafish (Danio rerio) are aquatic species commonly employed to detect toxicological effects due to their known physiology having a high degree of genetic similarity with mammals, and the optical transparency of the tissues. 

## 7. Prospects and Challenges

Titania has been considered an inert and nontoxic material. It is typically used as a color additive for foods up to 1 wt%, and was approved by the Food and Drug Agency of the United States under Title 21 of the Code of Federal Regulations [338]. The additive contains TiO_2_ NPs as described by the E171, European Food Safety Authority (EFSA) of the European Union [339]. Titania is also widely used in toothpaste as a white pigment with a small fraction of TiO_2_ NPs. These nanoparticles are also detected in sweets containing E 171, e.g., chewing gum, colored candy, chocolate and cake-icing. The approximate oral ingestion of TiO_2_ NPs for the Dutch is 0.19 mg/kg bw/day for elderly, 0.55 mg/kg bw/day for 7–69 year old people, and 2.16 mg/kg bw/day for young children [340]. Very recently, Hwang et al. demonstrated that commercial TiO_2_ additive as outlined by the E171 contains particles with mean size values of 118–169 nm. The TiO_2_ additive created ROS and inhibited long-term colony formation in human intestinal epithelial Caco-2 cells at concentrations >125 µg/mL. The additive slightly induced apoptosis at a very high content of 1000 µg/mL upon exposure for 24 h [341]. This result raises a safety concern about the toxic impact of TiO_2_ food additives on human health [342]. Heringa et al. reported the presence of TiO_2_ NPs in 15 post-mortem human livers and spleens of Caucasians. Those human subjects followed a West European diet and used toothpaste, so this may have resulted from oral intake of TiO_2_ NPs [343].

Most in vitro cell cultivation and in vivo animal models tests clearly indicate that TiO_2_ NPs are toxic to mammalian cells, since they induce DNA damage, ROS generation, and apoptosis. In particular, Ag-doped TiO_2_ NPs act as a double-edged sword, having beneficial and adverse effects. The plasmonic effect of surface electrons of AgNP-decorated TiO_2_ NPs promotes light-harvesting in the visible region, as mentioned previously. This inhibits bacterial growth of *E. coli* and *S. aureus* effectively when compared to pure TiO_2_ NPs, as shown in Figure 20. Therefore, there exists a synergistic bactericidal effect of TiO_2_ and AgNPs through the photocatalytic reaction and Ag^0^/Ag^+^ species released from the AgNPs. However, metallic Ag^0^ and ionic Ag^+^ released from AgNPs can elicit a toxic effect on HepG2 cells through ROS generation (Figure 34). Furthermore, AgNPs have been found to induce a toxic effect on mammalian cells in a dose-, size- and time-dependent manner. In vivo animal studies reveal that AgNPs locate preferentially in murine target organs including the liver, spleen, kidney and brain after intratracheal instillation, intravenous or intraperitoneal injection [15].

**Figure 35:**
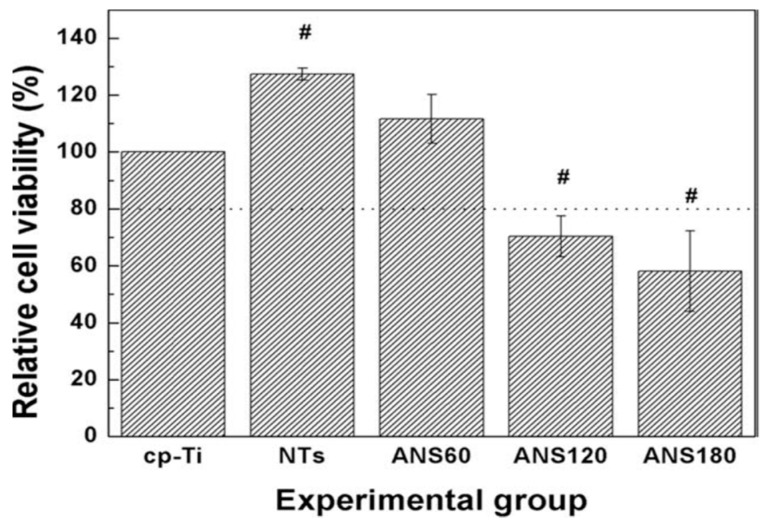
The viability of MC3T3-E1 murine osteoblasts obtained from the MTT assay. Error bars indicate the standard deviation (*n* = 5); # *p* < 0.05 compared with commercial pure Ti (cp-Ti). Reproduced with permission from [261]. Copyright Wiley, 2014.

Standalone and detached TNTs exhibit poor compatibility to human dermal fibroblasts due to ROS generation, leading to DNA damage and chromosomal aberration [308]. Ultrasonication was employed to detach TNTs from the Ti substrate. Standalone TNTs were able to pierce and penetrate through the membrane of fibroblasts, resulting in cytotoxicity. Without ultrasonication, intact TNTs adhered firmly on the Ti substrate, acting as the adhesion and growth sites for murine osteoblasts (MC3T3-E1) and human fibroblasts [226,261]. As such, anodic TNTs exhibited higher osteoblastic viability than pure Ti (Figure 35). After sputtered coating TNTs with silver for 60, 120 and 180 s (designated as ANS 60, ANS 120 and ANS 180), the ANS 60 sample still showed better biocompatibity than pure Ti. However, the cell viability of ANS 120 and ANS 180 samples decreased markedly because a longer sputtering time favored more AgNP formation on the nanotubes. Thus the ANS 180 sample exhibited the lowest cell proliferation. From Figure 21, ANS 60 showed a similar antibacterial actitvity to ANS 120 and ANS 180 samples. Therefore, a balance between bactericidal activity and cellular viability can be reached by monitoring the Ag content in ANS 60. In general, Ti-based alloy implants have inadequate antibacterial activity, so much effort has been made to improve their compatibility and antibacterial properties for clinical applications. Ti-based alloy implants with enhanced antibacterial activity and biocompatibility can be achieved by forming an Ag-doped TNTs surface layer with an optimal Ag content through anodization and sputtering. The modified TNTs formed on Ti-based implants can reduce bacterial colonization on their surfaces accordingly. Alternatively, antibacterial CuNPs can be used to replace AgNPs for doping TNTs with improved biocompatibility [98,99]. CuNPs are less toxic than AgNPs to human and bovine mammary epithelial cells [344].

However, it remains a big challenge for chemists and materials scientists to design novel nanomaterials for technological and medical applications utilizing TiO_2_ NPs and doped TiO_2_ NPs. With the increasing need for antibacterial scaffolds and wound dressings in tissue engineering, biodegradable polymers are ideal materials to immobilize TiO_2_ NPs to form polymer nanocomposites with improved biocompatibility and photocatalytic activity. It is of primary importance to select a proper polymeric material, suitable for those applications. As previously mentioned, mixing chitosan with TiO_2_ NPs can yield a biodegradable plastic film with visible light photocatalytic bactericidal activity. The film functions effectively for preserving fresh fruits and vegetables, and for prolonging shelf-life [289]. Such a plastic film can also be used as a food packaging material for killing pathogenic bacteria that causes food poisoning or food spoilage. In addition, biodegradable CS-TiO_2_ NPs film also finds potential applications as antibacterial scaffolds and wound dressings in orthopedics. By incorporating Cu dopant into TiO_2_ NPs, the resulting CS/Cu-doped TiO_2_ NPs shows higher photocatalytic bactericidal activity, as expected [288]. 

Finally, antibacterial and self-cleaning fabrics have received considerable attention in hospitals and clinics due to the increased risk of healthcare-associated infections. Those fabrics are particularly useful against nosocomial bacteria to protect patients from harmful microorganisms [298,345]. The fabrics made from cotton/Mn-doped TiO_2_ NPs have been reported to exhibit full inactivation of *S. aureus* and *K. pneumoniae* within 120 min under sunlight (Figure 32a,b) [298]. However, one can see that such fabrics need at least 25 wt% Mn-doped TiO_2_ NPs for achieving antibacterial properties. Those fillers can be removed from the fabrics upon several washing cycles, and then discharged into rivers and lakes. The recent literature data indicate the toxicity of rGO/TiO_2_ NPs in inducing teratogenic effects on zebrafish, although at doses considerably higher than those in aquatic environments [337]. The release of large amounts of Mn-doped TiO_2_ NPs would inevitably pollute the aquatic environment, and harm aquatic life in the ecosystem [346]. Moreover, Mn is a heavy metal that is toxic to mammalian cells. Manganese can induce toxicity in human bronchial epithelial cells, leading to caspase-9-mediated cell death [347,348]. Therefore, non-noble metal doped TiO_2_ NPs such as the N-doped TiO_2_ NPs photocatalyst is considere a potential replacement for Mn-doped TiO_2_ NPs for reducing environmental toxicity. 

Thanks to the efforts of researchers, visible-light active TiO_2_ NPs and their nanocomposites have been found to exhibit a bactericidal effect against drug-resistant bacteria including salmonella abony, *S. aureus*, *K. pneumoniae*, *P. aeruginosa* and anthrax. However, it still requires substantial time and effort to develop visible-light active TiO_2_ NPs photocatalysts with both antibacterial activity and good cytocompatibility. Long-term, in vivo animal studies relating to the biocompatibility and cytotoxicity of those photocatalysts are required. More research studies are needed to design the toxic free, green synthesis of visible-light active TiO_2_ NPs with bactericidal activity for industrial, environmental and medical applications. 

## 8. Conclusions

This review gives a summary of the synthesis, photocatalytic bacterial inactivation, biocompatibility and cytotoxic effects of visible-light active TiO_2_ NPs and their nanocomposites, especially over the past five years. The photocatalytic bactericidal effect of TiO_2_ NPs with a wide bandgap depends on the creation of an electron–hole pair under UV irradiation to generate ROS. The created radicals then interact with microorganisms, causing damage to the structure of the cell membrane and the subsequent leakage of intracellular components. However, UV-responsive TiO_2_ NPs exhibit poor photocatalytic efficiency under visible light. It is of practical importance to employ visible light to induce the photocatalytic bactericidal activity of TiO_2_ NPs. Considerable progress has recently been made in the development of TiO_2_ nanostuctures with good photocatalytic bactericidal activity under visible light irradiation. Visible-light active TiO_2_ nanomaterials can be synthetized through several techniques including sol-gel, hydrothermal/solvothermal, electrochemical anodization, and electrospinning. Among these, sol-gel is commonly used for preparing TiO_2_ NPs, while electrochemical anodization is an effective method for fabricating TNTs. 

Doping TiO_2_ NPs with metal and non-metal elements can lead to a red shift in the optical absorption edge into the visible region, resulting in a reduction of the bandgap accordingly. Transition metal elements, such as Mn, Fe, V and Cu, can create a localized d-electron state in the bandgap of TiO_2_ NPs, thereby promoting the separation of photogenerated electron–hole pair and suppressing the recombination of charge carriers. However, defect states in the bandgap of titania would also serve as charge recombination centers under experimental conditions of high dopant concentrations. Noble metal dopants such as AgNPs exhibit plasmonic oscillation of surface electrons under visible light irradiation. As such, hot-electron transfer from excited AgNPs into the conduction band of TiO_2_ NPs creates ROS for bactericidal activity. In addition to photocatalytic bactericidal activity, AgNPs of Ag-doped TiO_2_ NPs can inactivate bacteria through the release of Ag^+^ ions. These cations strongly interact with the thiol groups of microorganisms and inhibit DNA replication, resulting in apoptosis. Furthermore, AgNPs also induce a toxic effect on human cells. In this respect, we must be cautious when using AgNPs as a dopant for TiO_2_ NPs. Taking account of the adverse effects of metal dopants, visible-light active, anion-doped TiO_2_ NPs using non-metal elements have attracted increasing attention as agents against pathogenic bacteria. 

Given the important uses of visible-light active TiO_2_ NPs for various applications, their impact on human health poses a serious concern worldwide. In vitro cell cultivation and in vivo animal models studies have reported the cytotoxic effects of TiO_2_ NPs and Ag-doped TiO_2_ NPs in mammalian cells by inducing an inflammatory response, DNA damage, ROS generation, and apoptosis. For long-term safety, more research studies are needed to properly design a toxic free, green synthesis of visible-light active TiO_2_ NPs with bactericidal activity.

## Figures and Tables

**Table 1:** Visible-light active TiO_2_ NPs doped with metals and non-metals.

Dopants	New Band (Gap) State Created	Reference
Metals		
Ti	Ti^3+^, oxygen vacancy	[106,107]
Mn	Mn^2+^	[92]
Fe	Fe^3+^	[93,95]
Ni	Ni^2+^	[110]
Cu	Cu^2+^	[109]
V	V^4+^	[88]
Mo	Mo^6+^	[111,112]
Ce	Ce^3+^	[121,122]
Mo and W	Mo^6+^, W^6+^	[114]
V and Co	V^4+^, Co^2+^	[90]
Fe and Co	Fe^3+^, Co^2+^	[115]
		
Non-Metals		
N	N midgap	[166]
P	P^5+^	[173,174]
F	Ti^3+^, oxygen vacancy	[165,175]
F and N	Ti^3+^, oxygen vacancy, N midgap	[176]

**Table 2:** Bactericidal performance of modified titania nanoparticles under visible light.

Material	Size, nm	Bacteria	Complete Inactivation Time	Ref.
(1–3 mol%) Ni/TiO_2_ NPs	8–10	*E. coli*	>300 min (3% Ni dopant)	[248]
		*S. aureus*	>240 min (3% Ni dopant)	
		*Samonella abony*	>360 min (3% Ni dopant)	
(1–3 mol%) Cu/TiO_2_ NPs	9–10	*E. coli*	240 min (3% Cu dopant)	[247]
		*S. aureus*	120 min (3% Cu dopant)	
0.5%Cu/TiO_2_ NPs	28.84	*E. coli*	30 min	[109]
		*S. aureus*	30 min	
Ag/TiO_2_ NPs	AgNPs: 0.9; TiO_2_ NPs: 8	*E. coli*	60 min	[124]
N/TiO_2_ NPs	10–30	*E. coli*	420 min	[161]
		*S. aureus*	360 min	
F-N doped P25	70	*E. coli*	60 min	[270]
F-N doped TiO_2_	21.3	*E. coli*	60 min	[271]
(0.5–2.5%) rGO/TiO_2_ NPs	17–18	*E. coli*	75 min (1.5% rGO/TiO_2_ NPs)	[101]
(0.1–0.5%) MWNT/TiO_2_ NPs	TiO_2_ NPs: 8–15 MWNT diameter: 20–45	*E. coli*	300 min (0.5% MWNT/TiO_2_)	[156]
		*S. aureus*	180 min (0.5% MWNT/TiO_2_)	
0.5 wt% MWNT/Fe-doped TiO_2_	Fe-doped TiO_2_: 15–20 MWNT diameter: 20–45	*B. subtilis*	120 min	[157]
		*P. aeruginosa*	240 min	
Cu_2_O/TiO_2_	TiO_2_ NPs: 8	*E. coli*	60 min	[177]
CS/Cu-doped TiO_2_	16	*E. coli*	120 min	[288]
Cotton/(10–50%) Mn-doped TiO_2_	Mn-doped TiO_2_: 150	*S. aureus*	90 min (25 wt% Mn dopant)	[298]
		*K. pneumoniae*	90 min (25 wt% Mn dopant)	
Cotton/(10–50%) Mn-doped TiO_2_	Mn-doped TiO_2_: 150	*S. aureus*	60 min (50 wt% Mn dopant)	[298]
		*K. pneumoniae*	120 min (50 wt% Mn dopant)	

## References

[1] Economou V., Gousia P. (2015). Agriculture and food animals as a source of antimicrobial-resistant bacteria. Infect. Drug Resist..

[2] Rousham E.K., Unicomb L., Aminul M. (2018). Human, animal and environmental contributors to antibiotic resistance in low-resource settings: Integrating behavioural, epidemiological and one health approaches. Proc. R. Soc. B Biol. Sci..

[3] Li B., Webster T.J. (2018). Bacteria antibiotic resistance: New challenges and opportunities for implant-associated orthopedic infections. J. Orthop. Res..

[4] Kavanagh N., Ryan E.J., Widaa A., Sexton G., Fennell J., O’Rourke S., Cahill K.C., Kearney C.J., O’Brien F.J., Kerrigan S.W. (2018). Staphylococcal osteomyelitis: Disease progression, treatment challenges, and future directions. Clin. Microbiol. Rev..

[5] Collignon P.J., McEwen S.A. (2019). One health—Its importance in helping to better control antimicrobial resistance. Trop. Med. Infect. Dis..

[6] Huh A.J., Kwon Y.J. (2011). Nanoantibiotics: A new paradigm for treating infectious diseases using nanomaterials in the antibiotics resistant era. J. Control. Release.

[7] Shaikh S., Nazam N., Rizvi S.M., Ahmad K., Baig M.H., Lee E.J., Choi I. (2019). Mechanistic insights into the antimicrobial actions of metallic nanoparticles and their implications for multidrug resistance. Int. J. Mol. Sci..

[8] Jesline A., John N.P., Narayanan P.M., Vani C., Murugan S. (2015). Antimicrobial activity of zinc and titanium dioxide nanoparticles against biofilm-producing methicillin-resistant *Staphylococcus aureus*. Appl. Nanosci..

[9] Chen C., Li S., Thomas A., Kotov N.A., Haag R. (2017). Functional graphene nanomaterials based architectures: Biointeractions, fabrications, and emerging biological applications. Chem. Rev..

[10] Tjong S.C., Chen H. (2004). Nanocrystalline materials and coatings. Mater. Sci. Eng. R Rep..

[11] Tjong S.C. (2013). Nanocrystalline Materials: Their Synthesis-Structure-Property Relationships and Applications.

[12] He L.X., Tjong S.C. (2016). Nanostructured transparent conductive films: Fabrication, characterization and applications. Mater. Sci. Eng. R Rep..

[13] He L.X., Tjong S.C. (2016). Aqueous graphene oxide-dispersed carbon nanotubes as inks for the scalable production of all-carbon transparent conductive films. J. Mater. Chem. C.

[14] Liao C., Li Y., Tjong S.C. (2018). Graphene nanomaterials: Synthesis, biocompatibility, and cytotoxicity. Int. J. Mol. Sci..

[15] Liao C., Li Y., Tjong S.C. (2019). Bactericidal and cytotoxic properties of silver nanoparticles. Int. J. Mol. Sci..

[16] He L., Liao C., Tjong S.C. (2018). Scalable fabrication of high-performance transparent conductors using graphene oxide-stabilized single-walled carbon nanotube inks. Nanomaterials.

[17] Graves J.L., Thomas M., Ewunkem J.A. (2017). Antimicrobial nanomaterials: Why evolution matters. Nanomaterials.

[18] Gupta A., Mumtaz S., Li C.H., Hussain I., Rotello V.M. (2019). Combatting antibiotic-resistant bacteria using nanomaterials. Chem. Soc. Rev..

[19] Küünal S., Visnapuu M., Volubujeva O., Soares Rosario M., Rauwel P., Rauwel E. (2019). Optimisation of plant mediated synthesis of silver nanoparticles by common weed Plantago major and their antimicrobial properties. IOP Conf. Ser. Mater. Sci. Eng..

[20] Küünal S., Rauwel P., Rauwel E., Barhoum A., Makhlouf A.S. (2018). Plant extract mediated synthesis of nanoparticles. Emerging Applications of Nanoparticles and Architectural Nanostructures: Current Prospects and Future Trends.

[21] Collin F. (2019). Chemical basis of reactive oxygen species reactivity and involvement in neurodegenerative diseases. Int. J. Mol. Sci..

[22] Regmi C., Joshi B., Ray S.K., Gyawali G., Pandey R.P. (2018). Understanding mechanism of photocatalytic microbial decontamination of environmental wastewater. Front. Chem..

[23] Ballottin D., Fulaz S., Cabrini F., Tsukamoto J., Duran N., Alves O.L., Tasic L. (2017). Antimicrobial textiles: Biogenic silver nanoparticles against Candida and Xanthomonas. Mater. Sci. Eng. C.

[24] Chernousova S., Epple M. (2013). Silver as antibacterial agent: Ion, nanoparticle and metal. Angew. Chem. Int. Ed..

[25] Kedziora A., Speruda M., Krzyzewska E., Rybka J., LukowiK A., Bugla-Płoskonska G. (2018). Similarities and differences between silver ions and silver in nanoforms as antibacterial agents. Int. J. Mol. Sci..

[26] Liu C., Shen J., Yeung K.W.K., Tjong S.C. (2017). Development and antibacterial performance of novel polylactic acid-graphene oxide-silver nanoparticle hybrid nanocomposite mats prepared by electrospinning. ACS Biomater. Sci. Eng..

[27] Liu C., Shen J., Liao C.Z., Yeung K.W., Tjong S.C. (2018). Novel electrospun polyvinylidene fluoride-graphene oxide-silver nanocomposite membranes with protein and bacterial antifouling characteristics. Express Polym. Lett..

[28] Hanaor D.A., Sorrell C.C. (2011). Review of the anatase to rutile phase transformation. J. Mater. Sci..

[29] Sarkar A., Khan G.G. (2019). The formation and detection techniques of oxygen vacancies in titanium oxide-based nanostructures. Nanoscale.

[30] Pesci F.M., Wang G., Klug D.R., Li Y., Cowan A.J. (2013). Efficient suppression of electron-hole recombination in oxygen-deficient hydrogen-treated TiO_2_ nanowires for photoelectrochemical water splitting. J. Phys. Chem. C.

[31] Zhao H., Pan F., Li Y. (2017). A review on the effects of TiO_2_ surface point defects on CO_2_ photoreduction with H_2_O. J. Mater..

[32] Shwetharani R., Sakar M., Fernando C.A., Binas V., Balakrishna R.G. (2019). Recent advances and strategies to tailor the energy levels, active sites and electron mobility in titania and its doped/composite analogues for hydrogen evolution in sunlight. Catal. Sci. Technol..

[33] Rauwel E., Galeckas A., Rauwel P. (2014). Photoluminescent cubic and monoclinic HfO_2_ nanoparticles: Effects of temperature and ambient. Mater. Res. Express.

[34] Wang B., Huang W., Chi L., Al-Hashimi M., Marks T.J., Facchetti A. (2018). High-k gate dielectrics for emerging flexible and stretchable electronics. Chem. Rev..

[35] Markov S.L., Vidaković A.M. (2014). Testing methods for antimicrobial activity of TiO_2_ photocatalyst. Acta Period Technol..

[36] Rajh T., Dimitrijevic N.M., Bissonnette M., Koritarov T., Konda V. (2014). Titanium dioxide in the service of the biomedical revolution. Chem. Rev..

[37] Matsunaga T.R., Tomoda Y., Nakajima T., Wake H. (1985). Photoelectrochemical sterilization of microbial cells by semiconductor powders. FEMS Microbiol. Lett..

[38] Bogdan J., Zarzynska J., Plawinska-Czarnak J. (2015). Comparison of infectious agents susceptibility to photocatalytic effects of nanosized titanium and zinc oxides: A practical approach. Nanoscale Res. Lett..

[39] Wang Y., Wu T., Zhou Y., Meng C., Zhu W., Liu L. (2017). TiO_2_-based nanoheterostructures for promoting gas sensitivity performance: Designs, developments, and prospects. Sensors.

[40] Abbas M., Iftikhar H., Malik M.H., Nazir A. (2018). Surface coatings of TiO_2_ nanoparticles onto the designed fabrics for enhanced self-cleaning properties. Coatings.

[41] Huang Y., Mei L., Chen X., Wang Q. (2018). Recent developments in food packaging based on nanomaterials. Nanomaterials.

[42] Haggerty J.E.S., Schelhas L.T., Kitchaev D.A., Mangum J.S., Garten L.M., Sun W., Stone K.H., Perkins J.D., Toney M.F., Ceder G. (2017). High-fraction brookite films from amorphous precursors. Sci. Rep..

[43] Diebold U. (2003). The surface sicence of titanium dioxide. Surf. Sci. Rep..

[44] Lin X., Li J., Ma S., Liu G., Yang K., Tong M., Lin D. (2014). Toxicity of TiO_2_ nanoparticles to Escherichia coli: Effects of particle size, crystal phase and water chemistry. PLoS ONE.

[45] Maziarz W., Kursior A., Trenczek-Zajac A. (2016). Nanostructured TiO_2_-based gas sensors with enhanced sensitivity to reducing gases. Beilstein J. Nanotechnol..

[46] Raghu A.V., Karuppanan K.K., Nampoothin J., Pullithadathil B. (2019). Wearable, flexible ethanol gas sensor based on TiO_2_ nanoparticles-grafted 2D-titanium carbide nanosheets. ACS Appl. Nano Mater..

[47] Liu G., Yang H.G., Pan J., Yang Y.Q., Lu G.Q., Cheng H.M. (2014). Titanium dioxide crystals with tailored facets. Chem. Rev..

[48] Al-Attafi K., Nattestad A., Wu Q., Ide Y., Yamauchi Y., Dou S.X., Kim J.H. (2018). The effect of amorphous TiO_2_ in P25 on dye-sensitized solar cell performance. Chem. Commun..

[49] Truppi A., Petronella F., Placido T., Striccoli M. (2018). Visible-light-active TiO_2_-based hybrid nanocatalysts for environmental applications. Catalysis.

[50] Tobaldi D.M., Piccirillo C., Pullar R.C., Gualtieri A.F., Seabra M.P., Castro P.M., Labrincha J.A. (2014). Silver-modified nano-titania as an antibacterial agent and photocatalyst. J. Phys. Chem. C.

[51] Ali T., Ahmed A., Alam U., Uddin I., Tripathi P., Muneer M. (2018). Enhanced photocatalytic and antibacterial activities of Ag-doped TiO_2_ nanoparticles under visible light. Mater. Chem. Phys..

[52] Moongraksathum B., Chen Y.W. (2018). Anatase TiO_2_ co-doped with silver and ceria for antibacterial application. Catal. Today.

[53] Viet P.V., Phan B.T., Mott D., Maenosono S., Sang T.T., Thi C.M., Hieu L.V. (2018). Silver nanoparticle loaded TiO_2_ nanotubes with high photocatalytic and antibacterial activity synthesized by photoreduction method. J. Photochem. Photobiol. A.

[54] Ahmad R., Mohsin M., Ahmad D., Sardar M. (2015). Alpha amylase assisted synthesis of TiO_2_ nanoparticles: Structural characterization and application as antibacterial agents. J. Hazard. Mater..

[55] Lorenzetti M., Gongadze E., Kulkarni M., Junkar I., Iglic A. (2016). Electrokinetic properties of TiO_2_ nanotubular surfaces. Nanoscale Res. Lett..

[56] Jiang X., Lv B., Wang Y., Shen Q., Wang X. (2017). Bactericidal mechanisms and effector targets of TiO_2_ and Ag-TiO_2_ against Staphylococcus aureus. J. Med. Microbiol..

[57] Pagnout C., Jomini S., Dadhwal M., Caillet C., Thomas F., Bauda P. (2012). Role of electrostatic interactions in the toxicity of titanium dioxide nanoparticles toward Escherichia coli. Colloids Surf. B Biointerfaces.

[58] Liou J.W., Chang H.H. (2012). Bactericidal effects and mechanisms of visible light responsive titanium dioxide photocatalysts on pathogenic bacteria. Arch. Immunol. Ther. Exp..

[59] Charpentier P.A., Burgess K., Wang L., Chowdhury R.R., Lotus A.F., Moula G. (2012). Nano-TiO_2_/polyurethane composites for antibacterial and self-cleaning coatings. Nanotechnology.

[60] Santhosh S.M., Kandasamy N. (2015). Antibiofilm activity of epoxy/Ag-TiO_2_ polymer nanocomposite coatings against *Staphylococcus aureus* and *Escherichia coli*. Coatings.

[61] Khorshidi B., Biswas I., Ghosh T., Thundat T., Sadrzadeh M. (2018). Polyamide-TiO_2_ nanocomposite membranes with enhanced thermal stability and anti-biofouling propensity. Sci. Rep..

[62] Zhang S., Liang X., Gadd G.M., Zhao Q. (2019). Advanced titanium dioxide-polytetrafluorethylene (TiO_2_-PTFE) nanocomposite coatings on stainless steel surfaces with antibacterial and anti-corrosion properties. Appl. Surf. Sci..

[63] Ahmadi R., Tanomand A., Kazeminawa F., Kamounah F.S., Ayaseh A., Ganbarov K., Yousefi M., Katourani A., Yousefi B., Kafil H.S. (2019). Fabrication and characterization of a titanium dioxide (TiO_2_) reinforced with bio-nanocomposite containing Miswak (*Salvadora percica* L.) extract–the antimicrobial, thermophysical, and barrier properties. Int J. Nanomedic..

[64] Hegedus P., Szabó-Bárdos E., Horvath O., Szabo P., Horvath K. (2017). Investigation of a TiO_2_ photocatalyst immobilized with poly(vinyl alcohol). Catal. Today.

[65] Bui V.K., Park D., Lee Y.C. (2017). Chitosan combined with ZnO, TiO_2_ and Ag nanoparticles for antimicrobial wound healing applications: A mini review of the research trends. Polymers.

[66] Meng Y.Z., Tjong S.C. (1998). Rheology and morphology of compatibilized polyamide 6 blends containing liquid crystalline copolyesters. Polymer.

[67] Meng Y.Z., Tjong S.C., Hay A.S., Wang S.J. (2001). Synthesis and proton conductivities of phosphonic acid containing poly-(arylene ether)s. J. Polym. Sci. A Polym. Chem..

[68] Liu C., Chan K.W., Shen J., Liao C., Yeung K.W.K., Tjong S.C. (2016). Polyetheretherketone hybrid composites with bioactive nanohydroxyapatite and multiwalled carbon nanotube fillers. Polymers.

[69] Chan K.W., Liao C., Wong H.M., Yeung K.W.K., Tjong S.C. (2016). Preparation of polyetheretherketone composites with nanohydroxyapatite rods and carbon nanofibers having high strength, good biocompatibility and excellent thermal stability. RSC Adv..

[70] Liao C., Li K., Wong H.M., Tong W.Y., Yeung K.W.K., Tjong S.C. (2013). Novel polypropylene biocomposites reinforced with carbon nanotubes and hydroxyapatite nanorods for bone replacements. Mater. Sci. Eng. C.

[71] Liao C., Wong H.M., Yeung K.W.K., Tjong S.C. (2014). The development, fabrication and material characterization of polypropylene composites reinforced with carbon nanofiber and hydroxyapatite nanorod hybrid fillers. Int. J. Nanomed..

[72] Liu C., Wong H.M., Yeung K.W., Tjong S.C. (2016). Novel electrospun polylactic acid nanocomposite fiber mats with hybrid graphene oxide and nanohydroxyapatite reinforcements having enhanced biocompatibility. Polymers.

[73] Horn M., Schwerdtfeger C.F., Meagher E.P. (1972). Refinement of the structure of anatase at several temperatures. Z. Krist..

[74] Baur W.H., Khan A.A. (1971). Rutile-type compounds. IV. SiO_2_, GeO_2_ and a comparison with other rutile-type structures. Acta Crystallogr. B.

[75] Fagan R., Synnott D.W., McCormack D.E., Pillai S.C. (2016). An effective method for the preparation of high temperature stable anatase TiO_2_ photocatalysts. Appl. Surf. Sci..

[76] Higashimoto S. (2019). Titanium-dioxide-based visible-light-sensitive photocatalysis: Mechanistic insight and applications. Catalysts.

[77] Lu Y., Zang Y., Zhang H., Zhang Y., Wang G., Zhao H. (2016). Meaningful comparison of photocatalytic properties of {001} and {101} faceted anatase TiO_2_ nanocrystals. Sci. Bull..

[78] Sajan C.P., Wageh S., Al-Ghamdhi A.A., Yu J., Cao S. (2016). TiO_2_ nanosheets with exposed {001} facets for photocatalytic application. Nano Res..

[79] Liu X., Du G., Li M. (2019). True photoreactivity origin of Ti^3+^-doped anatase TiO_2_ crystals with respectively dominated exposed {001}, {101}, and {100} facets. ACS Omega.

[80] Li M., Yin J.J., Wamer W.G., Lo Y.M. (2014). Mechanistic characterization of titanium dioxide nanoparticle-induced toxicity using electron spin resonance. J. Food Drug Anal..

[81] Xue C., Wu J., Lan F., Liu W., Yang X., Zeng F., Xu H. (2010). Nano titanium dioxide induces the generation of ROS and potential damage in HaCaT cells under UVA irradiation. J. Nanosci. Nanotechnol..

[82] Bartlet K., Movafaghi S., Dasi L.P., Kota A.K., Popat K.C. (2018). Antibacterial activity on superhydrophobic titania nanotube arrays. Colloids Surf. B Biointerfaces.

[83] Dong H., Zeng G., Tang L., Fan C., Zhang C., He X. (2015). An overview on limitations of TiO_2_-based particles for photocatalytic degradation of organic pollutants and the corresponding countermeasures. Water Res..

[84] Pelaez M., Nolan N., Pillai S., Seery M., Falaras P., Kontos A.G., Dunlop P.S., Hamilton J.W., Byrne J.A., O’Shea K. (2012). A review on the visible light active titanium dioxide photocatalysts for environmental applications. Appl. Catal. B Environ..

[85] Schneider J., Matsuoka M., Takeuchi M., Zhang J., Horiuchi Y., Anpo M., Bahnemann D.W. (2014). Understanding TiO_2_ photocatalysis: Mechanisms and materials. Chem. Rev..

[86] Moma J., Baloyi J., Khan S.B., Akhtar K. (2019). Modified Titanium Dioxide for Photocatalytic Applications. Photocatalysts—Applications and Attributes.

[87] Kang X., Liu S., Dai Z., He Y., Song X., Tang Z. (2019). Titanium dioxide: From engineering to applications. Catalysis.

[88] Lin W.C., Lin Y.J. (2012). Effect of vanadium (IV)-doping on the visible light-induced catalytic activity of titanium dioxide catalysts for methylene blue degradation. Environ. Eng. Sci..

[89] Yu J.H., Nam S.H., Lee J.W., Kim D.I., Boo J.H. (2019). Oxidation state and structural studies of vanadium-doped titania particles for the visible light-driven photocatalytic activity. Appl. Surf. Sci..

[90] Lv T., Zhao J., Chen M., Shen K., Zhang D., Zhang J., Zhang G., Liu Q. (2018). Boosted visible-light photodegradation of methylene blue by V and Co co-doped TiO_2_. Materials.

[91] Khatun N., Rini E.G., Shirage P., Rajput P., Jha S.N., Sen S. (2016). Effect of lattice distortion on bandgap decrement due to vanadium substitution in TiO_2_ nanoparticles. Mater. Sci. Semicond. Process..

[92] Binas V., Venieri D., Kotzias D., Kiriakidis D. (2017). Modified TiO_2_ based photocatalysts for improved air and health quality. J. Mater..

[93] Moradi H., Eshaghi A., Hosseini S.R., Ghani K. (2016). Fabrication of Fe-doped TiO_2_ nanoparticles and investigation of photocatalytic decolorization of reactive red 198 under visible light irradiation. Ultrason. Sonochem..

[94] Crisan M., Mardare D., Ianculescu A., Dragan N., Nitoi I., Crisan D., Voicescu M., Todan L., Oancea P., Adomnitei C. (2018). Iron doped TiO_2_ films and their photoactivity in nitrobenzene removal from water. Appl. Surf. Sci..

[95] Daghrir R., Drogui P., Robert D. (2013). Modified TiO_2_ for environmental photocatalytic applications: A review. Ind. Eng. Chem. Res..

[96] Li W. (2014). Influence of electronic structures of doped TiO_2_ on their photocatalysis. Phys. Status Solidi Rapid Res. Lett..

[97] Ivanov S., Barylyak A., Besaha K., Bund A., Wojnarowska-Nowak R., Yaremchuk I., Kus-Liśkiewicz M. (2016). Synthesis, characterization, and photocatalytic properties of sulfur- and carbon-codoped TiO_2_ nanoparticles. Nanoscale Res. Lett..

[98] Koklic T., Pintaric S., Zdovc I., Golob M., Umek P., Mehle A., Dobeic M., Strancar J. (2018). Photocatalytic disinfection of surfaces with copper doped TiO_2_ nanotube coatings illuminated by ceiling mounted fluorescent light. PLoS ONE.

[99] Syrek K., Grudzień J., Sennik-Kubiec A., Brudzisz A., Sulka G.D. (2019). Anodic titanium oxide layers modified with gold, silver, and copper nanoparticles. J. Nanomater..

[100] Janczarek M., Wei Z., Endo M., Ohtani B., Kowalska E. (2016). Silver- and copper modified decahedral anatase titania particles as visible light-responsive plasmonic photocatalyst. J. Photon. Energy.

[101] Wanag A., Rokicka P., Kusiak-Nejman E., Kapica-Kozar J., Wrobel R.J., Markowska-Szczupak A., Morawski A.W. (2018). Antibacterial properties of TiO_2_ modified with reduced graphene oxide. Ecotoxicol. Environ. Saf..

[102] Raja A., Selvakumar K., Rajasekaran P., Arunpandian M., Ashokkumar S., Kaviyarasu K., Asath Bahadur S., Swaminathan M. (2019). Visible active reduced graphene oxide loaded titania for photodecomposition of ciprofloxacin and its antibacterial activity. Colloids Surf. A.

[103] Tayel A., Ramadan A.R., El Seoud O.A. (2018). Titanium dioxide/graphene and titanium dioxide/graphene oxide nanocomposites: Synthesis, characterization and photocatalytic applications for water decontamination. Catalysts.

[104] Rauwel P., Galeckas A., Salumaa M., Ducroquet F., Rauwel E. (2016). Photocurrent generation in carbon nanotube/cubic-phase HfO_2_ nanoparticle hybrid nanocomposites. Beilstein J. Nanotechnol..

[105] Choi W., Termin A., Hoffmann M.R. (1994). The role of metal ion dopants in quantum-sized TiO_2_: Correlation between photoreactivity and charge carrier recombination dynamics. J. Phys. Chem..

[106] Fang W.Z., Xing M.Y., Zhang J.L. (2014). A new approach to prepare Ti^3+^ self-doped TiO_2_ via NaBH_4_ reduction and hydrochloric acid treatment. Appl. Catal. B Environ..

[107] Wan Z., Huang G.F., Huang W.Q., Jiao C., Yan X.G., Yang Z.M., Zhang Q.L. (2014). The enhanced photocatalytic activity of Ti^3+^ self-doped TiO_2_ by a reduction method. Mater. Lett..

[108] Jayashree S., Ashokkumar M. (2018). Switchable intrinsic defect chemistry of titania for catalytic applications. Catalysts.

[109] Mathew S., Ganguly P., Rhatigan S., Kumaravel V., Byrne C., Hinder S.J., Bartlett J., Nolan M., Pillai S.C. (2018). Cu-doped TiO_2_: Visible light assisted photocatalytic antimicrobial activity. Appl. Sci..

[110] Manzoor M., Rafiq A., Ikram M., Nafees M., Ali S. (2018). Structural, optical, and magnetic study of Ni-doped TiO_2_ nanoparticles synthesized by sol–gel method. Int. Nano Lett..

[111] Huang J.G., Guo X.T., Wang B., Li L.Y., Zhao M.X., Dong L.L., Liu X.J., Huang Y.T. (2015). Synthesis and photocatalytic activity of Mo-doped TiO_2_ nanoparticles. J. Spectrosc..

[112] Avilés-García O., Espino-Valencia J., Romero R., Rico-Cerda J.L., Arroyo-Albiter M., Natividad R. (2017). W and Mo doped TiO_2_: Synthesis, characterization and photocatalytic activity. Fuel.

[113] Shi Z., Lai H., Yao S., Wang S. (2012). Photocatalytic activity of Fe and Ce co-doped mesoporous TiO_2_ catalyst under UV and visible light. J. Chin. Chem. Soc..

[114] Aviles-Garcia O., Espino-Valencia J., Romero-Romero R., Rico-Cerda J.L., Arroyo-Albiter M., Solis-Casados D.A., Navitidad-Rangel R. (2018). Enhanced photocatalytic activity of titania by co-doping with Mo and W. Catalysts.

[115] El Mragui A., Logvina Y., Pinto da Silva L., Zegaoui O., Estevesda Silva J.C.G. (2019). Synthesis of Fe- and Co-doped TiO_2_ with improved photocatalytic activity under visible irradiation toward carbamazepine degradation. Materials.

[116] Nadolna J., Grzyb G., Sobczak J.W., Lisowski W. (2015). Visible light activity of rare earth metal doped (Er^3+^, Yb^3+^ or Er^3+^/Yb^3+^) titania photocatalysts. Appl. Catal. B Envorin..

[117] Rozman N., Tobaldi D.M., Cvelbar U., Puliyalil H., Labrincha J.A., Legat A., Skapin A.S. (2019). Hydrothermal synthesis of rare-earth modified titania: Influence on phase composition, optical properties, and photocatalytic activity. Materials.

[118] Mazierski P., Lisowski W., Grzyb T., Winiarski M.J., Klimczuk T., Mikołajczyk A., Flisikowski J., Hirsch A., Kolakowska A., Puzyn T. (2017). Enhanced photocatalytic properties of lanthanide-TiO_2_ nanotubes: An experimental and theoretical study. Appl. Catal. B Environ..

[119] Xie K., Jia Q., Wang Y., Zhang W., Xu J. (2018). The electronic structure and optical properties of anatase TiO_2_ with rare earth metal dopants from first-principles calculations. Materials.

[120] Makdee A., Unwiset P., Chanapattharapol K.C., Kidkhunthod P. (2018). Effects of Ce addition on the properties and photocatalytic activity of TiO_2,_ investigated by X-ray absorption spectroscopy. Mater. Chem. Phys..

[121] Kasinathan K., Kennedy J., Elayaperumal M., Henini M., Malik M. (2016). Photodegradation of organic pollutants RhB dye using UV simulated sunlight on ceria based TiO_2_ nanomaterials for antibacterial applications. Sci. Rep..

[122] Choudhury B., Borah B., Choudhury A. (2012). Extending photocatalytic activity of TiO_2_ nanoparticles to visible region of illumination by doping of cerium. Photochem. Photobiol..

[123] Li J., Zhou H., Qian S., Liu Z., Feng J., Jin P., Liu X. (2014). Plasmonic gold nanoparticles modified titania nanotubes for antibacterial application. Appl. Phys. Lett..

[124] Endo M., Wei Z., Wang K., Karabiyik B., Yoshiiri K., Rokickka P., Ohtani B., Markowska-Szczupak A., Kowalska E. (2018). Noble metal-modified titania with visible-light activity for the decomposition of microorganisms. Beilstein J. Nanotechnol..

[125] Wysocka I., Kowalska E., Ryl J., Nowaczyk G., Zielińska-Jurek A. (2019). Morphology, photocatalytic and antimicrobial properties of TiO_2_ modified with mono- and bimetallic copper, platinum and silver nanoparticles. Nanomaterials.

[126] Petica A., Florea A., Gaidau C., Balan D., Anicai L. (2019). Synthesis and characterization of silver-titania nanocomposites prepared by electrochemical method with enhanced photocatalytic characteristics, antifungal and antimicrobial activity. J. Mater. Res. Technol..

[127] Krajczewski J., Kolataj K., Kudelski A. (2017). Plasmonic nanoparticles in chemical analysis. RSC Adv..

[128] Kim M., Lin M., Son J., Xu H., Nam J.M. (2017). Hot-electron-mediated photochemical reactions: Principles, recent advances, and challenges. Adv. Opt. Mater..

[129] Furube A., Hashimoto S. (2017). Insight into plasmonic hot-electron transfer and plasmon molecular drive: New dimensions in energy conversion and nanofabrication. NPG Asia Mater..

[130] Zhang Z., Zhang C., Zheng H., Xu H. (2019). Plasmon-driven catalysis on molecules and nanomaterials. Acc. Chem. Res..

[131] Hartland G.V., Besteiro L.V., Johns P., Govorov A.O. (2017). What’s so hot about electrons in metal nanoparticles?. ACS Energy Lett..

[132] Kim J., Son H.Y., Nam Y.S. (2018). Multilayered plasmonic heterostructure of gold and titania nanoparticles for solar fuel production. Sci. Rep..

[133] Wu N. (2018). Plasmonic metal–semiconductor photocatalysts and photoelectrochemical cells: A review. Nanoscale.

[134] Clavero C. (2014). Plasmon-induced hot-electron generation at nanoparticle/metal-oxide interfaces for photovoltaic and photocatalytic devices. Nat. Photonics.

[135] Khan M.R., Chowdhurya M.N., Chuan T.W., Cheng C.K. (2015). Schottky barrier and surface plasmonic resonance phenomena towards the photocatalytic reaction: Study of their mechanisms to enhance the photocatalytic activity. Catal. Sci. Technol..

[136] Yao G.Y., Liu Q.L., Zhao Z.Y. (2018). Studied localized surface plasmon resonance effects of Au nanoparticles on TiO_2_ by FDTD simulations. Catalysts.

[137] Michalas L., Khiat A., Stathopoulos S., Prodromakis T. (2018). Electrical characteristics of interfacial barriers at metal—TiO_2_ contacts. J. Phys. D Appl. Phys..

[138] Hankodo C.T., Moustakas N.G., Peppel T., Springer A., Oropeza F.E., Huda A., Bustan M.D., Yudono B., Gulo F., Strunk J. (2019). Characterization and effect of Ag(0) vs. Ag(I) species and their localized plasmon resonance on photochemically inactive TiO_2_. Catalysts.

[139] Ma Y., Zhi L. (2019). Graphene-based transparent conductive films: Material systems, preparation and applications. Small Methods.

[140] Nair R.R., Blake P., Grigorenko A.N., Novoselov K.S., Booth T.J., Stauber T., Peres N.M., Geim A.K. (2008). Fines structure constant defines visual transparency of graphene. Science.

[141] Kim C.H. (2018). Nanostructured graphene: An active component in optoelectronic devices. Nanomaterials.

[142] Kumar P., Huo P., Zhang R., Liu B. (2019). Antibacterial properties of graphene-based nanomaterials. Nanomaterials.

[143] Karahan H.E., Wiraja C., Xu C., Wei J., Wang Y., Wang L., Liu F., Chen Y. (2018). Graphene materials in antimicrobial nanomedicine: Current status and future perspectives. Adv. Healthc. Mater..

[144] Gillespie N.O., Martsinovich N. (2019). Origin of charge trapping in TiO_2_/reduced graphene oxide photocatalytic composites: Insights from theory. ACS Appl. Mater. Interf..

[145] Tang B., Chen H., Peng H., Wang Z., Huang W. (2018). Graphene modified TiO_2_ composite photocatalysts: Mechanism, progress and perspective. Nanomaterials.

[146] Tan L.L., Ong W.J., Chai S.P., Mohamed A.R. (2013). Reduced graphene oxide-TiO_2_ nanocomposite as a promising visible-light-active photocatalyst for the conversion of carbon dioxide. Nanoscale Res. Lett..

[147] Polat E.O., Balci O., Kakenov N., Uzlu H.B., Kocabas C., Dahiya R. (2015). Synthesis of large area graphene for high performance in flexible optoelectronic devices. Sci. Rep..

[148] Guerrero-Contreras J., Caballero-Briones F. (2015). Graphene oxide powders with different oxidation degree, prepared by synthesis variations of the Hummers method. Mater. Chem. Phys..

[149] Dreyer D.R., Park S., Bielawski C.W., Ruoff R.S. (2010). The chemistry of graphene oxide. Chem. Soc. Rev..

[150] Park S., An J., Potts J.R., Velamakanni A., Murali S., Ruoff R.S. (2011). Hydrazine-reduction of graphite- and graphene oxide. Carbon.

[151] Xu C., Shi X., Ji A., Shi L., Zhou C., Cui Y. (2015). Fabrication and characteristics of reduced graphene oxide produced with different green reductants. PLoS ONE.

[152] Yeh T.F., Teng T.C., Chen L.C., Teng H. (2016). Graphene oxide-based nanomaterials for efficient photoenergy conversion. J. Mater. Chem. A.

[153] Giovannetti R., Rommozzi E., Zannotti M., D’Amato C.A. (2017). Recent advances in graphene based TiO_2_ nanocomposites (GTiO_2_Ns) for photocatalytic degradation of synthetic dyes. Catalysts.

[154] Rauwel P., Galeckas A., Ducroquet F., Rauwel E. (2019). Selective photocurrent generation in HfO_2_ and carbon nanotube hybrid nanocomposites under Ultra-Violet and visible photoexcitations. Mater. Lett..

[155] Akhavan O., Azimirad R., Safa S., Larijani M.M. (2010). Visible light photo-induced antibacterial activity of CNT–doped TiO_2_ thin films with various CNT contents. J. Mater. Chem..

[156] Koli V.B., Dhodamani A.G., Raut A.V., Thorat N.D., Pawar S.H., Delekar S.D. (2016). Visible light photo-induced antibacterial activity of TiO_2_-MWCNTs nanocomposites with varying the contents of MWCNTs. J. Photochem. Photobiol. A.

[157] Koli V.B., Delekar S.D., Pawar S.H. (2016). Photoinactivation of bacteria by using Fe-doped TiO_2_-MWCNTs nanocomposites. J. Mater. Sci. Mater. Med..

[158] Choi J., Park H., Hoffmann M.R. (2010). Effects of single metal-ion doping on the visible-light photoreactivity of TiO_2_. J. Phys. Chem. C.

[159] Nagpure S., Kim D.Y., Rankin S.E. (2017). Synthesis and catalytic applications of non-metal doped mesoporous titania. Inorganics.

[160] Valentin C.D., Pacchioni G. (2013). Trends in non-metal doping of anatase TiO_2_: B, C, N and F. Catal. Today.

[161] Ananpattarachai J., Boonto Y., Kajitvichyanukul P. (2016). Visible light photocatalytic antibacterial activity of Ni-doped and N-doped TiO_2_ on Staphylococcus aureus and Escherichia coli bacteria. Environ. Sci. Pollut. Res..

[162] Zener B., Matoh L., Carraro G., Miljevic B., Korosec R.C. (2018). Sulfur-, nitrogen- and platinum-doped titania thin films with high catalytic efficiency under visible-light illumination. Beilstein J. Nanotechnol..

[163] Cravanzola S., Cesano F., Gaziano F., Scarano D. (2017). Sulfur-doped TiO_2_: Structure and surface properties. Catalysts.

[164] Banerjee S., Pillai S.C., Falaras P., O’Shea K.E., Byrne J.A., Dionysiou D.D. (2014). New insights into the mechanism of visible light photocatalysis. J. Phys. Chem. Lett..

[165] Yu J.C., Yu J., Ho W., Jiang Z., Zhang L. (2002). Effects of F- doping on the photocatalytic activity and microstructures of nanocrystalline TiO_2_ powders. Chem. Mater..

[166] Asahi R., Morikawa T., Ohwaki T., Aoki K., Taga Y. (2001). Visible-light photocatalysis in nitrogen-doped titanium oxides. Science.

[167] Varley J.B., Janotti A., van de Walle C.G. (2011). Mechanism of visible-light photocatalysis in nitrogen-doped TiO_2_. Adv. Mater..

[168] Ansari S.A., Khan M.M., Ansari M.O., Cho M.H. (2016). Nitrogen-doped titanium dioxide (N-doped TiO_2_) for visible light photocatalysis. New J. Chem..

[169] Yang K., Dai Y., Huang B., Whangbo M.H. (2009). Density functional characterization of the visible-light absorption in substitutional C-anion- and C-cation-doped TiO_2_. J. Phys. Chem. C.

[170] Di Valentin C., Pacchioni G., Selloni A. (2005). Theory of carbon doping of titanium dioxide. Chem. Mater..

[171] Geng H., Yin S., Yang X., Shuai Z., Liu B. (2006). Geometric and electronic structures of the boron-doped photocatalyst TiO_2_. J. Phys. Condens. Matter.

[172] Patel N., Dashora A., Jaiswal R., Fernandes R., Yadav M., Kothari D.C. (2015). Experimental and theoretical investigations on the activity and stability of substitutional and interstitial boron in TiO_2_ photocatalyst. J. Chem. Phys..

[173] Sotelo-Vazquez C., Noor N., Kafizas A., Quesada-Cabrera R., Scanlon D.O., Taylor A., Durrant J.R., Parkin I.P. (2015). Multifunctional P-doped TiO_2_ films: A new approach to self-cleaning, transparent conducting oxide materials. Chem. Mater..

[174] Gopal N.O., Lo H.H., Ke T.F., Chou C.C., Wu J.D., Sheu S.C., Ke S.C. (2012). Visible light active phosphorus-doped TiO_2_ nanoparticles: An EPR evidence for the enhanced charge separation. J. Phys. Chem. C.

[175] Li D., Haneda H., Hishita S., Labhsetwar N.K. (2005). Fluorine-doped TiO_2_ powders prepared by spray pyrolysis and their improved photocatalytic activity for decomposition of gas-phase acetaldehyde. J. Fluor. Chem..

[176] Yang G., Wang T., Yang B., Yan Z., Ding S., Xiao T. (2013). Enhanced visible-light activity of F-N co-doped TiO_2_ nanocrystals via nonmetal impurity, Ti^3+^ ions and oxygen vacancies. Appl. Surf. Sci..

[177] Janczarek M., Endo M., Zhang D., Wang K., Kowalska E. (2018). Enhanced photocatalytic and antimicrobial performance of cuprous oxide/titania: The effect of titania matrix. Materials.

[178] Haque F., Daeneke T., Kalantar Zadeh K., Ou J. (2018). Two-dimensional transition metal oxide and chalcogenide-based photocatalysts. Nano-Micro Lett..

[179] Kiwi J., Rtimi S. (2018). Mechanisms of the antibacterial effects of TiO_2_–FeOx under solar or visible light: Schottky barriers versus surface plasmon resonance. Coatings.

[180] Bera S., Won D.I., Rawal S.B., Kang H.J., Lee W.I. (2019). Design of visible-light photocatalysts by coupling of inorganic semiconductors. Catal. Today.

[181] Phung H.N., Tran V.N., Nguyen L.T., Phan L.K., Duong P.A., Le H.V. (2017). Investigating visible-photocatalytic activity of MoS_2_/TiO_2_ heterostructure thin films at various MoS_2_ deposition times. J. Nanomater..

[182] Liao Y., Deng P., Wang X., Zhang D., Li F., Yang Q., Zhang H., Zhong Z. (2018). A Facile method for preparation of Cu_2_O-TiO_2_ NTA heterojunction with visible-photocatalytic activity. Nanoscale Res. Lett..

[183] Li G., Huang J., Chen J., Deng Z., Huang Q., Liu Z., Guo W., Cao R. (2019). Highly active photocatalyst of Cu_2_O/TiO_2_ octahedron for hydrogen generation. ACS Omega.

[184] Zhu X., Gu P., Wu H., Yang D., Sun H., Wangyang P., Li J., Tian H. (2017). Influence of substrate on structural, morphological and optical properties of TiO_2_ thin films deposited by reaction magnetron sputtering. AIP Adv..

[185] Orlianges J.C., Crunteanu A., Pothier A., Merie-Mejean T., Blondy P., Champeaus C. (2012). Titanium dioxide thin films deposited by pulsed laser deposition and integration in radio frequency devices: Study of structure, optical and dielectric properties. Appl. Surf. Sci..

[186] Vahl A., Veziroglu S., Henkel B., Strunskus T., Polonskyi O., Aktas O.C., Fuapel F. (2019). Pathways to tailor photocatalytic performance of TiO_2_ thin films deposited by reactive magnetron sputtering. Materials.

[187] Johari N.D., Rosli J.M., Juoi J.M., Yazid S.A. (2019). Comparison on the TiO_2_ crystalline phases deposited via dip and spin coating using green sol–gel route. J. Mater. Res. Technol..

[188] Manoj P.K., Koshy P., Vaidyan V.K. (2012). Transparent anatase titania films: A critical study on optical properties. Prog. Nat. Sci. Mater. Int..

[189] Bai Y., Mora-Sero I., De Angelis F., Bisquert J., Wang P. (2014). Titanium dioxide nanomaterials for photovoltaic applications. Chem. Rev..

[190] Rauwel E., Willinger M.G., Ducroquet F., Rauwel P., Matko I., Kiselev D., Pinna N. (2008). Carboxylic acids as oxygen sources for the atomic layer deposition of high-κ metal oxides. J. Phys. Chem. C.

[191] Correa G.C., Bao B., Strandwitz N.C. (2015). Chemical stability of titania and alumina thin films formed by atomic layer deposition. ACS Appl. Mater. Interfaces.

[192] Zhang Y., Utke I., Michler J., Ilari G., Rossell M.D., Erni R. (2014). Growth and characterization of CNT–TiO_2_ heterostructures. Beilstein J. Nanotechnol..

[193] Kumar S.G., Koteswara Rao K.S. (2014). Polymorphic phase transition among the titania crystal structures in solution based approach: From precursor chemistry to nucleation process. Nanoscale.

[194] Padmanabhan S.C., Pillai S.C., Colreavy J., Balakrishnan S., McCormack D.E., Perova T.S., Gunko Y., Hinder S.J., Kelly J.M. (2007). A simple sol gel processing for the development of high-temperature stable photoactive anatase titania. Chem. Mater..

[195] Behnajady M.A., Eskandarloo H. (2015). Preparation of TiO_2_ nanoparticles by the sol–gel method under different pH conditions and modeling of photocatalytic activity by artificial neural network. Res. Chem. Intermed..

[196] Lusvard G., Barani C., Giubertoni F., Paganelli G. (2017). Synthesis and characterization of TiO_2_ nanoparticles for the reduction of water pollutants. Materials.

[197] Sharma M., Pathak M., Kapoor P.N. (2018). The sol-gel method: Pathway to ultrapure and homogeneous mixed metal oxide nanoparticles. Asian J. Chem..

[198] Marami M.B., Farahmandjoum M., Khoshnevisan B. (2018). Sol–gel synthesis of Fe-doped TiO_2_ nanocrystals. J. Electron. Mater..

[199] Mogal S.I., Gandhi V.G., Mishra M., Tripathi S., Joshi P.A., Shah D.O. (2014). Single-step synthesis of silver-doped titanium dioxide: Influence of silver on structural, textural, and photocatalytic properties. Ind. Eng. Chem. Res..

[200] Katoueizadeh E., Zebarjad S.M., Janghorban K. (2018). Synthesis and enhanced visible-light activity of N-doped TiO_2_ nano-additives applied over cotton textiles. J. Mater. Res. Technol..

[201] Nolan T., Synnot D., Seery M., Hider S., Van Wassenhaven A., Pillai S. (2012). Effect of N-doping on the photocatalytic activity of sol-gel TiO_2_. J. Hazard. Mater..

[202] Zane A., Zuo R., Villamena F.A., Rockenbauer A., Foushee A.M., Flores K., Dutta P.K., Nagy A. (2016). Biocompatibility and antibacterial activity of nitrogen-doped titanium dioxide nanoparticles for use in dental resin formulations. Int. J. Nanomed..

[203] Qin H.L., Gu G.B., Liu S. (2008). Preparation of nitrogen-doped titania with visible-light activity and its application. C. R. Chim..

[204] Livraghi S., Chierotti M.R., Giamello E., Magnacca G., Paganini M.C., Cappelletti G., Bianchi C.L. (2008). Nitrogen-doped titanium dioxide active in photocatalytic reactions with visible light: A multi-technique characterization of differently prepared materials. J. Phys. Chem. C.

[205] Yanagisawa K., Ovenstone J. (1999). Crystallization of anatase from amorphous titania using the hydrothermal technique:  Effects of starting material and temperature. J. Phys. Chem. B.

[206] Stride J.A., Tuong N.T., Nowotny M.K., Nowotny J. (2010). Controlled Synthesis of Titanium Dioxide Nanostructures. Solid State Phenomena: Solid State Chemistry and Photocatalysis of Titanium Dioxide.

[207] Morais A., Longo C., Araujo J., Barroso M. (2015). Nanocrystalline anatase TiO_2_/reduced graphene oxide composite films as photoanodes for photoelectrochemical water splitting studies: The role of the reduced graphene oxide. Phys. Chem. Chem. Phys..

[208] De Marco L., Manca M., Giannuzzi R., Malara F., Melcarne G., Ciccarella G., Zama I., Cingolani R., Gigli G. (2010). Novel preparation method of TiO_2_-nanorod-based photoelectrodes for dye-sensitized solar cells with improved light-harvesting efficiency. J. Phys. Chem. C.

[209] Falentin-Daudré C., Baumann J.S., Migonney V., Spadavecchia J. (2017). Highly crystalline sphere and rod-shaped TiO_2_ nanoparticles: A facile route to bio-polymer grafting. Front. Lab. Med..

[210] Roca R.A., Leite E.R. (2013). Size and shape tailoring of titania nanoparticles synthesized by solvothermal route in different solvents. J. Am. Ceram. Soc..

[211] Zhang S., Li Y., Li M. (2018). Facile Synthesis of anatase TiO_2_ nanospheres as anode materials for sodium-ion batteries. JOM.

[212] Dubey R.S., Krishnamurthy K.V., Singh S. (2019). Experimental studies of TiO_2_ nanoparticles synthesized by sol-gel and solvothermal routes for DSSCs application. Results Phys..

[213] Tjong S.C., Hoffman R.W., Yeager E.B. (1982). Electron and ion spectroscopic iron-chromium alloys. J. Electrochem. Soc..

[214] Tjong S.C., Yeager E. (1981). ESCA and SIMS studies of the passive film on iron. J. Electrochem. Soc..

[215] Louarn G., Salou L., Hoonaert A., Layrolle P. (2019). Nanostuctured surface coatings for titanium alloy implants. J. Mater. Res..

[216] Kulkarni M., Mazare A., Gongadze E., Perutkova S., Kralj-Iglič V., Milosev I., Schmuki P., Iglic A., Mozetic M. (2015). Titanium nanostructures for biomedical applications. Nanotechnology.

[217] Minagar S., Wang J., Berndt C.C., Ivanova E.P., Wen C. (2013). Cell response of anodized nanotubes on titanium and titanium alloys. J. Biomed. Mater. Res. Part A.

[218] Su E., Justin D.F., Pratt C.R., Sarin V.K., Nguyen V.S., Oh S., Jin S. (2018). Effects of titanium nanotubes on the osseointegration, cell differentiation, mineralisation and antibacterial properties of orthopaedic implant surfaces. Bone Jt. J..

[219] Fu Y., Mo A. (2018). A Review on the electrochemically self-organized titania nanotube arrays: Synthesis, modifications, and biomedical applications. Nanoscale Res. Lett..

[220] Liu N., Chen X., Zhang J., Schwank J.W. (2014). A review on TiO_2_-based nanotubes synthesized via hydrothermal method: Formation mechanism, structure modification, and photocatalytic applications. Catal. Today.

[221] Ahmad A., Haq E.U., Akhtar W., Arshad M., Ahmad Z. (2017). Synthesis and characterization of titania nanotubes by anodizing of titanium in fluoride containing electrolytes. Appl. Nanosci..

[222] Macak J.M., Tsuchiya H., Ghicov A., Yasuda K., Hahn R., Bauer S., Schmuki P. (2007). TiO_2_ nanotubes: Self-organized electrochemical formation, properties and applications. Curr. Opin. Solid State Mater. Sci..

[223] Do T.C., Nguyen T.Q., Nguyen K.T., Le P.H. (2019). TiO_2_ and Au-TiO_2_ nanomaterials for rapid photocatalytic degradation of antibiotic residues in aquaculture wastewater. Materials.

[224] Lai C.W. (2015). Surface morphology and growth of anodic titania nanotubes films: Photoelectrochemical water splitting studies. J. Nanomater..

[225] Nguyen T.L., Ung T.D., Nguyen Q.L. (2014). Non-chapped, vertically well aligned titanium dioxide nanotubes fabricated by electrochemical etching. Adv. Nat. Sci. Nanosci. Nanotechnol..

[226] Lan M.Y., Liu C.P., Huang H.H., Lee S.W. (2013). Both enhanced biocompatibility and antibacterial activity in Ag-decorated TiO_2_ nanotubes. PLoS ONE.

[227] Li Y., Liao C., Tjong S.C. (2019). Electrospun polyvinylidene fluoride-based fibrous scaffolds with piezoelectric characteristics for bone and neural tissue engineering. Nanomaterials.

[228] Al-Enizi A.M., Zagho M.M., Elzatahry A.A. (2018). Polymer-based electrospun nanofibers for biomedical applications. Nanomaterials.

[229] Feng S., Zhang F., Ahmed S., Liu Y. (2019). Physico-mechanical and antibacterial properties of PLA/TiO_2_ composite materials synthesized via electrospinning and solution casting processes. Coatings.

[230] Xue J., Wu T., Dai Y., Xia Y. (2019). Electrospinning and electrospun nanofibers: Methods, materials, and applications. Chem. Rev..

[231] Tekmen C., Susio A., Cocen U. (2008). Titania nanofibers prepared by electrospinning. Mater. Lett..

[232] Mondal K. (2017). Recent advances in the synthesis of metal oxide nanofibers and their environmental remediation applications. Inventions.

[233] Albetran H., O’Connor B.H., Low I.M. (2016). Effect of calcination on band gaps for electrospun titania nanofibers heated in air–argon mixtures. Mater. Des..

[234] Chapman B.S., Mishra S.R., Tracy J.B. (2019). Direct electrospinning of titania nanofibers with ethanol. Dalton Trans..

[235] Pan X., Yang M.Q., Fu X., Zhang N. (2013). Defective TiO_2_ with oxygen vacancies: Synthesis, properties and photocatalytic applications. Nanoscale.

[236] Nasr M., Balme S., Eid C., Habchi R., Miele P., Bechelany M. (2017). Enhanced visible-light photocatalytic performance of electrospun rGO/TiO_2_ composite nanofibers. J. Phys. Chem. C.

[237] Kiwi J., Rtimi S., Sanjines R., Pulgarin C. (2014). TiO_2_ and TiO_2_-doped films able to kill bacteria by contact: New evidence for the dynamics of bacterial inactivation in the dark and under light irradiation. Int. J. Photoenergy.

[238] Li Y., Zhang W., Niu J., Chen Y. (2012). Mechanism of photogenerated reactive oxygen species and correlation with the antibacterial properties of engineered metal-oxide nanoparticles. ACS Nano.

[239] Tsai T.M., Chang H.H., Chang K.C., Liu Y.L., Tseng C.C. (2010). A comparative study of the bactericidal effect of photocatalytic oxidation by TiO_2_ on antibiotic-resistant and antibiotic-sensitive bacteria. J. Chem. Technol. Biotechnol..

[240] Kubacka A., Diez M.S., Rojo D., Bargiela R., Ciordia S., Zapico I., Albar J.P., Barbas C., dos Santos V.A., Fernández-García M. (2015). Understanding the antimicrobial mechanism of TiO_2_-based nanocomposite films in a pathogenic bacterium. Sci. Rep..

[241] Vatansever F., de Melo W.C., Avci P., Vecchio D., Sadasivam M., Gupta A., Chandran R., Karimi M., Parizotto N.A., Yin R. (2013). Antimicrobial strategies centered around reactive oxygen species—Bactericidal antibiotics, photodynamic therapy and beyond. FEMS Microbiol. Rev..

[242] Sheng H., Nakamura K., Kanno T., Sasaki K., Niwano Y. (2015). Bactericidal effect of photolysis of H_2_O_2_ in combination with sonolysis of water via hydroxyl radical generation. PLoS ONE.

[243] Michels H.T., Keevil C.W., Salgado C.D., Schmidt M.G. (2015). From laboratory research to a clinical trial: Copper alloy surfaces kill bacteria and reduce hospital-acquired infections. Herd.

[244] Rtimi S., Pulgarin C., Kiwi J. (2017). Recent developments in accelerated antibacterial inactivation on 2D Cu-titania surfaces under indoor visible light. Coatings.

[245] Moongraksathum B., Shang J.Y., Chen Y.W. (2018). Photocatalytic antibacterial effectiveness of Cu-doped TiO_2_ thin film prepared via the peroxo sol-gel method. Catalysts.

[246] Leyland N.S., Podporska-Carroll J., Browne J., Hinder S.J., Quilty B., Pillai S.C. (2016). Highly efficient F, Cu doped TiO_2_ anti-bacterial visible light active photocatalytic coatings to combat hospital-acquired infections. Sci. Rep..

[247] Yadav H.M., Odari S.V., Koli V.B., Mali S.S., Hong C.K., Pawar S.H., Delekar S.D. (2014). Preparation and characterization of copper-doped anatase TiO_2_ nanoparticles with visible light photocatalytic antibacterial activity. J. Photochem. Photobiol. A.

[248] Yadav H.M., Odari S.V., Bohara R.A., Mali S.S., Pawar S.H., Delekar S.D. (2014). Synthesis and visible light photocatalytic antibacterial activity of nickel-doped TiO_2_ nanoparticles against Gram-positive and Gram-negative bacteria. J. Photochem. Photobiol. A.

[249] Vollmer W., Blanot D., De Pedro M.A. (2008). Peptidoglycan structure and architecture. FEMS Microbiol. Rev..

[250] Bertani B., Ruiz N. (2018). Function and biogenesis of lipopolysaccharides. EcoSal Plus.

[251] Botos I., Noinaj N., Buchanan S.K. (2017). Insertion of proteins and lipopolysaccharide into the bacterial outer membrane. Philos. Trans. R. Soc. B.

[252] Polissi A., Sperandeo P. (2014). The lipopolysaccharide export pathway in Escherichia coli: Structure, organization and regulated assembly of the Lpt machinery. Mar. Drugs.

[253] Dakal T.C., Kumar A., Majumdar R.S., Yadav V. (2016). Mechanistic basis of antimicrobial actions of silver nanoparticles. Front. Microbiol..

[254] Hsueh Y.H., Lin K.S., Ke W.J., Hsieh C.T., Chiang C.L., Tzou D.Y., Liu S.T. (2015). The antimicrobial properties of silver nanoparticles in Bacillus subtilis are mediated by released Ag^+^ ions. PLoS ONE.

[255] Riaz Ahmed K.B., Nagy A.M., Brown R.P., Zhang Q., Malghan S.G., Goering P.L. (2017). Silver nanoparticles: Significance of physicochemical properties and assay interference on the interpretation of in vitro cytotoxicity studies. Toxicol. In Vitro.

[256] Gupta K., Singh R.P., Pandey A., Pandey A. (2013). Photocatalytic antibacterial performance of TiO_2_ and Ag-doped TiO_2_ against *S. aureus*, *P. aeruginosa* and *E. coli*. Beilstein J. Nanotechnol..

[257] Garvey M., Panaitescu E., Menon L., Byrne C., Dervin S., Hinder S.J., Pillai S.C. (2016). Titania nanotube photocatalysts for effectively treating waterborne microbial pathogens. J. Catal..

[258] Nguyen N.T., Ozkan S., Tomanec O., Zboril A., Schmuki P. (2018). Spaced titania nanotube arrays allow the construction of an efficient N-doped hierarchical structure for visible light harvesting. ChemistryOpen.

[259] Podporska-Carroll J., Panaitescu E., Quilty B., Wang L., Menon L., Pillai S.C. (2015). Antimicrobial properties of highly efficient photocatalytic TiO_2_ nanotubes. Appl. Catal. B Environ..

[260] Hajjaji A., Elabidi M., Trabelsi K., Assadi A.A., Bessais B., Rtimi S. (2018). Bacterial adhesion and inactivation on Ag decorated TiO_2_-nanotubes under visible light: Effect of the nanotubes geometry on the photocatalytic activity. Colloids Surf. B.

[261] Uhm S.H., Song D.H., Kwon J.S., Lee S.B., Han J.G., Kim K.N. (2014). Tailoring of antibacterial Ag nanostructures on TiO_2_ nanotube layers by magnetron sputtering. J. Biomed. Mater. Res. Part B.

[262] Dunnill C.W., Aiken Z.A., Kafizas A., Pratten J., Wilson M., Morgan D.J., Parkin I.P. (2009). White light induced photocatalytic activity of sulfur-doped TiO_2_ thin films and their potential for antibacterial application. J. Mater. Chem..

[263] Xue X., Wang Y., Yang H. (2013). Preparation and characterization of boron-doped titania nano-materials with antibacterial activity. Appl. Surf. Sci..

[264] Sun D.S., Kau J.H., Huang H.H., Tseng Y.H., Wu W.S., Chang H.H. (2016). Antibacterial properties of visible-light-responsive carbon-containing titanium dioxide photocatalytic nanoparticles against anthrax. Nanomaterials.

[265] He P., Tao J., Huang X., Xue J. (2013). Preparation and photocatalytic antibacterial property of nitrogen doped TiO_2_ nanoparticles. J. Sol-Gel Sci. Technol..

[266] Fagan R., McCormack D.E., Hinder S., Pillai S.C. (2016). Improved high temperature stability of anatase TiO_2_ photocatalysts by N, F, P co-doping. Mater. Des..

[267] Li C., Sun Z., Ma R., Xue Y., Zheng S. (2017). Fluorine doped anatase TiO_2_ with exposed reactive (001) facets supported on porous diatomite for enhanced visible-light photocatalytic activity. Microporous Mesoporous Mater..

[268] Hamilton J.W.J., Byrne J.A., Dunlop P.S.M., Dionysiou D.D., Pelaez M., O’Shea K., Synnott D., Pillai S.C. (2014). Evaluating the mechanism of visible light activity for N,F-TiO_2_ using photoelectrochemistry. J. Phys. Chem. C.

[269] Abdullah A.M., Gracia-Pinilla M.A., Pillai S.C., O’Shea K. (2019). UV and visible light-driven production of hydroxyl radicals by reduced forms of N, F, and P codoped titanium dioxide. Molecules.

[270] Milosevic I., Jayaprakash A., Greenwood B., van Driel B., Rtimi S., Bowen P. (2017). Synergistic effect of fluorinated and N doped TiO_2_ nanoparticles leading to different microstructure and enhanced photocatalytic bacterial inactivation. Nanomaterials.

[271] Milosevic I., Rtimi S., Jayaprakash A., van Driel B., Greenwood B., Aimable A., Senna M., Bowen P. (2018). Synthesis and characterization of fluorinated anatase nanoparticles and subsequent N-doping for efficient visible light activated photocatalysis. Colloids Surf. B.

[272] Akhavan O., Ghaderi E. (2010). Toxicity of graphene and graphene oxide nanowalls against bacteria. ACS Nano.

[273] Akhavan O., Ghaderi E., Esfandiar A. (2011). Wrapping bacteria by graphene nanosheets for isolation from environment, reactivation by sonication and inactivation by near-infrared irradiation. J. Phys. Chem. B.

[274] Lu X., Feng X., Werber J.R., Chu C., Zucker I., Kim J.H., Osuji J.O., Elimelech M. (2017). Enhanced antibacterial activity through the controlled alignment of graphene oxide nanosheets. Proc. Natl. Acad. Sci. USA.

[275] Linklater D.P., Baulin V.A., Juodkazis S., Ivanova E.P. (2018). Mechano-bactericidal mechanism of graphene nanomaterials. Interface Focus.

[276] Akhavan O., Ghaderi E. (2009). Photocatalytic reduction of graphene oxide nanosheets on TiO_2_ thin film for photoinactivation of bacteria in solar light irradiation. J. Phys. Chem. C.

[277] Nica I.C., Stan M.S., Popa M., Chifiriuc M.C., Pircalabioru G.G., Lazar V., Dumitrescu I., Diamandescu L., Feder M., Baibarac M. (2017). Development and biocompatibility evaluation of photocatalytic TiO_2_/reduced graphene oxide-based nanoparticles designed for self-cleaning purposes. Nanomaterials.

[278] Andrews J.M. (2002). Determination of minimum inhibitory concentration. J. Antimicrob. Chemother..

[279] Macia M.D., Rojo-Molinero E., Oliver A. (2014). Antimicrobial susceptibility testing in biofilm-growing bacteria. Clin. Microbiol. Infect..

[280] Tjong S.C. (2006). Structural and mechanical properties of polymer nanocomposites. Mater. Sci. Eng. R Rep..

[281] Jamróz E., Kulawik P., Kopel P. (2019). The effect of nanofillers on the functional properties of biopolymer-based films: A review. Polymers.

[282] Gao T., Jiang M., Liu X., You G., Wang W., Sun Z., Ma A., Chen J. (2019). Patterned polyvinyl alcohol hydrogel dressings with stem cells seeded for wound healing. Polymers.

[283] Mochane M.J., Motsoeneng T.S., Sadiku E.R., Mokhena T.C., Sefadi J.S. (2019). Morphology and properties of electrospun PCL and its composites for medical applications: A mini review. Appl. Sci..

[284] Liao C., Li Y., Tjong S.C. (2019). Antibacterial activities of aliphatic polyester nanocomposites with silver nanoparticles and/or graphene oxide sheets. Nanomaterials.

[285] Rescek A., Scetar M., Hrnjak-Murgić Z., Dimitrov N., Galic K. (2016). Polyethylene/polycaprolactone nanocomposite films for food Packaging modified with magnetite and casein: Oxygen barrier, mechanical, and thermal properties. Polym. Plast. Technol..

[286] Xing Y., Li X., Zhang L., Xu Q., Che Z., Li W., Bai Y., Li K. (2012). Effect of TiO_2_ nanoparticles on the antibacterial and physical properties of polyethylene-based film. Prog. Org. Coat..

[287] Munoz-Bonilla A., Cerrada M.L., Fernández-Garcia M., Kubacla A., Ferrer M., Fernandez-Garcia M. (2013). Biodegradable polycaprolactone-titania nanocomposites: Preparation, characterization and antimicrobial properties. Int. J. Mol. Sci..

[288] Raut A.V., Yadav H.M., Gnanamani A., Pushpavanam S., Pawar S.H. (2011). Synthesis and characterization of chitosan-TiO_2_: Cu nanocomposite and their enhanced antimicrobial activity with visible light. Colloids Surf. B.

[289] Zhang X., Xiao G., Wang Y., Zhao Y., Su H., Tan T. (2017). Preparation of chitosan-TiO_2_ composite film with efficient antimicrobial activities under visible light for food packaging applications. Carbohydr. Polym..

[290] Li J., Xie B., Xia K., Li Y., Han J., Zhao C. (2018). Enhanced antibacterial activity of silver doped titanium dioxide-chitosan composites under visible light. Materials.

[291] Jbeli A., Hamden Z., Bouattour S., Ferraria A.M., Conceicao D.S., Vieira Ferreira L.F., Chehimi M.M., do Rego A.M., Rei Vilar M., Boufi S. (2018). Chitosan-Ag-TiO_2_ films: An effective photocatalyst under visible light. Carbohydr. Polym..

[292] Saravanan R., Aviles J., Gracia F., Mosquera E., Gupta V.K. (2018). Crystallinity and lowering band gap induced visible light photocatalytic activity of TiO_2_/CS (chitosan) nanocomposites. Int. J. Biol. Macromol..

[293] Zhao Y., Tao C., Xiao G., Xu H. (2017). Controlled synthesis and wastewater treatment of Ag_2_O/TiO_2_ modified chitosan-based photocatalytic film. RSC Adv..

[294] Hamden Z., Bouattour S., Ferraria A.M., Ferreira D.P., Vieira Ferreira L.F., Botelho do Rego A.M., Boufi S. (2016). In situ generation of TiO_2_ nanoparticles using chitosan as a template and their photocatalytic activity. J. Photochem. Photobiol. A.

[295] Kaewklin P., Siripatrawan U., Suwanagul A., Lee Y.S. (2018). Active packaging from chitosan-titanium dioxide nanocomposite film for prolonging storage life of tomato fruit. Int. J. Biol. Macromol..

[296] Lavengood S.L., Zhang M. (2014). Chitosan-based scaffolds for bone tissue engineering. J. Mater. Chem. B.

[297] Callewaert C., De Maeseneire E., Kerckhof F.M., Verliefde A., Van de Wiele T., Boon N. (2014). Microbial odor profile of polyester and cotton clothes after a fitness session. Appl. Environ. Microbiol..

[298] Zahid M., Papadopoulou E.L., Suarato G., Binas V.D., Kiriakidis G., Gounaki I., Moira O., Venieri D., Bayer I.S., Athanassiou A. (2018). Fabrication of visible light-induced antibacterial and self-cleaning cotton fabrics using manganese doped TiO_2_ nanoparticles. ACS Appl. Bio Mater..

[299] Pfang P.G., García-Cañete J., García-Lasheras J., Blanco A., Aunon A., Parron-Cambero R., Macías-Valcayo A., Esteban J. (2019). Orthopedic implant-associated infection by multidrug resistant Enterobacteriaceae. J. Clin. Med..

[300] Li Y., Yang Y., Li R., Tang X., Guo D., Qing Y., Qin Y. (2019). Enhanced antibacterial properties of orthopedic implants by titanium nanotube surface modification: A review of current techniques. Int. J. Nanomed..

[301] Gollwitzer H., Haenie M., Mittelmeier W., Heidenau F., Harrasser N. (2018). A biocompatible sol-gel derived titania coating for medical implants with antibacterial modification by copper integration. AMB Express.

[302] Tsou H.K., Hsieh P.Y., Chi M.H., Chung C.J., He J.L. (2012). Improved osteoblast compatibility of medical-grade polyetheretherketone using arc ionplated rutile/anatase titanium dioxide films for spinal implants. J. Biomed. Mater. Res. Part A.

[303] Radtke A., Topolski A., Jędrzejewski T., Kozak W., Sadowska B., Więckowska-Szakiel M., Szubka M., Talik E., Nielsen L.P., Piszczek P. (2017). The bioactivity and photocatalytic properties of titania nanotube coatings produced with the use of the low-potential anodization of Ti6Al4V alloy surface. Nanomaterials.

[304] Xu L., Lv K., Yu W.Q. (2016). Effect of TiO_2_ nanotube layers thickness on periodontal ligament cells. Dentistry.

[305] Piszczek P., Lewandowska Z., Radtke A., Jędrzejewski T., Kozak W., Sadowska B., Szubka M., Talik E., Flori F. (2017). Biocompatibility of titania nanotube coatings enriched with silver nanograins by chemical vapor deposition. Nanomaterials.

[306] Laux P., Tentschert J., Riebeling C., Braeuning A., Creutzenberg O., Epp A., Fessard V., Haas K.H., Haase A., Hund-Rinke K. (2018). Nanomaterials: Certain aspects of application, risk assessment and risk communication. Arch. Toxicol..

[307] Wadhwa S., Rea C., O’Hare P., Mathur A., Roy S.S., Dunlop P.S.M., Byrne J.A., Burke G., Meenan B., McLaughlin J.A. (2011). Comparative in vitro cytotoxicity study of carbon nanotubes and titania nanostructures on human lung epithelial cells. J. Hazard. Mater..

[308] Mohamed M.A., Torabi A., Paulose M., Sakthi Kumar D., Varghese O.K. (2017). Anodically grown titania nanotube induced cytotoxicity has genotoxic origins. Sci. Rep..

[309] Allegri M., Bianchi M.G., Chiu M., Varet J., Costa A.L., Ortelli S., Blosi M., Bussolati O., Poland C.A., Bergamaschi E. (2016). Shape-related toxicity of titanium dioxide nanofibres. PLoS ONE.

[310] Holden P.A., Gardea-Torresdey J.L., Klaessig F., Turco R., Mortimer M., Hund-Rinke K., Avery D., Barcelo D., Behra R., Cohen Y. (2016). Considerations of environmentally relevant test conditions for improved evaluation of ecological hazards of engineered nanomaterials. Environ. Sci. Technol..

[311] Gupta R., Xie H. (2018). Nanoparticles in daily life: Applications, toxicity and regulations. J. Environ. Pathol. Toxicol. Oncol..

[312] Khezri S.M., Shariat S.M., Tabibian S. (2013). Evaluation of extracting titanium dioxide from water-based paint sludge in auto-manufacturing industries and its application in paint production. Toxicol. Ind. Health.

[313] Al-Kattan A., Wichser A., Vonbank R., Brunner S., Ulrich A., Zuin S., Nowack B. (2013). Release of TiO_2_ from paints containing pigment-TiO_2_ or nano-TiO_2_ by weathering. Environ. Sci. Process. Impacts.

[314] Kim Y. (2014). Nanowastes treatment in environmental media. Environ. Health Toxicol..

[315] Shi X., Li Z., Chen W., Qiang L., Xia J., Chen M., Zhu L., Alvarez P.J. (2016). Fate of TiO_2_ nanoparticles entering sewage treatment plants and bioaccumulation in fish in the receiving streams. NanoImpact.

[316] Clemente Z., Castro V.L., Moura M.A., Jonsson C.M., Fraceto L.F. (2014). Toxicity assessment of TiO_2_ nanoparticles in zebrafish embryos under different exposure conditions. Aquat. Toxicol..

[317] De Matteis V. (2017). Exposure to inorganic nanoparticles: Routes of entry, immune response, biodistribution and in vitro/in vivo toxicity evaluation. Toxics.

[318] Ahamed M., Khan M.A.M., Akhtar M.J., Alhadlaq H.A., Alshamshan A. (2017). Ag-doping regulates the cytotoxicity of TiO_2_ nanoparticles via oxidative stress in human cancer cells. Sci. Rep..

[319] Kuku G., Culha M. (2017). Investigating the origins of toxic response in TiO_2_ nanoparticle-treated cells. Nanomaterials.

[320] Batt J., Milward M., Chapple I., Grant M., Roberts H., Addison O. (2018). TiO_2_ nanoparticles can selectively bind CXCL8 impacting on neutrophil chemotaxis. Eur. Cells Mater..

[321] Ribeiro A., Gemini-Piperni S., Travassos R., Lemgruber L., Silva R.C., Rossi A.L., Farina M., Anselme K., Shokuhfar T., Shahbazian-Yassar R. (2016). Trojan-like internalization of anatase titanium dioxide nanoparticles by human osteoblast cells. Sci. Rep..

[322] Valentini X., Absil L., Laurent G., Robbe A., Laurent S., Muller R., Legrand A., Nonclercq D. (2017). Toxicity of TiO_2_ nanoparticles on the NRK52E renal cell line. Mol. Cell. Toxicol..

[323] Wang Y., Cui H., Zhou J., Li F., Wang J., Chen M., Liu Q. (2014). Cytotoxicity, DNA damage, and apoptosis induced by titanium dioxide nanoparticles in human non-small cell lung cancer A549 cells. Environ. Sci. Pollut. Res..

[324] Shi H., Magaye R., Castranova V., Zhao J. (2013). Titanium dioxide nanoparticles: A review of current toxicological data. Part. Fibre Toxicol..

[325] Mottola F., Iovine C., Santonastaso M., Romeo M.L., Pacifico S., Cobellis L., Rocco L. (2019). NPs-TiO_2_ and lincomycin coexposure induces DNA damage in cultured human amniotic cells. Nanomaterials.

[326] Golbamak N., Rasulev B., Cassano A., Marchese Robinson R.L., Benfenati E., Leszczynski J., Cronin M.T. (2015). Genotoxicity of metal oxide nanomaterials: Review of recent data and discussion of possible mechanisms. Nanoscale.

[327] Huerta-García E., Zepeda-Quiroz I., Sánchez-Barrera H., Colín-Val Z., Alfaro-Moreno E., Ramos-Godinez M.D.P., López-Marure R. (2018). Internalization of titanium dioxide nanoparticles is cytotoxic for H9c2 rat cardiomyoblasts. Molecules.

[328] Yin J.J., Liu J., Ehrenshaft M., Roberts J.E., Fu P.P., Mason R.P., Zhao B. (2012). Phototoxicity of nanotitanium dioxides in HaCaT keratinocytes—Generation of reactive oxygen species and cell damage. Toxicol. Appl. Pharmacol..

[329] Ren Y., Liu X., Geng R., Lu Q., Rao R., Tan X., Yang X., Liu W. (2018). Increased level of α2,6-sialylated glycans on HaCaT cells induced by titanium dioxide nanoparticles under UV radiation. Nanomaterials.

[330] Grassian V.H., O’Shaughnessy P.T., Adamcakova-Dodd A., Pettibone J.M., Thorne P.S. (2007). Inhalation exposure study of titanium dioxide nanoparticles with a primary particle size of 2 to 5 nm. Environ. Health Perspect..

[331] Liu R., Yin L., Pu Y., Lian G., Zhang J., Su Y., Xia Z., Ye B. (2009). Pulmonary toxicity induced by three forms of titanium dioxide nanoparticles via intratracheal instillation in rats. Prog. Nat. Sci..

[332] Wu J., Liu W., Xue C., Zhou S., Lan F., Bi L., Xu H., Yang X., Zeng F. (2009). Toxicity and penetration of TiO_2_ nanoparticles in hairless mice and porcine skin after subchronic dermal exposure. Toxicol. Lett..

[333] Disdier C., Devoy J., Cosnefroy A., Chalansonnet M., Herlin-Boime N., Brun E., Lund A., Mabondzo A. (2015). Tissue biodistribution of intravenously administrated titanium dioxide nanoparticles revealed blood-brain barrier clearance and brain inflammation in rat. Part. Fibre Toxicol..

[334] Hong J., Zhang Y.Q. (2016). Murine liver damage caused by exposure to nano-titanium dioxide. Nanotechnology.

[335] Jia X., Wang S., Zhou L., Sun L. (2017). The potential liver, brain, and embryo toxicity of titanium dioxide nanoparticles on mice. Nanoscale Res. Lett..

[336] Jin C., Wang F., Tang Y., Zhang X., Wang J., Yang Y. (2014). Distribution of graphene oxide and TiO_2_-graphene oxide composite in A549 cells. Biol. Trace Elem. Res..

[337] Prakash J., Venkatesan M., Praksash J.S., Bharath G., Anwer S., Veluswamy P., Prema D., Venkataprasanna K.S., Venkatasubbu G.D. (2019). Investigations on the in-vivo toxicity analysis of reduced graphene oxide/TiO_2_ nanocomposite in zebrafish embryo and larvae (Danio rerio). Appl. Surf. Sci..

[338] Code of Federal Regulations (Annual Edition). https://www.govinfo.gov/app/collection/cfr.

[339] European Food Safety Authority (EFSA) (2016). Re-evaluation of titanium dioxide (E 171) as a food additive. EFSA J..

[340] Rompelberg C., Heringa M.B., van Donkersgoed G., Drijvers J., Roos A., Westenbrink S., Peters R., van Bemmel G., brand W., Oomen A.G. (2016). Oral intake of added titanium dioxide and its nanofraction from food products, food supplements and toothpaste by the Dutch population. Nanotoxicology.

[341] Hwang J.S., Yu J., Kim H.M., Oh J.M., Choi S.J. (2019). Food additive titanium dioxide and its fate in commercial foods. Nanomaterials.

[342] Skocaj M., Filipic M., Petkovic J., Novak S. (2011). Titanium dioxide in our everyday life; is it safe?. Radiol. Oncol..

[343] Heringa M.B., Peters R.J.B., Bleys R.L.A., van der Lee M.K., Tromp P.C., van Kesteren P.C., van Eijkeren J.C., Undas A.K., Oomen A.G., Bouwmeester H. (2018). Detection of titanium particles in human liver and spleen and possible health implications. Part. Fibre Toxicol..

[344] Kalinska A., Jaworski S., Wierzbicki M., Golebiewski M. (2019). Silver and copper nanoparticles—An alternative in future mastitis treatment and prevention?. Int. J. Mol. Sci..

[345] Li S.H., Zhu T.X., Huang J.Y., Guo Q.Q., Chen G.Q., Lai Y.K. (2017). Durable antibacterial and UV-protective Ag/TiO_2_@fabrics for sustainable biomedical application. Int. J. Nanomed..

[346] Lammel T., Sturve J. (2018). Assessment of titanium dioxide nanoparticle toxicity in the rainbow trout (*Onchorynchus mykiss*) liver and gill cell lines RTL-W1 and RTgill-W1 under particular consideration of nanoparticle stability and interference with fluorometric assays. NanoImpact.

[347] Smith M.R., Fernandes J., Go Y. (2017). Redox dynamics of manganese as a mitochondrial life-death switch. Biochem. Biophys. Res. Commun..

[348] Zhang L., Sang H., Liu Y., Li J. (2013). Manganese activates caspase-9-dependent apoptosis in human bronchial epithelial cells. Hum. Exp. Toxicol..

